# Automated grindstone chemistry: a simple and facile way for PEG-assisted stoichiometry-controlled halogenation of phenols and anilines using *N*-halosuccinimides

**DOI:** 10.3762/bjoc.18.100

**Published:** 2022-08-09

**Authors:** Dharmendra Das, Akhil A Bhosle, Amrita Chatterjee, Mainak Banerjee

**Affiliations:** 1 Department of Chemistry, BITS Pilani, K. K. Birla Goa Campus, Goa 403 726, India

**Keywords:** automated grinding, chemoselectivity, mechanochemistry, *N*-bromosuccinimide, PEG-400, regioselectivity, stoichiometry-controlled halogenation

## Abstract

A simple electrical mortar–pestle was used for the development of a green and facile mechanochemical route for the catalyst-free halogenation of phenols and anilines via liquid-assisted grinding using PEG-400 as the grinding auxiliary. A series of mono-, di-, and tri-halogenated phenols and anilines was synthesized in good to excellent yields within 10–15 min in a chemoselective manner by controlling the stoichiometry of *N*-halosuccinimides (NXS, X = Br, I, and Cl). It was observed that PEG-400 plays a key role in controlling the reactivity of the substrates and to afford better regioselectivity. Almost exclusive *para*-selectivity was observed for the aromatic substrates with free *o-* and *p*-positions for mono- and dihalogenations. As known, the decarboxylation (or desulfonation) was observed in the case of salicylic acids and anthranilic acids (or sulfanilic acids) leading to 2,4,6-trihalogenated products when 3 equiv of NXS was used. Simple instrumentation, metal-free approach, cost-effectiveness, atom economy, short reaction time, and mild reaction conditions are a few noticeable merits of this environmentally sustainable mechanochemical protocol.

## Introduction

**Figure 1:**
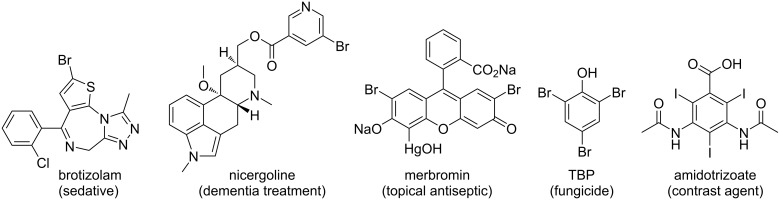
Representative examples of important halogen-containing aryl derivatives.

Aryl halides are valuable compounds with potent bioactivities [1–5] (Figure 1) and are utilized as crucial precursors for various metal-catalyzed cross-coupling reactions [6–9]. They are frequently used as synthetic intermediates in several value-added syntheses of natural products, pharmaceuticals, agrochemicals, and advanced materials [10–14]. The ubiquity of halogen atoms in these synthetic building blocks urges the development of efficient, sustainable, and mild methods for aromatic halogenation.

**Scheme 1:**
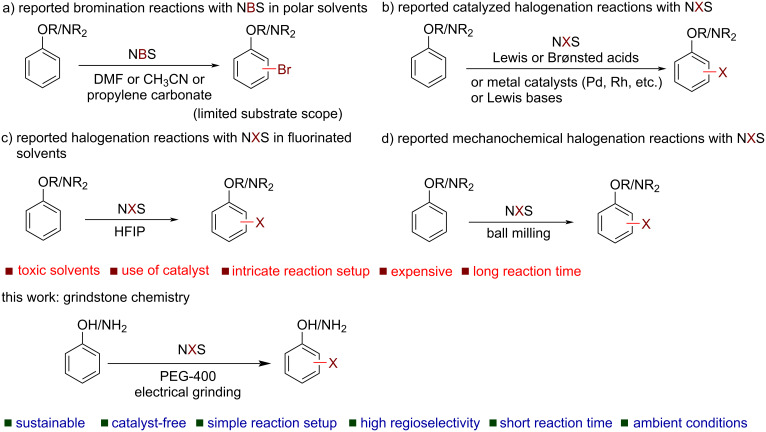
Strategies for halogenation of aromatic compounds using NXS.

The century-old classical method of using hazardous and corrosive reagents X_2_ (X = Br, Cl) suffers from low atom economy (<50%), formation of corrosive byproducts (e.g., HBr) [15–16], which cause serious environmental issues. To mitigate the problem, several mild and operationally safe halogenating agents have been successfully introduced to replace X_2_ [17–31]. Among them, the use of *N*-halosuccinimides has turned out to be a viable alternative to X_2_ because of their low-cost, ease of handling, and possible recycling of the byproduct succinimide [24–31]. In several earlier cases, the bromination with *N*-bromosuccinimide (NBS) was carried out in toxic polar solvents (e.g., DMF), but no iodinated or chlorinated products were obtained because of the low reactivity of NIS and NCS (Scheme 1a) [24–27]. In recent time, the use of Lewis or Brønsted acids, Lewis bases, and transition-metal catalysts (Pd, Rh, Fe, etc.) were employed to boost the reactivity of NXS (Scheme 1b) [32–43]. However, the use of toxic and expensive metals, high catalyst loading, and heating conditions are some sheer hurdles to achieving sustainability. Among notable other catalyst-free methods, the use of costly and low boiling hexafluoroisopropanol (HFIP) as solvent offered the *para*-selective halogenation of activated aromatic systems (Scheme 1c) [44]. It is noteworthy to mention that over-halogenation of activated systems like phenols and anilines, due to the high reactivity and availability of multiple sites for substitution, often leads to an inseparable mixture of halogenated products [27–28]. Thus, a cheaper and sustainable method for a regioselective halogenation in a controlled manner is a worthy pursuit.

**Scheme 2:**
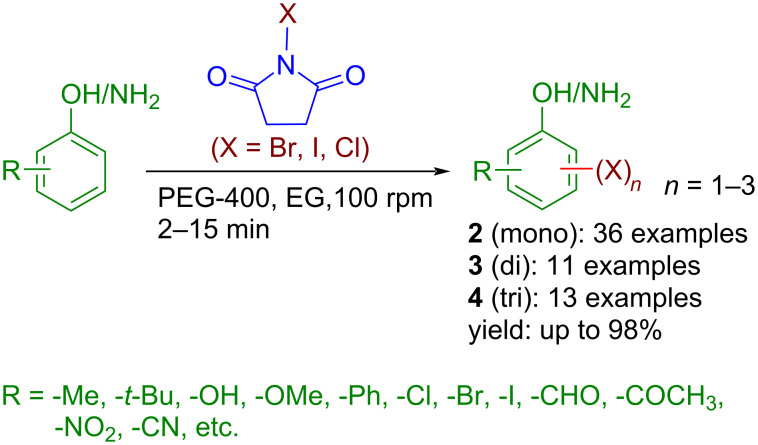
General scheme of PEG-400-assisted halogenation of phenols and anilines in an automated grinder using NXS.

In recent times, mechanochemistry [45–46], achieved by mechanical grinding or milling, has garnered massive interest among chemists owing to its green attributes like solvent-free, clean, atom economic, and time-efficient, and has been identified by the IUPAC as one of 10 world-changing technologies [47]. While milling has received more focus, the simpler form of mechanochemistry, Toda et al.’s “grindstone chemistry” [48] has also been proved as a useful technique for various organic transformations [49]. It is generally carried out by hand-grinding which is not only a labor-intensive process but also raises some concerns on the reaction kinetics, reproducibility, and scalability. An alternative and efficient way of grinding is the use of automation instead of manual intervention. Notably, an industrial-scale synthesis is possible by the suitable choice of a large automated grindstone apparatus. However, there are only limited examples of the use of automated grindstone chemistry [50]. Notably, a few mechanochemical methods are available for aromatic halogenation using NXS (Scheme 1d) [51–53]. However, the solvent-free protocol reported by Mal and co-workers requires several hours for halogenation [51], whereas Ghafuri and co-workers’ method requires the use of a solid acid catalyst [52], apart from the use of high-cost, high-end milling equipment which limits to laboratory scale only. Therefore, developing an operationally simple, environmentally benign protocol, potentially useful for the batch-scale synthesis of aryl halides is highly desirable. From our past experience, we realized a liquid-assisted grinding expedites a reaction to several folds [54–56]. In this regard, PEG-400 is widely preferred due to its biodegradable and benign nature and often offers excellent outcomes where other grinding auxiliaries failed to deliver [56–57]. Herein, we report a sustainable and facile aromatic (mono-, di-, and tri-) halogenation protocol by controlling the stoichiometry of the *N*-halosuccinimide and PEG-400 as the grinding auxiliary in an electrical grinder (Scheme 2).

## Results and Discussion

At the outset, the optimization of the reaction conditions was carried out using *p*-cresol (**1a**) as the model substrate with 1.1 equiv of *N*-bromosuccinimide (NBS) for attempted monobromination. They were ground together at the speed of 100 rpm in an electrical grinder (EG) of Agate-made under neat conditions for 30 min. The reaction was incomplete and a mixture containing some starting material (**1a**), two other spots which are identified as *o*-monobromo (**2a**) as major (57%), and *o*-dibrominated *p*-cresol (**3a**) as minor (20%) constituents, was obtained (Table 1, entry 1). Next, the reaction mixture was ground under LAG conditions with ethanol and an improved yield (77%) of the monobromo product **2a,** with a reduced amount of **3a** (12%), and a nominal recovery of the starting material were observed (Table 1, entry 2). Incomplete consumption of starting phenol **1a** was primarily due to over bromination. The LAG in the presence of water afforded relatively inferior results than EtOH (Table 1, entry 3). The use of ethylene glycol and glycerol as the grinding matrix showed improved yields with the monobromo product **2a** formed in 81% and 85% yield, respectively within 10 min (Table 1, entries 4 and 5). Next, liquid polyethylene glycol, PEG-400 was selected as the LAG agent keeping all other parameters the same. Interestingly, the monobrominated product **2a** was obtained almost exclusively in an excellent yield (91%) within just 5 min of grinding (Table 1, entry 6). The attempted model reaction under solid-state grinding using silica gel was sluggish and it afforded **2a** and **3a** in a 3:2 ratio in lower yields (Table 1, entry 7). Under PEG-400-assisted grinding conditions, a study was conducted to determine the suitable stoichiometry of NBS for the bromination reaction. The study revealed that the increased or decreased stoichiometry of NBS adversely affects the reaction outcome leading to either incomplete conversion (Table 1, entries 8 and 9) or to a low yield of the desired monobromo product due to over-bromination (Table 1, entry 10). On the other hand, increasing the grinding speed (120 rpm) did not increase the yield of the desired product or expedite the reaction (Table 1, entry 11). Whereas upon lowering the grinding speed (70 rpm), the yield of the desired monobromo product decreased marginally, and the reaction took a long time for complete conversion (Table 1, entry 12). Next, a short study was conducted by carrying out the reactions on the model substrate **1a** in the solution phase to understand the advantage of grinding over conventional ways (Table S1 in Supporting Information File 1). Again, PEG-400 was found suitable as the solvent (1–2 mL per mmol of *p*-cresol) for the monobromination of *p*-cresol, but the reactions took several hours for completion and showed inferior chemoselectivity producing dibrominated product **3a** in higher quantity in the solution phase (Table S1, entries 6 and 7 in Supporting Information File 1). However, a thick immiscible mixture was formed when only 0.2 mL of PEG-400 were used and the reaction could not proceed to completion (Table S1, entry 8 in Supporting Information File 1). Further, during aqueous work-up at least 10% loss of the water-soluble crude product **2a** was observed in the conventional solution-phase reactions leading to a drop in the isolated yields. Based on the above observations, the optimal reaction conditions for the electrophilic monobromination was set as to grind the substrates (1 mmol) in an automated grinder with 1.1 mmol of NBS at 100 rpm in PEG-400 (0.2 mL per mmol of the substrate) as a grinding auxiliary.

**Table 1:** Optimization of the reaction conditions for the bromination with NBS.^a^

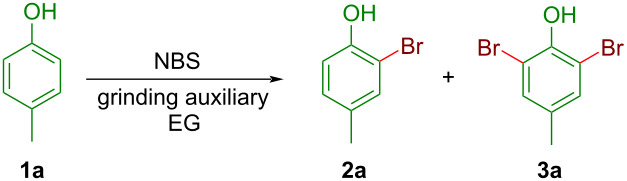						

Entry	Grinding media^b^	Equiv of NBS	Grinding speed (rpm)	Time (min)	Yield^c^ (%)	

					**2a**	**3a**

1	NG	1.1	100	10	57^d^	20
2	EtOH	1.1	100	10	77^d^	12
3	H_2_O	1.1	100	10	63^d^	15
4	ethylene glycol	1.1	100	10	81^d^	06
5	glycerol	1.1	100	10	85	05
6	PEG-400	1.1	100	5	91	03
7	SiO_2_	1.1	100	30	45^d^	28
8	PEG-400	0.9	100	10	62^d^	–
9	PEG-400	1.0	100	5	84^d^	03
10	PEG-400	1.2	100	5	78^d^	08
11	PEG-400	1.1	120	5	89	04
12	PEG-400	1.1	70	15	86	03

^a^1 mmol of **1a** and 1.1 mmol NBS are taken for EG; ^b^0.2 mL of solvent (300 mg for SiO_2_) per mmol of **1a** was used for LAG; ^c^isolated yields; ^d^some amount of starting phenol **1a** was also isolated. NG: neat grinding.

**Scheme 3:**
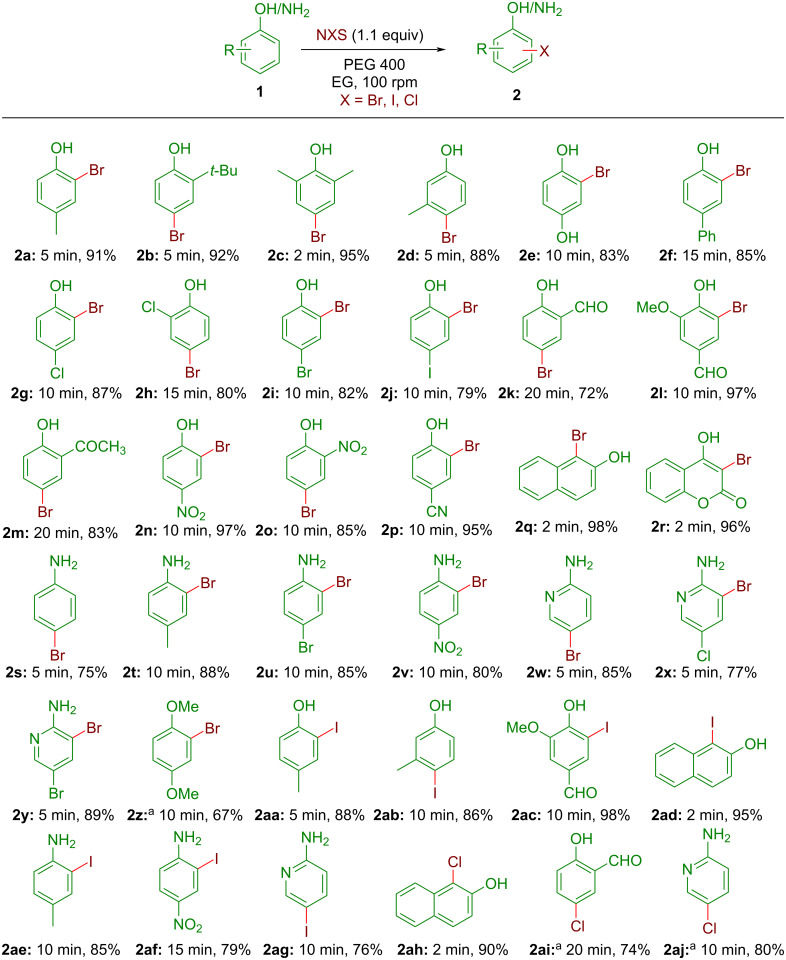
Monohalogenation of phenols and anilines by automated grinding with NXS. All yields refer to the isolated products. Note a: Reactions were carried out in the presence of 10 mol % of conc. H_2_SO_4_.

We next explored the substrate scope of the catalyst-free monobromination under the optimized reaction conditions to validate the effectiveness of our method using an indigenous electrical grinder. The results are summarized in Scheme 3. At the outset, several electron-rich and electron-deficient phenol derivatives were converted to the corresponding monobrominated products **2a**–**r** in high to excellent yields upon employing 1.1 equiv of NBS as the brominating agent and PEG-400 (0.2 mL per mmol of phenols) as the LAG agent. As mentioned, PEG-400 allows the formation of a free-flowing liquid mixture and the reactions were mostly completed within 2–15 min of grinding in an Agate mortar–pestle. In each case, once the reaction got over, the crude product was directly slurried by the addition of silica gel (230–400 mesh, approximately 1 g) and purified by flash chromatography eluting with varying proportions of EtOAc/petroleum ether; thus, a typical work-up step was avoided. Moreover, up to 95% of the side product succinimide was also isolated, and considering its possible conversion to NBS [44] it is an attribute to this green protocol by lowering the E-factor. The products were well-characterized by ^1^H NMR, ^13^C NMR, IR, and CHN analysis. The NMR spectra of the synthesized compounds matched well with the reported data indicating their successful formation. As such not much substituent effect was seen and the protocol worked well for phenols having electron-donating groups (EDG, products **2b–e**, Scheme 3) or even strong electron-withdrawing groups (EWG, products **2n–p**, Scheme 3) affording high yields of the corresponding monobrominated products. The reaction of halogen-substituted phenols also showed higher yields with no exchange of halogen atoms during the course of the reaction (products **2g–j**, Scheme 3). As expected, exclusive *ortho*-bromination to the phenolic hydroxy group was observed for 4-phenylphenol indicating this electrophilic halogenation is selective to electron-rich aromatic rings only (product **2f**, Scheme 3). Also, aromatic halogenation prevails over α-halogenation of a ketomethyl group as was demonstrated by the formation of product **2m** as the sole product for the bromination of 2'-hydroxyacetophenone (product **2m**, Scheme 3). Notably, easily oxidizable groups like –CHO remained unaffected under the reaction conditions (product **2k** and **2l**, Scheme 3). It is worthy to mention that the bromination on 2-naphthol and coumarin was extremely fast affording >95% yields within just 2 min of grinding (products **2q** and **2r**, Scheme 3). Next, a series of aniline derivatives were taken as the substrates for this electrophilic bromination by NBS. To our delight, the corresponding bromo derivatives were formed in high yields (75–89%) within 5–15 min of grinding (products **2s–y**, Scheme 3). Once again, no prominent substituent effect was observed in terms of yields or reaction time. Next, we focused our attention on expanding the substrate scope to other electron-rich aromatic systems. The bromination of hydroquinone dimethyl ether was sluggish and a moderate yield (67%) of the desired monobromo derivative **2z** was achieved only after 30 min of rigorous grinding. However, a negligible conversion was observed for *p*-xylene or mesitylene even after grinding for an hour. Therefore, we restricted our study to the halogenation of phenols and anilines only. Subsequently, a short series of monoiodo derivatives was successfully prepared in high to excellent yields from phenols and anilines by adding 1.1 equiv of NIS with PEG-400 as the LAG agent (product **2aa–ag**, Scheme 3). Notably, both Br- and I-substituents are mainly used as the substrates for cross-coupling reactions indicating the usefulness of the current protocol for quick access to these halo derivatives. Encouraged by this, we attempted monochlorination with selected phenols and anilines. The first attempt with 2-naphthol afforded the desired chloro derivative **2ah** in high yield within 2 min. However, unlike in the case of NBS and NIS, the chlorination by NCS was often found sluggish and complete conversion was not observed even after vigorous grinding for 30 min. Nonetheless, the addition of a catalytic amount of H_2_SO_4_ (10 mol %) was sufficient to activate NCS and the corresponding chloro derivatives were obtained in good yields (product **2ai** and **2aj**, Scheme 3). It is worthy to note that PEG-400 as the grinding auxiliary not only expedited the reaction but also played a key role in availing better regioselectivity. A very high *para*-selectivity was observed for both phenols and aniline substrates with free *o-* and *p*-positions in the case of bromination as well as iodination (product **2b**, **2d**, **2h**, **2k**, **2s**, **2w**, **2ab**, **2ag**, etc. in Scheme 3). In some cases, the formation of negligible amounts of dihalo derivatives (3–5%) could not be avoided. Only for the attempted monobromination of unsubstituted phenol, the addition of 1.1 equiv of NBS afforded a mixture of products with reduced regioselectivity to the expected *p*-bromophenol (yield: 62%). From the mechanistic point of view, it is expected that a standard electrophilic aromatic substitution pathway was followed for the halogenation using NXS (X = Br, I, or Cl). Presumably, PEG-400 with several -O- and terminal -OH functionalities helps to enhance the polarization of the N–X bond. Thus, the formation of the halonium ion (X^+^) in the reaction medium is faster and stabilized by solvation to offer an extra bit of time for the attack of phenol (or aniline) preferably through *p*-position leading to the formation of the thermodynamically stable halo derivative via a σ-complex formation. The high concentration of substrates and reagents in the close proximity in this solvent-less process and grinding force could be the other reasons for the fast reaction kinetics.

**Scheme 4:**
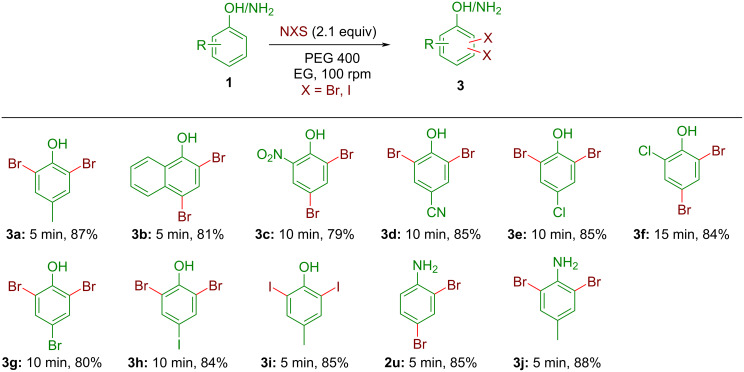
Dihalogenation of phenols and anilines with NXS by automated grinding. All yields refer to the isolated products.

The initial optimization study showed that the presence of excess NBS could increase the yield of undesired dibromo products (Table 1, entry 10). Encouraged by this, a short series of dihalogenated derivatives was prepared under optimized grinding conditions by just changing the stoichiometry of NXS from 1 equiv to 2 equiv (X = Br, I) (Scheme 4). Several electron-rich (products **3a** and **3k**, Scheme 4) and electron-deficient (product **3c**–**h**, Scheme 4) phenols and anilines were successfully converted to the corresponding dibromo derivatives in good to excellent yields within 5–15 min when 2.1 equiv of NBS were used (Scheme 4). The formation of the corresponding monobromo products was not observed. Similarly, *p*-cresol (**1a**) was converted to the corresponding diiodo derivative **3i** in high yield by using 2.1 equiv of NIS indicating the generality of this synthetic protocol. Next, the attempted dihalogenation of aniline derivatives also worked well to afford the desired products in high yields (products **2u** and **3j** in Scheme 4). In all cases, no prominent substituent effect was observed in terms of yields and reaction time.

Next, we planned to further diversify this halogenation protocol via automated grinding for the facile access to trihalogenated derivatives by the use of 3 equiv of *N-*halosuccinimides (X = Br, I) (Table 2). Notably, trihalo phenols and anilines are commercial products and used as intermediates of pharmaceutical and agrochemical products. The basic substrates phenol (entry 1, Table 2) and aniline (entry 5, Table 2) afforded the 2,4,6-tribromo derivatives in the presence of NBS in excellent yields just by grinding for 5 min. Similarly, 3 equiv of NIS ensured the formation of 2,4,6-triiodo derivatives in over 90% yields (entries 8 and 11, Table 2). As known, easy decarboxylation (or desulfonation) was observed for phenols and anilines with carboxylic acid (-CO_2_H) or sulfonic acid (-SO_3_H) groups both at *o*- or *p*-positions leading to the formation of 2,4,6-trihalo phenols and anilines in excellent yields within just 10 min (Table 2) [58]. Thus simple control of the stoichiometry of NXS could offer the versatility in obtaining mono-, di-, or trihalo derivatives as per the requirement within a very short time, which is a sheer advantage of this mechanochemical method. A further study of mechanochemical decarboxylative aromatic halogenations is underway in our laboratory.

**Table 2:** Trihalogenation of phenols and anilines with NXS by automated grinding.^a^

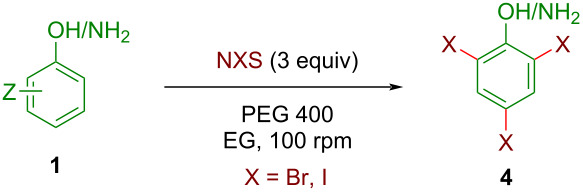				

Entry	Z	Product	Time (min)	Yield (%)

1	H	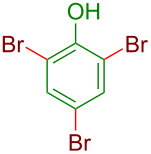 **3g**	05	94
2	*o*-CO_2_H		10	86
3	*p*-CO_2_H		10	89
4	*p*-SO_3_H		10	85

5	H	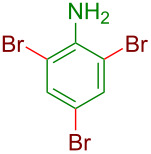 **4a**	05	95
6	*p*-CO_2_H		10	90
7	*p*-SO_3_H		05	92

8	H	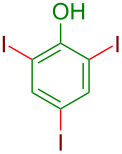 **4b**	05	94
9	*p*-CO_2_H		05	93
10	*p*-SO_3_H		05	95

11	H	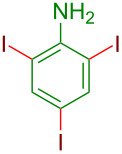 **4c**	05	92
12	*p*-CO_2_H		05	95
13	*p*-SO_3_H		05	97

^a^All yields refer to the isolated products.

**Scheme 5:**
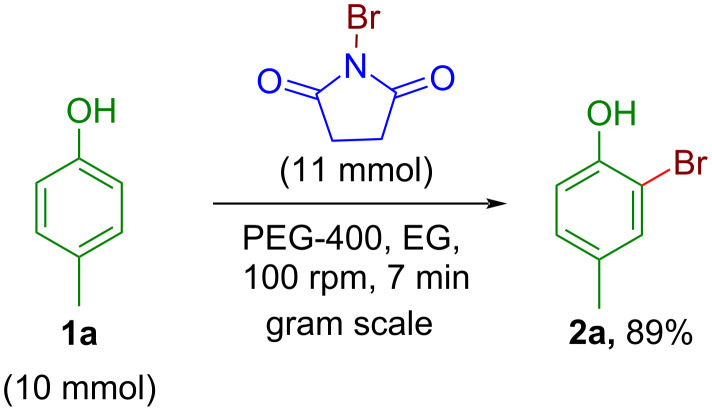
Gram-scale monobromination of *p*-cresol by NBS in the automated grinder.

The scalability of any synthetic protocol is a necessary attribute to access its potential from the laboratory scale to a pilot-scale synthesis. A gram-scale synthesis was conducted with 1.08 g of *p*-cresol (**1a**, 10 mmol) and 1.96 g NBS (11 mmol) in PEG-400 as grinding auxiliary (Scheme 5). The obtained yield of the gram-scale (10 mmol, 89%) synthesis for the monobromo product **2a** was found to be more or less comparable with the yield of small-scale synthesis (1 mmol, 91%). However, the reactions took a slightly longer time (7 min) than the small-scale synthesis. The demonstration of gram-scale reaction implies the potential application of the new protocol in large-scale synthesis with adequate grinding equipment.

Lastly, a comparative study of available methods for *N-*halosuccinimide-aided electrophilic halogenations with our auto-grinding protocol was conducted (Table S2 in Supporting Information File 1). It suggested that the present green method is comparable or better than several other conventional methods in terms of the reaction time, substrate scope, regioselectivity, etc. Moreover, a low E-factor in the range of 2.1–3.6 ensures that the current method could potentially replace the existing conventional methods for the aromatic halogenation of phenols and anilines. Notably, a cost-comparison of our method and the other mechanochemical method by Ghafuri and co-workers was done to understand that the current method is approximately 6 times more cost-effective; besides, the time required for the synthesis of the solid acid catalyst and the cost of high-end milling instruments are additional considerations for that method [52].

## Conclusion

In conclusion, we have developed a facile and sustainable mechanochemical route for the catalyst-free halogenation of phenol and aniline derivatives using *N*-halosuccinimides as the reagent. In the protocol, PEG-400 was used as an LAG agent and the reactions were conducted in an automated grinder in open-air at room temperature for quick access to halogenated derivatives. A wide range of substrates was compatible with NXS (X = Br, I, Cl) for electrophilic aryl halogenation without much substituent effect and by just controlling the stoichiometry of NXS a series of mono-, di-, and trihalogenated phenols and anilines were obtained in a chemoselective manner in good to excellent yields within 2–15 min of grinding. Spontaneous decarboxylation (or desulfonation) was observed in the case of salicylic acids or anthranilic acids leading to 2,4,6-trihalo derivatives when 3 equiv of NXS were used. PEG-400 plays a key role for faster reaction kinetics and to afford better regioselectivity. Almost exclusive *p*-selectivity was observed for the aromatic substrates with free *ortho-* and *para*-positions. The gram-scale reaction shows similar efficiency like smaller batches indicating easy scale-up of this protocol. The method is environmentally friendly and cost-effective having key attributes like simple instrumentation, no aqueous workup, short reaction time, and mild reaction conditions.

## Experimental

### General procedure for monohalogenation of phenols and anilines

The phenol derivative (**1**, 1.0 mmol) was taken in an Agate mortar attached to an electrical grinder, PEG-400 (0.2 mL) was added as LAG agent, and to the mixture, NBS (1.1 mmol) was added in several portions over 5 min under continuous grinding by a pestle at 100 rpm. The electrical grinding was continued for the specific time period (as mentioned in Scheme 3) and the completion of the reaction was monitored by checking TLC after 2 min, 5 min, 10 min, 15 min as applicable for the reaction. After complete conversion was observed, 0.8–1 g of silica gel (230–400 mesh) was added and the slurry was subjected to flash chromatography and eluted with a mixture of EtOAc/petroleum ether to afford the pure monobromo phenol derivative. The side product succinimide was subsequently eluted using MeOH/CHCl_3_ 1:10

## Supporting Information

File 1 Experimental procedures, spectral data, tables, and copies of spectra.
