# Supplementary material for: Automated grindstone chemistry: a simple and facile way for PEG-assisted stoichiometry-controlled halogenation of phenols and anilines using N-halosuccinimides
Source: Beilstein J Org Chem. 2022 Aug 9;18:999–1008. doi: 10.3762/bjoc.18.100 (PMC9379637; doi:10.3762/bjoc.18.100)
Supplement: File 1 — Experimental procedures, spectral data, tables, and copies of spectra. [file Beilstein_J_Org_Chem-18-999-s001.pdf]

BEILSTEIN JOURNAL OF ORGANIC CHEMISTRY

Supporting Information

for

Automated grindstone chemistry: a simple and facile way for PEG-assisted stoichiometry-controlled halogenation of phenols and anilines using *N*-halosuccinimides

Dharmendra Das, Akhil A. Bhosle, Amrita Chatterjee and Mainak Banerjee

Beilstein J. Org. Chem. **2022,** *18,* 999–1008. doi:10.3762/bjoc.18.100

Experimental procedures, spectral data, tables, and copies of spectra

License and Terms: This is a supporting information file under the terms of the Creative Commons Attribution License (https://creativecommons.org/licenses/by/4.0). Please note that the reuse, redistribution and reproduction in particular requires that the author(s) and source are credited and that individual graphics may be subject to special legal provisions.
The license is subject to the *Beilstein Journal of Organic Chemistry* terms and conditions: (https://www.beilstein-journals.org/bjoc/terms)

Table of contents

Experimental procedures S2
Characterization data of products S3–S12
Solution phase study S13
Comparative table S14
^{1}H NMR and ^{13}C NMR spectra of products S15–S63
References S64–S66

Experimental procedures

General information

All the reagents and solvents of AR grade were procured from commercial sources and used without further purification. The mechanochemical grinding reactions were carried out in an indigenous electrical grinder (Scientech instruments, India) with Agate-made mortar and pestle. The thin-layer chromatography (TLC) of 0.25 mm silica gel aluminum plates (60F-254) were used to monitor the progress of the reaction, and visualization was done using UV light (254 or 365 nm). The synthesized products were purified by flash chromatography or conventional column chromatography with 100-200 mesh silica gel. ^{1}H NMR and ^{13}C NMR spectra were recorded on Bruker AVANCE (400 MHz and 500 MHz) instrument and tetramethylsilane as the internal standard. The multiplicity of NMR peaks was represented using standard abbreviations, and chemical shifts are reported in parts per million (δ) units. CHN data were recorded with the Vario MICRO elementar CHNS analyzer. IR spectra were recorded in KBr pellets with IR Affinity 1, Shimadzu.

General procedure for di-halogenation of the substrates

The phenol (or aniline) derivative (1.0 mmol) was taken in an Agate mortar attached to an electrical grinder and to it NXS (2.1 mmol; X = Br, I) and PEG-400 (0.2 mL) were added one after another, and grinding was continued by an electrically operated pestle for the specific time period as mentioned in Table 3. The completion of the reaction was monitored by TLC. After complete conversion, 1 g of silica gel (230-400 mesh) was added and the slurry was subjected to flash chromatography and eluted with a mixture of EtOAc-petroleum ether to afford the pure di-bromo phenol (or aniline) derivative. Once again, the side product succinimide was subsequently eluted using 1:10 MeOH-$CHCl_3$.

General procedure for tri-halogenation of the substrates

Phenol or aniline derivatives (**1**, 1.0 mmol), NXS (3.0 mmol; X = Br, I) were taken in an agate mortar containing PEG-400 (0.2 mL) and electrical grinding was continued with a pestle at 100 rpm for the specific time period as mentioned for respective substrates in Table 4. 1 g of silica gel (230-400 mesh) was added and the slurry was subjected to flash chromatography and eluted with a mixture of EtOAc-petroleum ether to afford the pure tri-bromo phenol (or aniline) derivative.

Characterization data of products 2a–aj, 3a–j, and 4a–c

2-Bromo-4-methylphenol (2a) [1]: Off-white solid, 170 mg (91%), m.p. 57-59 °C (lit. m.p. 55-57 °C); ^{1}H NMR (400 MHz, $CDCl_3$): δ (ppm) 2.27 (s, 3H), 5.36 (s, 1H), 6.91 (d, J = 8.2 Hz, 1H), 7.01 (dd, J_1 = 1.4 Hz, J_2 = 8.2 Hz, 1H), 7.271-7.274 (m, 1H). ^{13}C NMR (100 MHz, $CDCl_3$): δ (ppm) 20.2, 109.8, 115.7, 129.8, 131.4, 132.1, 150.0. Anal. Calcd for C_7H_7BrO: C, 44.95; H, 3.77. Found: C, 45.01; H, 3.76. IR (KBr) $\tilde{v}$: 3498, 2924, 1608, 1493, 1040, 866, 812, 760, 671 and 550 cm^{-1}.

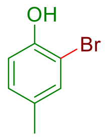

2a

4-Bromo-2-*tert*-butylphenol (2b) [2]: Light-grey liquid, 210 mg (92%), ^{1}H NMR (400 MHz, $CDCl_3$): δ (ppm) 1.39 (s, 9H), 4.14 (br, 1H), 6.56 (d, J = 8.4 Hz, 1H), 7.16 (dd, J_1 = 2.4 Hz, J_2 = 8.4 Hz, 1H), 7.35 (d, J = 2.4 Hz, 1H). ^{13}C NMR (100 MHz, $CDCl_3$): δ (ppm) 29.3, 34.7, 112.8, 118.1, 129.5, 130.1, 138.5, 153.3. Anal. Calcd for $C_{10}H_{13}BrO$: C, 52.42; H, 5.72. Found: C, 52.51; H, 5.74. IR (KBr) $\tilde{v}$: 3549, 2959, 1597, 1489, 1398, 1249, 1176, 1082, 880, 806, 706, 631 and 567 cm^{-1}.

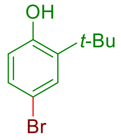

2b

4-Bromo-2,6-dimethylphenol (2c) [1]: Light-brown solid, 190 mg (95%), m.p. 79-80 °C (lit. m.p. 74-78 °C); ^{1}H NMR (400 MHz, $CDCl_3$): δ (ppm) 2.22 (s, 3H), 4.58 (s, 1H), 7.10 (s, 2H). ^{13}C NMR (100 MHz, $CDCl_3$): δ (ppm) 15.7, 112.0, 125.2, 131.0, 151.3. Anal. Calcd for C_8H_9BrO: C, 47.79; H, 4.51. Found: C, 47.65; H, 4.49. IR (KBr) $\tilde{v}$: 3383, 2916, 1609, 1476, 1329, 1190, 941, 853, 718 and 555 cm^{-1}.

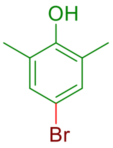

2c

4-Bromo-3-methylphenol (2d) [1,2]: Off-white solid, 164 mg (88%), m.p. 59-60 °C (lit. m.p. 59-61 °C); ^{1}H NMR (400 MHz, $CDCl_3$): δ (ppm) 2.34 (s, 3H), 4.97 (s, 1H), 6.55 (dd, J_1 = 3.0 Hz, J_2 = 8.8 Hz, 1H), 6.73 (d, J = 3.0 Hz, 1H), 7.35 (d, J = 8.4 Hz, 1H). ^{13}C NMR (100 MHz, $CDCl_3$): δ (ppm) 22.9, 114.5, 115.5, 117.8, 133.0, 139.2, 154.6. Anal. Calcd for C_7H_7BrO: C, 44.95; H, 3.77. Found: C, 45.05; H, 3.73. IR (KBr) $\tilde{v}$: 3383, 2922, 1587, 1169, 1028, 860, 810 and 602 cm^{-1}.

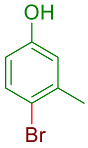

2d

2-Bromobenzene-1,4-diol (2e) [3,4]: Grey solid, 156 mg (83%), m.p. 114-116 °C (lit. m.p. 114 °C); ^{1}H NMR (400 MHz, $CDCl_3$): δ (ppm) 6.73 (dd, J_1 = 3.2 Hz, J_2 = 8.8 Hz, 1H), 6.89 (d, J = 8.8 Hz, 1H), 6.99 (d, J = 3.0 Hz, 1H). ^{13}C NMR (100 MHz, $CDCl_3$): δ (ppm) 109.9, 115.8, 116.3, 118.6, 147.2, 149.5. Anal. Calcd for $C_6H_5BrO_2$: C, 38.13; H, 2.67. Found: C, 38.31; H, 2.69. IR (KBr) $\tilde{v}$: 3487, 3372, 1624, 1585, 1312, 1263, 1028, 893, 746, 638 and 548 cm^{-1}.

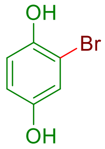

2e

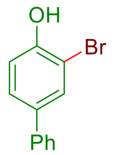

2f

3-Bromo-1,1’-biphenyl-4-ol (2f) [5]: White crystalline solid, 210 mg (85%), m.p. 93-95 °C (lit. m.p. 94-95 °C); ^{1}H NMR (400 MHz, $CDCl_3$): δ (ppm) 5.55 (br, 1H), 7.10 (d, J = 8.4 Hz, 1H), 7.31-7.36 (m, 1H), 7.40-7.48 (m, 3H), 7.50-7.53 (m, 2H), 7.71 (d, J = 2.4 Hz, 1H). ^{13}C NMR (100 MHz, $CDCl_3$): δ (ppm) 110.6, 116.3, 126.7, 127.2, 127.9, 128.8, 130.4, 135.4, 139.4, 151.6. Anal. Calcd for $C_{12}H_9BrO$: C, 57.86; H, 3.64. Found: C, 58.01; H, 3.63. IR (KBr) $\tilde{\nu}$: 3300, 2361, 1603, 1487, 1229, 1042, 882, 762, 671 and 581 cm^{-1}.

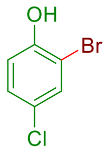

2g

2-Bromo-4-chlorophenol (2g) [1]: Yellow oil, 180 mg (87%); ^{1}H NMR (400 MHz, $CDCl_3$): δ (ppm) 5.56 (s, 1H), 6.95 (d, J = 8.8 Hz, 1H), 7.19 (dd, J_1 = 2.4 Hz, J_2 = 8.8 Hz, 1H), 7.46 (s, 1H). ^{13}C NMR (100 MHz, $CDCl_3$): δ (ppm) 110.3, 116.9, 125.8, 129.8, 131.3, 151.2. Anal. Calcd for C_6H_4BrClO: C, 34.74; H, 1.94. Found: C, 34.67; H, 1.93. IR (KBr) $\tilde{\nu}$: 3501, 3070, 1703, 1578, 1275, 1180, 862, 698, and 552 cm^{-1}.

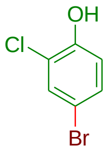

2h

4-Bromo-2-chlorophenol (2h) [1]: Peach crystalline, 162 mg (80%); ^{1}H NMR (400 MHz, $CDCl_3$): δ (ppm) 5.54 (s, 1H), 6.90 (d, J = 8.6 Hz, 1H), 7.29 (dd, J_1 = 2.4 Hz, J_2 = 8.4 Hz, 1H), 7.46 (d, J = 2.4 Hz, 1H). ^{13}C NMR (100 MHz, $CDCl_3$): δ (ppm) 112.3, 117.6, 120.8, 131.3, 131.4, 150.7. Anal. Calcd for C_6H_4BrClO: C, 34.74; H, 1.94. Found: C, 34.70; H, 1.97. IR (KBr) $\tilde{\nu}$: 3520, 3080, 1580, 1327, 1186, 862, 709, and 550 cm^{-1}.

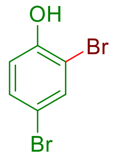

2i

2,4-Dibromophenol (2i) [1]: White solid, 206 mg (82%), m.p. 37-38 °C (lit. m.p. 37-39 °C); ^{1}H NMR (400 MHz, $CDCl_3$): δ (ppm) 5.61 (br, 1H), 6.90 (d, J = 8.6 Hz, 1H), 7.32 (dd, J_1 = 2.0 Hz, J_2 = 8.4 Hz, 1H), 7.59 (d, J = 2.2 Hz, 1H). ^{13}C NMR (100 MHz, $CDCl_3$): δ (ppm) 110.8, 112.6, 117.4, 132.1, 134.1, 151.7. Anal. Calcd for $C_6H_4Br_2O$: C, 28.61; H, 1.60. Found: C, 28.67; H, 1.57. IR (KBr) $\tilde{\nu}$: 3406, 2984, 1699, 1277, 867, 743, 683, and 546 cm^{-1}.

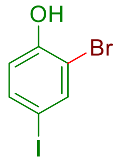

2j

2-Bromo-4-iodophenol (2j) [6]: Light-brown solid, 235 mg (79%), m.p. 50-53 °C (lit. m.p. 52-54 °C); ^{1}H NMR (400 MHz, $CDCl_3$): δ (ppm) 5.59 (br, 1H), 6.78 (d, J = 8.6 Hz, 1H), 7.49 (dd, J_1 = 2.0 Hz, J_2 = 8.4 Hz, 1H), 7.75 (d, J = 2.0 Hz, 1H). ^{13}C NMR (100 MHz, $CDCl_3$): δ (ppm) 82.0, 111.3, 118.1, 138.0, 139.6, 152.3. Anal. Calcd for C_6H_4BrIO: C, 24.11; H, 1.35. Found: C, 24.21; H, 1.36. IR (KBr) $\tilde{\nu}$: 3499, 1709, 1395, 1182, 868, 744, 677, 608 and 542 cm^{-1}.

5-Bromo-2-hydroxybenzaldehyde (2k) [7]: Beige solid, 144 mg (72%), m.p. 102-104 °C (lit. m.p. 104-105 °C); ^{1}H NMR (500 MHz, $CDCl_3$): δ (ppm) 6.90 (d, J = 8.8 Hz, 1H), 7.59 (dd, J_1 = 2.8 Hz, J_2 = 8.8 Hz, 1H), 7.66 (d, J = 2.6 Hz, 1H), 9.83 (s, 1H), 10.92 (s, 1H). ^{13}C NMR (125 MHz, $CDCl_3$): δ (ppm) 111.3, 119.7, 121.7, 135.6, 139.7, 160.5, 195.4. Anal. Calcd for $C_7H_5BrO_2$: C, 41.83; H, 2.51. Found: C, 41.75; H, 2.47. IR (KBr) $\tilde{v}$: 3229, 2923, 2866, 1677, 1472, 1169, 825, 692 cm^{-1}.

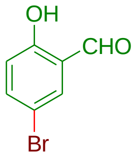

2k

3-Bromo-4-hydroxy-5-methoxybenzaldehyde (2l) [2]: White solid, 224 mg (97%), m.p. 164-166 °C (lit. m.p. 166-168 °C); ^{1}H NMR (400 MHz, $CDCl_3$): δ (ppm) 3.98 (s, 3H), 6.54 (br, 1H), 7.36 (d, J = 1.6 Hz, 1H), 7.64 (d, J = 1.6 Hz, 1H), 9.78 (s, 1H). ^{13}C NMR (100 MHz, $CDCl_3$): δ (ppm) 56.6, 108.0, 108.1, 130.0, 130.1, 147.7, 148.9, 189.7. Anal. Calcd for $C_8H_7BrO_3$: C, 41.59; H, 3.05. Found: C, 41.70; H, 3.06. IR (KBr) $\tilde{v}$: 3298, 2941, 2848, 2745, 1674, 1591, 1159, 1047, 855, 831, 795, 681cm^{-1}.

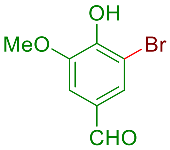

2l

5-Bromo-2-hdroxyacetophenone (2m) [7]: Off-white solid, 179 mg (83%), m.p. 58-60 °C (lit. m.p. 59-60 °C); ^{1}H NMR (500 MHz, $CDCl_3$): δ (ppm) 2.61 (s, 3H), 6.87 (d, J = 8.5 Hz, 1H), 7.53 (dd, J_1 = 2.5 Hz, J_2 = 8.6 Hz, 1H), 7.82 (d, J = 2.5 Hz, 1H), 12.14 (s, 1H). ^{13}C NMR (125 MHz, $CDCl_3$): δ (ppm) 26.7, 110.4, 120.4, 120.8, 132.9, 139.1, 161.2, 203.5. Anal. Calcd for $C_8H_7BrO_2$: C, 44.68; H, 3.28. Found: C, 44.51; H, 3.19. IR (KBr) $\tilde{v}$: 3014, 1753, 1643, 1457, 1194, 802, 730, 615 cm^{-1}.

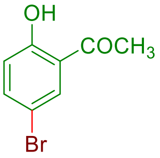

2m

2-Bromo-4-nitrophenol (2n) [1]: Off-white solid, 210 mg (97%), m.p. 110-112 °C (lit. m.p. 111-114 °C); ^{1}H NMR (400 MHz, $CDCl_3$): δ (ppm) 6.82 (d, J = 9.2 Hz, 1H), 8.0 (d, J = 9.2 Hz, 1H), 8.26 (s, 1H). ^{13}C NMR (100 MHz, $CDCl_3$): δ (ppm) 109.7, 114.5, 124.6, 126.6, 139.0, 156.1. Anal. Calcd for $C_6H_4BrNO_3$: C, 33.06; H, 1.85; N, 6.43. Found: C, 33.16; H, 1.87; N, 6.38. IR (KBr) $\tilde{v}$: 3375, 3078, 1514, 1327, and 633 cm^{-1}.

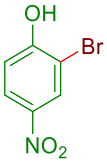

2n

4-Bromo-2-nitrophenol (2o) [8]: White solid, 185 mg (85%), m.p. 90-92 °C (lit. m.p. 89-92 °C); ^{1}H NMR (500 MHz, $CDCl_3$): δ (ppm) 7.10 (d, J = 8.9 Hz, 1H), 7.69 (dd, J_2 = 2.5 Hz, J_1 = 9.0 Hz, 1H), 8.27 (d, J = 2.5 Hz, 1H), 10.60 (s, 1H). ^{13}C NMR (125 MHz, $CDCl_3$): δ (ppm) 111.7, 121.7, 127.3, 134.0, 140.3, 154.1. Anal. Calcd for $C_6H_4BrNO_3$: C, 33.06; H, 1.85; N, 6.43. Found: C, 33.13; H, 1.83; N, 6.39. IR (KBr) $\tilde{v}$: 3249, 3078, 1536, 1327, and 519 cm^{-1}.

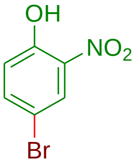

2o

3-Bromo-4-hydroxybenzonitrile (2p) [9]: White solid, 188 mg (95%), m.p. 155-157 °C (lit. m.p. 156 °C); ^{1}H NMR (500 MHz, $CDCl_3$): δ (ppm) 6.60 (s, 1H,) 7.08 (d, *J* = 8.4 Hz, 1H), 7.52 (dd, J_1 = 2.0 Hz, J_2 = 8.4 Hz, 1H), 7.79 (d, *J* = 2.0 Hz, 1H). ^{13}C NMR (125 MHz, $CDCl_3$): δ (ppm) 105.0. 110.6, 116.9, 117.6, 133.3, 136.2, 156.6. Anal. Calcd for C_7H_4BrNO: C, 42.46; H, 2.04; N, 7.07. Found: C, 42.39; H, 2.01; N, 6.33. IR (KBr) $\tilde{v}$: 3438, 3089, 2228, 1661, 1428, 1121, 638 and 575 cm^{-1}.

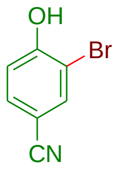

2p

1-Bromonaphthalen-2-ol (2q) [1,2]: Off-white solid, 219 mg (98%), m.p. 77-79 °C (lit. m.p. 78-81 °C); ^{1}H NMR (400 MHz, $CDCl_3$): δ (ppm) 5.97 (s, 1H), 7.29–7.31 (m, 1H), 7.41–7.45 (m, 1H), 7.58–7.62 (m, 1H), 7.76–7.82 (m, 2H), 8.07 (d, *J* = 8.8 Hz, 1H). ^{13}C NMR (100 MHz, $CDCl_3$): δ (ppm) 106.1, 117.1, 124.1, 125.3, 127.8, 128.2, 129.3, 129.7, 132.3, 150.6. Anal. Calcd for $C_{10}H_7BrO$: C, 53.84; H, 3.16. Found: C, 54.02; H, 3.17. IR (KBr) $\tilde{v}$: 3279, 3055, 1499, 808, 644 and 519 cm^{-1}.

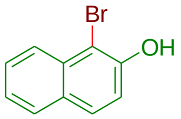

2q

3-Bromo-4-hydroxycoumarin (2r) [6,10]: White solid, 232 mg (96%), m.p. 189-191 °C (lit. m.p. 192-194 °C); ^{1}H NMR (400 MHz, $CDCl_3$): δ (ppm) 7.36–7.42 (m, 2H), 7.64–7.69 (m, 1H), 7.95 (dd, J_1 = 1.6 Hz, J_2 = 8.0 Hz, 1H), 9.18 (br, 1H). ^{13}C NMR (100 MHz, $CDCl_3$): δ (ppm) 89.1, 115.9, 116.4, 123.4, 124.4, 132.8, 151.7, 158.5, 162.3. Anal. Calcd for $C_9H_5BrO_3$: C, 44.85; H, 2.09. Found: C, 44.94; H, 2.13. IR (KBr) $\tilde{v}$: 3184, 2344, 1699, 1551, 1211, 826, 746 and 590 cm^{-1}.

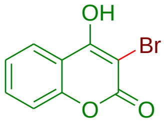

2r

4-Bromoaniline (2s) [11,12]: Brown solid, 130 mg (75%), m.p. 62-63 °C (lit. m.p. 63-64 °C); ^{1}H NMR (400 MHz, $CDCl_3$): δ (ppm) 3.51 (br, 2H), 6.56 (d, *J* = 8.8 Hz, 2H), 7.23 (d, *J* = 8.8 Hz, 2H). ^{13}C NMR (100 MHz, $CDCl_3$): δ (ppm) 110.2, 116.7, 132.0, 145.3. Anal. Calcd for C_6H_6BrN: C, 41.89; H, 3.52; N, 8.14. Found: C, 41.78; H, 3.50; N, 8.09. IR (KBr) $\tilde{v}$: 3474, 3381, 1612, 1489, 1287, 1180, 1070, 1005, 818, 692 and 604 cm^{-1}.

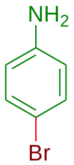

2s

2-bromo-4-methylaniline (2t) [12]: Brown oil, 164 mg (88%); ^{1}H NMR (500 MHz, $CDCl_3$): δ (ppm) 2.23 (s, 3H), 3.83 (br, 2H), 6.73 (d, *J* = 7.5 Hz, 1H), 6.97 (d, *J* = 7.5 Hz, 1H), 7.29 (s, 1H). ^{13}C NMR (125 MHz, $CDCl_3$): δ (ppm) 20.0, 109.2, 115.7, 128.9, 130.1, 132.7, 141.5. Anal. Calcd for C_7H_8BrN: C, 45.19; H, 4.33; N, 7.53. Found: C, 45.22; H, 4.30; N, 7.61. IR (KBr) $\tilde{v}$: 3477, 3371, 3018, 1624, 1489, 1309, 746, 1038 and 810 cm^{-1}.

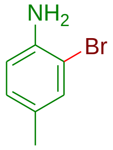

2t

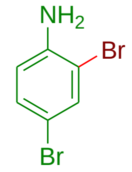

2u

2,4-Dibromoaniline (2u) [13]: Brown solid, 213 mg (85%), m.p. 78-80 °C (lit. m.p. 79-80 °C); ^{1}H NMR (400 MHz, CDCl$_3$): δ (ppm) 4.08 (br, 2H), 6.64 (d, J = 8.4 Hz, 1H), 7.19 (dd, J_1 = 2.4 Hz, J_2 = 8.8 Hz, 1H), 7.53 (d, J = 2.4 Hz, 1H). ^{13}C NMR (100 MHz, CDCl$_3$): δ (ppm) 108.8, 109.5, 116.7, 131.1, 134.4, 143.2. Anal. Calcd for $C_6H_5Br_2N$: C, 28.72; H, 2.01; N, 5.58. Found: C, 28.69; H, 2.02; N, 5.56. IR (KBr) $\tilde{\nu}$: 3404, 3302, 3075, 2924, 1618, 1479, 1393, 1288, 1032, 866, 810, 684 and 536 cm^{-1}.

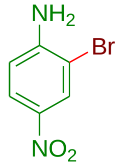

2v

2-Bromo-4-nitroaniline (2v) [14]: Yellow solid, 174 mg (80%), m.p. 88-90 °C (lit. m.p. 89-93 °C); ^{1}H NMR (400 MHz, CDCl$_3$): δ (ppm) 4.86 (br, 2H), 6.74 (d, J = 8.8 Hz, 1H), 8.01 (dd, J_1 = 2.4 Hz, J_2 = 8.8 Hz, 1H), 8.35 (d, J = 2.4 Hz, 1H). ^{13}C NMR (100 MHz, CDCl$_3$): δ (ppm) 106.9, 113.4, 124.9, 129.1, 138.9, 149.9. Anal. Calcd for $C_6H_5BrN_2O_2$: C, 33.21; H, 2.32; N, 12.91. Found: C, 33.29; H, 2.30; N, 12.99. IR (KBr) $\tilde{\nu}$: 3487, 3372, 3098, 1624, 1489, 1312, 746, 698, 638 and 548 cm^{-1}.

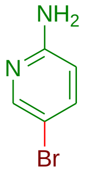

2w

2-Amino-5-bromopyridine (2w) [15,16]: Light brown solid, 148 mg (85%), m.p. 133-135 °C (lit. m.p. 135-138 °C); ^{1}H NMR (400 MHz, CDCl$_3$): δ (ppm) 4.57 (br, 2H), 6.40 (d, J = 8.8 Hz, 1H), 7.48 (dd, J_1 = 2.4 Hz, J_2 = 8.8 Hz, 1H), 8.06 (s, 1H). ^{13}C NMR (100 MHz, CDCl$_3$): δ (ppm) 108.2, 110.0, 140.1, 148.6, 157.0. Anal. Calcd for $C_5H_5BrN_2$: C, 34.71; H, 2.91; N, 16.19. Found: C, 34.60; H, 2.90; N, 16.24. IR (KBr) $\tilde{\nu}$: 3453, 3294, 3152, 1707, 1589, 1549, 1487, 1389, 1261, 1142, 1090, 1001, 927, 825, 633 and 515 cm^{-1}.

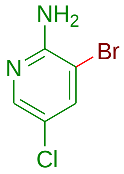

2x

2-Amino-3-bromo-5-chloropyridine (2x) [16]: Pale-yellow solid, 160 mg (77%), m.p. 78-79 °C (lit. m.p. 78-84 °C); ^{1}H NMR (400 MHz, CDCl$_3$): δ (ppm) 5.03 (br, 2H), 6.66 (d, J = 2.2 Hz, 1H), 7.97 (d, J = 2.0 Hz, 1H). ^{13}C NMR (100 MHz, CDCl$_3$): δ (ppm) 104.1, 120.4, 139.6, 145.2, 154.0. Anal. Calcd for $C_5H_4BrClN_2$: C, 28.95; H, 1.94; N, 13.50. Found: C, 28.93; H, 1.93; N, 13.47. IR (KBr) $\tilde{\nu}$: 3474, 3291, 3146, 2345, 1638, 1389, 1242, 1124, 889, 741, 656 and 507 cm^{-1}.

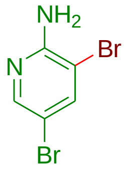

2y

2-Amino-3,5-dibromopyridine (2y) [16,17]: White crystalline solid, 224 mg (89%), m.p. 99-101 °C (lit. m.p. 104-105 °C); ^{1}H NMR (400 MHz, CDCl$_3$): δ (ppm) 4.99 (br, 2H), 7.76 (d, J = 2.4 Hz, 1H), 8.05 (d, J = 2.2 Hz, 1H). ^{13}C NMR (100 MHz, CDCl$_3$): δ (ppm) 104.5, 107.2, 141.9, 147.6, 154.3. Anal. Calcd for $C_5H_4Br_2N_2$: C, 23.84; H, 1.60; N, 11.12. Found: C, 23.80; H, 1.64; N, 11.04. IR (KBr) $\tilde{\nu}$: 3464, 3279, 3140, 2345, 1570, 1385, 1240, 893, 743, 694, and 544 cm^{-1}.

2-Bromo-1,4-dimethoxybenzene (2z) [18]: Light brown liquid, 146 mg (67%); ^{1}H NMR (500 MHz, $CDCl_3$): δ (ppm) 6.97-7.00 (m, 2H), 7.28 (d, J = 2.7 Hz, 1H). ^{13}C NMR (125 MHz, $CDCl_3$): δ (ppm) 55.8, 56.8, 112.8, 113.6, 114.6, 118.9, 150.2, 153.9. Anal. Calcd for $C_8H_9BrO_2$: C, 44.27; H, 4.18. Found: C, 44.21; H, 4.12. IR (KBr) $\tilde{\nu}$: 3030, 2947, 1633, 1509, 1237, 1032, 816, 702, 515 cm^{-1}.

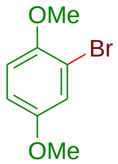

2z

2-Iodo-4-methylphenol (2aa) [19]: Yellow oil, 206 mg (88%); ^{1}H NMR (400 MHz, $CDCl_3$): δ (ppm) 2.25 (s, 3H), 5.16 (br, 1H), 6.88 (d, J = 8.4 Hz, 1H), 7.04 (dd, J_1 = 1.6 Hz, J_2 = 8.4 Hz, 1H), 7.48 (s, 1H). ^{13}C NMR (100 MHz, $CDCl_3$): δ (ppm) 19.9, 85.4, 114.7, 130.8, 131.9, 138.3, 152.6. Anal. Calcd for C_7H_7IO: C, 35.92; H, 3.01. Found: C, 36.01; H, 3.04. IR (KBr) $\tilde{\nu}$: 3482, 2920, 1601, 1485, 1246, 858, 754, 665 and 544 cm^{-1}.

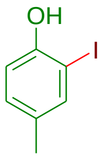

2aa

4-Iodo-3-methylphenol (2ab) [20]: Yellow liquid, 201 mg (86%); ^{1}H NMR (500 MHz, $CDCl_3$): δ (ppm) 2.36 (s, 3H), 6.42 (dd, J_1 = 3.2 Hz, J_2 = 8.5 Hz, 1H), 6.76 (d, J = 3.0 Hz, 1H), 7.60 (d, J = 8.5 Hz, 1H), 7.48 (s, 1H). ^{13}C NMR (125 MHz, $CDCl_3$): δ (ppm) 29.1, 89.6, 114.9, 117.1, 139.5, 142.6, 155.7. Anal. Calcd for C_7H_7IO: C, 35.92; H, 3.01. Found: C, 36.01; H, 3.02. IR (KBr) $\tilde{\nu}$: 3351, 1571, 1465, 1285, 1160, 1010, 807, 682 cm^{-1}.

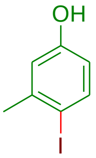

2ab

4-Hydroxy-3-iodo-5-methoxybenzaldehyde (2ac) [2]: Pale-yellow solid, 272 mg (98%), m.p. 179-181 °C (lit. m.p. 180-182 °C); ^{1}H NMR (400 MHz, $CDCl_3$): δ (ppm) 3.97 (s, 3H), 6.71 (br, 1H), 7.38 (d, J = 1.6 Hz, 1H), 7.82 (d, J = 1.6 Hz, 1H), 9.77 (s, 1H). ^{13}C NMR (100 MHz, $CDCl_3$): δ (ppm) 56.5, 80.5, 108.6, 131.1, 136.2, 146.5, 151.4, 189.5. Anal. Calcd for $C_8H_7IO_3$: C, 34.56; H, 2.54. Found: C, 34.77; H, 2.55. IR (KBr) $\tilde{\nu}$: 3156, 2847, 1460, 1416, 1354, 1294, 855, 785, 673 and 584 cm^{-1}.

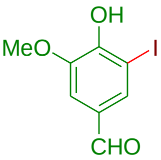

2ac

1-Iodonaphthalen-2-ol (2ad) [21]: Off-white solid, 256 mg (95%), m.p. 94-96 °C (lit. m.p. 93-94 °C); ^{1}H NMR (500 MHz, $CDCl_3$): δ (ppm) 5.82 (s, 1H), 7.25 (d, J = 8.2 Hz, 1H), 7.38 (t, J = 8.2 Hz, 1H), 7.55 (t, J = 8.0 Hz, 1H), 7.73-7.75 (m, 2H), 7.93 (d, J = 8.2 Hz, 1H). ^{13}C NMR (125 MHz, $CDCl_3$): δ (ppm) 86.2, 116.4, 124.1, 128.2, 128.3, 129.6, 130.2, 130.6, 134.8, 153.7. Anal. Calcd for $C_{10}H_7IO$: C, 44.47; H, 2.61. Found: C, 44.31; H, 2.58. IR (KBr) $\tilde{\nu}$: 3363, 1590, 1482, 1213, 999, 813, 593 cm^{-1}.

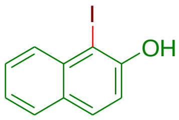

2ad

2-Iodo-4-methylaniline (2ae) [22]: Yellow solid, 198 mg (85%), m.p. 38-40 °C (lit. m.p. 39 °C); ^{1}H NMR (400 MHz, $CDCl_3$): δ (ppm) 2.21 (s, 3H), 3.74 (br, 2H), 6.70 (d, J = 8.0 Hz, 1H), 6.95 (dd, J_1 = 1.4 Hz, J_2 = 8.0 Hz, 1H), 7.48 (d, J = 1.4 Hz, 1H). ^{13}C NMR (100 MHz, $CDCl_3$): δ (ppm) 19.8, 84.3, 114.7, 129.6, 130.0, 139.0, 144.2. Anal. Calcd for C_7H_8IN: C, 36.08; H, 3.46; N, 6.01. Found: C, 36.18; H, 3.43; N, 5.94. IR (KBr) $\tilde{v}$: 3447, 3360, 2920, 1732, 1495, 1155, 810, 665 and 540 cm^{-1}.

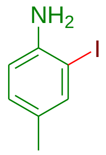

2ae

2-Iodo-4-nitroaniline (2af) [23]: Yellow solid, 209 mg (79%), m.p. 100-102 °C (lit. m.p. 98-107 °C); ^{1}H NMR (400 MHz, $CDCl_3$): δ (ppm) 4.83 (br, 2H), 6.70 (d, J = 8.8 Hz, 1H), 8.05 (dd, J_1 = 2.4 Hz, J_2 = 8.8 Hz, 1H), 8.55 (d, J = 2.6 Hz, 1H). ^{13}C NMR (100 MHz, $CDCl_3$): δ (ppm) 80.5, 112.2, 125.7, 135.5, 139.2, 152.4. Anal. Calcd for $C_6H_5IN_2O_2$: C, 27.30; H, 1.91; N, 10.61. Found: C, 27.34; H, 1.94; N, 10.54. IR (KBr) $\tilde{v}$: 3478, 3372, 2924, 2347, 1609, 1491,1258, 1117, 899, 746, 681 and 638 cm^{-1}.

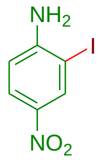

2af

2-Amino-5-iodopyridine (2ag) [24]: Yellow solid, 167 mg (76%), m.p. 128-131 °C (lit. m.p. 128-130 °C); ^{1}H NMR (500 MHz, DMSO-d_6): δ (ppm) 5.90 (s, 2H), 6.45 (t, J = 8.0 Hz, 1H), 7.36 (t, J = 8.2 Hz, 1H), 7.89 (d, J = 8.0 Hz, 1H). ^{13}C NMR (125 MHz, DMSO-d_6): δ (ppm) 72.3, 111.9, 147.4, 152.9, 159.4. Anal. Calcd for $C_5H_5IN_2$: C, 27.30; H, 2.29; N, 12.73. Found: C, 27.21; H, 2.32; N, 12.59. IR (KBr) $\tilde{v}$: 3459, 3360, 3184, 1601, 1482, 1154, 777, 646 cm^{-1}.

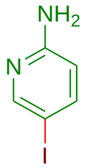

2ag

1-Chloronaphthalen-2-ol (2ah) [25]: White solid, 160 mg (90%), m.p. 65-67 °C (lit. m.p. 64-66 °C); ^{1}H NMR (500 MHz, $CDCl_3$): δ (ppm) 5.94 (s, 1H), 7.27 (d, J = 8.8 Hz, 1H), 7.41 (t, J = 8.6 Hz, 1H), 7.58 (t, J = 8.6 Hz, 1H), 7.71 (d, J = 8.8 Hz, 1H), 7.80 (d, J = 8.8 Hz, 1H), 8.07 (d, J = 8.6 Hz, 1H). ^{13}C NMR (125 MHz, $CDCl_3$): δ (ppm) 113.3, 117.2, 122.7, 124.1, 127.5, 128.2, 128.4, 129.4, 131.0, 149.3. Anal. Calcd for $C_{10}H_7ClO$: C, 67.25; H, 3.95. Found: C, 67.21; H, 3.92. IR (KBr) $\tilde{v}$: 3300, 1590, 1494, 1232, 1093, 812, 644 cm^{-1}.

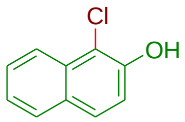

2ah

5-Chloro-2-hydroxybenzaldehyde (2ai) [26]: White solid, 115 mg (74%), m.p. 100-102 °C (lit. m.p. 98-100 °C); ^{1}H NMR (500 MHz, $CDCl_3$): δ (ppm) 6.94 (d, J = 8.5 Hz, 1H), 7.45 (dd, J_1 = 3.0 Hz, J_2 = 8.6 Hz, 1H), 7.52 (d, J = 3.0 Hz, 1H), 9.83 (s, 1H), 10.90 (s, 1H). ^{13}C NMR (125 MHz, $CDCl_3$): δ (ppm) 119.3, 121.1, 124.6, 132.6, 136.8, 160.1, 195.6. Anal. Calcd for $C_7H_5ClO_2$: C, 53.70; H, 3.22. Found: C, 53.64; H, 3.18. IR (KBr) $\tilde{v}$: 3220, 3038, 2876, 1686, 1471, 1265, 1160, 898, 697, 635 cm^{-1}.

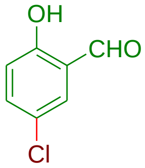

2ai

2-Amino-5-Chloropyridine (2aj) [27]: Brown solid, 102 mg (80%), m.p. 135-137 °C (lit. m.p. 136-137 °C); ^{1}H NMR (500 MHz, $CDCl_3$): δ (ppm) 4.57 (s, 2H), 6.43 (d, J = 8.8 Hz, 1H), 7.35 (dd, J_1 = 2.6 Hz, J_2 = 8.7 Hz, 1H), 7.99 (d, J = 2.6 Hz, 1H). ^{13}C NMR (125 MHz, $CDCl_3$): δ (ppm) 109.4, 120.8, 137.5, 146.3, 156.8. Anal. Calcd for $C_5H_5ClN_2$: C, 46.71; H, 3.92; N, 21.79. Found: C, 46.60; H, 3.84; N, 21.63. IR (KBr) $\tilde{\nu}$: 3459, 3296, 3148, 1624, 1476, 821, 754, 649 cm^{-1}.

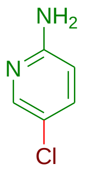

2aj

2,6-Dibromo-4-methylphenol (3a) [1]: Off-white solid, 231 mg (87%), m.p. 47-49 °C (lit. m.p. 49-50 °C); ^{1}H NMR (400 MHz, $CDCl_3$): δ (ppm) 2.18 (s, 3H), 5.65 (s, 1H), 7.18 (s, 2H). ^{13}C NMR (100 MHz, $CDCl_3$): δ (ppm) 20.0, 109.4, 132.4, 147.1. Anal. Calcd for $C_7H_6Br_2O$: C, 31.62; H, 2.27. Found: C, 31.60; H, 2.28. IR (KBr) $\tilde{\nu}$: 3420, 2922, 1560, 1476, 1323, 1273, 1231, 1163, 851, 775, 739, 706, and 563 cm^{-1}.

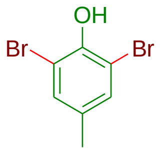

3a

2,4-Dibromonaphthalen-1-ol (3b) [28]: Light brown solid, 244 mg (81%), m.p. 105-106 °C (lit. m.p. 105-107 °C); ^{1}H NMR (400 MHz, $CDCl_3$): δ (ppm) 5.97 (s, 1H), 7.57 (td, J_1 = 1.2. Hz, J_2 = 6.8 Hz, 1H), 7.63 (td, J_1 = 1.2. Hz, J_2 = 6.8 Hz, 1H), 7.79 (s, 1H), 8.13 (d, J = 8.0 Hz, 1H), 8.25 (d, J = 7.6 Hz, 1H). ^{13}C NMR (100 MHz, $CDCl_3$): δ (ppm) 103.2, 113.3, 122.8, 125.1, 126.9, 127.1, 128.1, 131.1, 131.9, 148.2. Anal. Calcd for $C_{10}H_6Br_2O$: C, 39.78; H, 2.00. Found: C, 39.70; H, 2.01. IR (KBr) $\tilde{\nu}$: 3283, 3070, 1630, 1490, 1348, 1236, 985, 808, 644 and 575 cm^{-1}.

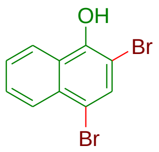

3b

2,4-Dibromo-6-nitrophenol (3c) [29]: Yellow solid, 235 mg (79%), m.p. 107-109 °C (lit. m.p. 110 °C); ^{1}H NMR (400 MHz, $CDCl_3$): δ (ppm) 7.98 (d, J = 2.4 Hz, 1H), 8.24 (d, J = 2.4 Hz, 1H), 11.04 (s, 1H). ^{13}C NMR (100 MHz, $CDCl_3$): δ (ppm) 112.3, 114.5, 126.7, 134.4, 142.9, 151.4. Anal. Calcd for $C_6H_3Br_2NO_3$: C, 24.27; H, 1.02; N, 4.72. Found: C, 24.17; H, 1.04; N, 4.65. IR (KBr) $\tilde{\nu}$: 3338, 3372, 3098, 1624, 1510, 1439, 1312, 1121, 893, 746, 638 and 545 cm^{-1}.

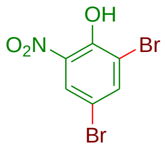

3c

3,5-Dibromo-4-hydroxybenzonitrile (3d) [30]: Off-white solid, 235 mg (85%), m.p. 185-186 °C (lit. m.p. 190-192 °C); ^{1}H NMR (400 MHz, $CDCl_3$): δ (ppm) 6.42 (br, 1H), 7.77 (s, 2H). ^{13}C NMR (100 MHz, $CDCl_3$): δ (ppm) 106.5, 110.4, 116.2, 135.6, 153.6. Anal. Calcd for $C_7H_3Br_2NO$: C, 30.36; H, 1.09; N, 5.06. Found: C, 30.30; H, 1.09; N, 5.03. IR (KBr) $\tilde{\nu}$: 3438, 3089, 2228, 1661, 1428, 1121, 893, 746, 638 and 575 cm^{-1}.

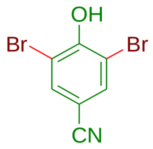

3d

2,6-Dibromo-4-chlorophenol (3e) [31]: White solid, 245 mg (85%), m.p. 84-86 °C (lit. m.p. 79-84 °C); ^{1}H NMR (400 MHz, $CDCl_3$): δ (ppm) 5.85 (s, 1H), 7.46 (s, 2H). ^{13}C NMR (100 MHz, $CDCl_3$): δ (ppm) 109.9, 126.2, 131.6, 148.5. Anal. Calcd for $C_6H_3Br_2ClO$: C, 25.17; H, 1.06. Found: C, 25.20; H, 1.06. IR (KBr) $\tilde{\nu}$: 3483, 3076, 2924, 1558, 1456, 1387, 1312, 1273, 1217, 1155, 856, 738, 713 and 555 cm^{-1}.

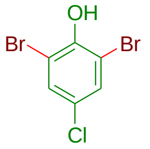

3e

2,4-Dibromo-6-chlorophenol (3f) [29]: White solid, 240 mg (84%), m.p. 87-89 °C (lit. m.p. 92 °C); ^{1}H NMR (400 MHz, $CDCl_3$): δ (ppm) 5.90 (s, 1H), 7.45 (d, J = 2.4 Hz, 1H), 7.55 (d, J = 2.0 Hz, 1H). ^{13}C NMR (100 MHz, $CDCl_3$): δ (ppm) 110.8, 112.2, 121.5, 131.4, 133.6, 148.2. Anal. Calcd for $C_6H_3Br_2ClO$: C, 25.17; H, 1.06. Found: C, 25.26; H, 1.03. IR (KBr) $\tilde{\nu}$: 3460, 3075, 2924, 1560, 1460, 1385, 1312, 1271, 1231, 1153, 855, 770, 691 and 556 cm^{-1}.

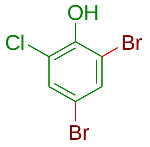

3f

2,4,6-Tribromophenol (3g) [29]: Pale-white solid, 265 mg (80%), m.p. 91-93 °C (lit. m.p. 95 °C); ^{1}H NMR (400 MHz, $CDCl_3$): δ (ppm) 5.90 (s, 1H), 7.59 (s, 2H). ^{13}C NMR (100 MHz, $CDCl_3$): δ (ppm) 110.4, 112.7, 134.2, 148.9. Anal. Calcd for $C_6H_3Br_3O$: C, 21.79; H, 0.91. Found: C, 21.88; H, 0.94. IR (KBr) $\tilde{\nu}$: 3447, 3071, 1546, 1458, 737, 671, and 552 cm^{-1}.

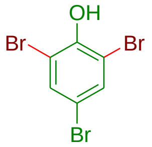

3g

2,6-Dibromo-4-iodophenol (3h) [32]: White solid, 317 mg (84%), m.p. 98-99 °C (lit. m.p. 99-101 °C); ^{1}H NMR (400 MHz, $CDCl_3$): δ (ppm) 5.91 (br, 1H), 7.74 (s, 2H). ^{13}C NMR (100 MHz, $CDCl_3$): δ (ppm) 81.7, 110.8, 139.7, 149.6. Anal. Calcd for $C_6H_3Br_2IO$: C, 19.08; H, 0.80. Found: C, 19.14; H, 0.82. IR (KBr) $\tilde{\nu}$: 3414, 3061, 1547, 1377, 1263, 856, 735, 648, and 552 cm^{-1}.

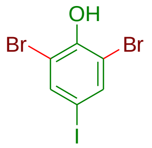

3h

2,6-Diiodo-4-methylphenol (3i) [19]: Pale-yellow solid, 306 mg (85%), m.p. 48-50 °C (lit. m.p. 49-51 °C); ^{1}H NMR (400 MHz, $CDCl_3$): δ (ppm) 2.22 (s, 3H), 5.57 (s, 1H), 7.49 (s, 1H). ^{13}C NMR (100 MHz, $CDCl_3$): δ (ppm) 19.4, 81.9, 133.9, 139.6, 151.4. Anal. Calcd for $C_7H_6I_2O$: C, 23.36; H, 1.68. Found: C, 23.30; H, 1.64. IR (KBr) $\tilde{\nu}$: 3449, 2918, 1543, 1456, 1150, 853, 766, 710 and 556 cm^{-1}.

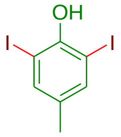

3i

2,6-Dibromo-4-methylaniline (3j) [2,29]: Off-white solid, 233 mg (88%), m.p. 73-75 °C (lit. m.p. 74-76 °C); ^{1}H NMR (400 MHz, $CDCl_3$): δ (ppm) 2.21 (s, 3H), 4.32 (br, 2H), 7.20 (s, 2H). ^{13}C NMR (100 MHz, $CDCl_3$): δ (ppm) 19.8, 108.8, 129.4, 132.2, 139.4. Anal. Calcd for $C_7H_7Br_2N$: C, 31.73; H, 2.66; N, 5.29. Found: C, 31.63; H, 2.65; N, 5.27. IR (KBr) $\tilde{\nu}$: 3487, 3380, 2942, 1570, 1486, and 682 cm^{-1}.

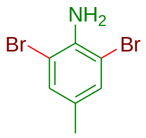

3j

2,4,6-Tribromoaniline (4a) [29]: White solid, 294-310 mg (90-95%), m.p. 121-122 °C (lit. m.p. 121-122 °C); ^{1}H NMR (400 MHz, $CDCl_3$): δ (ppm) 4.50 (br, 2H), 7.50 (s, 2H). ^{13}C NMR (100 MHz, $CDCl_3$): δ (ppm) 108.8, 133.8, 141.3. Anal. Calcd for $C_6H_4Br_3N$: C, 21.85; H, 1.22; N, 4.25. Found: C, 21.81; H, 1.24; N, 4.29. IR (KBr) $\tilde{\nu}$: 3414, 3287, 3073,1456, 1381, 1067, 860, 733, 706, 671 and 548 cm^{-1}.

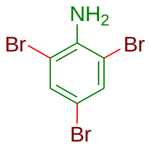

4a

2,4,6-Triiodophenol (4b) [33]: White solid, 439-448 mg (93-95%), m.p. 150-151 °C (lit. m.p. 148 °C); ^{1}H NMR (400 MHz, $CDCl_3$): δ (ppm) 5.76 (s, 1H), 7.93 (s, 2H). ^{13}C NMR (100 MHz, $CDCl_3$): δ (ppm) 83.28, 83.33, 146.4, 153.7. Anal. Calcd for $C_6H_3I_3O$: C, 15.27; H, 0.64. Found: C, 15.30; H, 0.63. IR (KBr) $\tilde{\nu}$: 3447, 3047, 1437, 1371, 1138, 860, 700, 650, and 542 cm^{-1}.

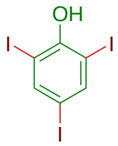

4b

2,4,6-Triiodoaniline (4c) [34]: Off-white solid, 447-456 mg (92-97%), m.p. 175-177 °C (lit. m.p. 175-176 °C); ^{1}H NMR (400 MHz, $CDCl_3$): δ (ppm) 4.66 (br, 2H), 7.86 (s, 2H). ^{13}C NMR (100 MHz, $CDCl_3$): δ (ppm) 78.7. 81.8, 146.2, 149.0. Anal. Calcd for $C_6H_4I_3N$: C, 15.31; H, 0.86; N, 2.98. Found: C, 15.38; H, 0.85; N, 2.90. IR (KBr) $\tilde{\nu}$: 3396, 3306, 1607, 1437, 1051, 860, 698 and 538 cm^{-1}.

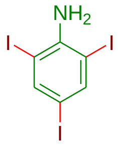

4c

Solution phase study

Table S1: Optimization of reaction condition for bromination under conventional conditions[a]

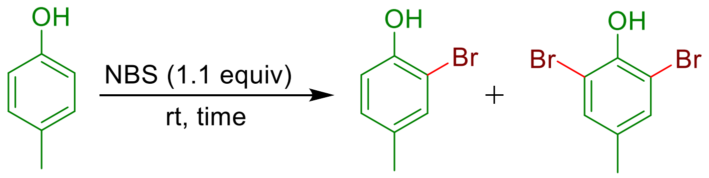

Entry	Solvent	Solvent Volume	Time (h)	Yield[b] (%) 1a	2a	3a
1	CH_3CN	1 mL	05	20	36	23
2	EtOH	1 mL	05	16	42	20
3	H_2O	1 mL	05	31	20	11
4	EtOH:H_2O (1:1)	1 mL	05	23	37	12
5	Ethylene glycol	1 mL	05	10	51	13
6	PEG-400	1 mL	2.5	NA	63	10
7	PEG-400	2 mL	2.5	NA	61	11
8	PEG-400	0.2 mL	2.5	55[c]	15	03

[a]1 mmol of **1a** and 1.1 mmol NBS are taken for stirring; [b]Isolated yields; [c]sticky mass resulted in inefficient stirring.

Comparative Table S2: Comparison of reported method with our method for aromatic halogenation with NXS.

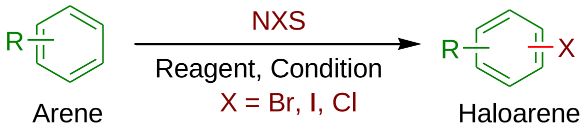

Sl. No.	Method	Solvent	Catalyst (mol %)/ Additive (equiv)	Condition	X	Yield (%)	E-factor	Ref
1	Solution phase	ACN	–	rt, 30 min to 6 h	Br	89–97	(16.5–20.2) x 10^3	35
2	Solution phase	CAN	–	0 °C to rt, 18 h	Br	18–99	(3.6–21.7) x 10^3	36
3	Solution phase	CAN	PTSA (0.5-2)	rt, 2 h	Br, Cl	33–91	(77.4–279.3) x 10^3	37
4	Solution phase	CAN	Thiourea (5)	rt, 10 min to 64 h	Br, I, Cl	38–98	(58.1–159.7) x 10^3	38
5	Solution phase	CAN	I_2 (10)	rt–50 °C, 12–48 h	Br	78–99	(13.2–15.8) x 10^3	39
6	Solution phase	DCM	$AgNTf_2$ (7.5)	20–45 °C, 18 min to 7.3 h	I	67–99	25.9–33.3	23
7	Solution phase	DCE	$Pd(OAc)_2$ (5), PTSA (0.5)	70 °C, 12 h	Br, I, Cl	61–94	19.3–22.7	40
8	Solution phase	DCE	$[RhCp^*Cl_2]2$ (1-2.5), $AgSbF_6$ (4-10), PivOH (1.1) or $Cu(OAc)_2$ (2.2)	60–120 °C, 16–52 h	Br, I	35–99	23.1–87.5	41
9	Solution phase	DCM	DABCO (5)	rt, 1 h	Br, I, Cl	53–99	26.6–29.1	42
10	Solution phase	HFIP	–	rt (or 0–100 °C), 5 min to 16 h	Br, I, Cl	74–99	30.8–35.7	2
11	Milling	Neat milling	–	21 Hz, rt, 45 min to 2 h	Br, I, Cl	70–98	0.43–1.2	43
12	Milling	Neat milling	MCM41-SO_3H (0.2 g/mmol of substrate)	30 Hz, rt, 1–20 min	Br	92–96	1.2–1.4	44
13	Automated grinding	PEG-400	–	100 rpm, rt, 2–15 min	Br, I, Cl	67–98	2.1–3.6	present work

^{1}H NMR and ^{13}C NMR spectra of products

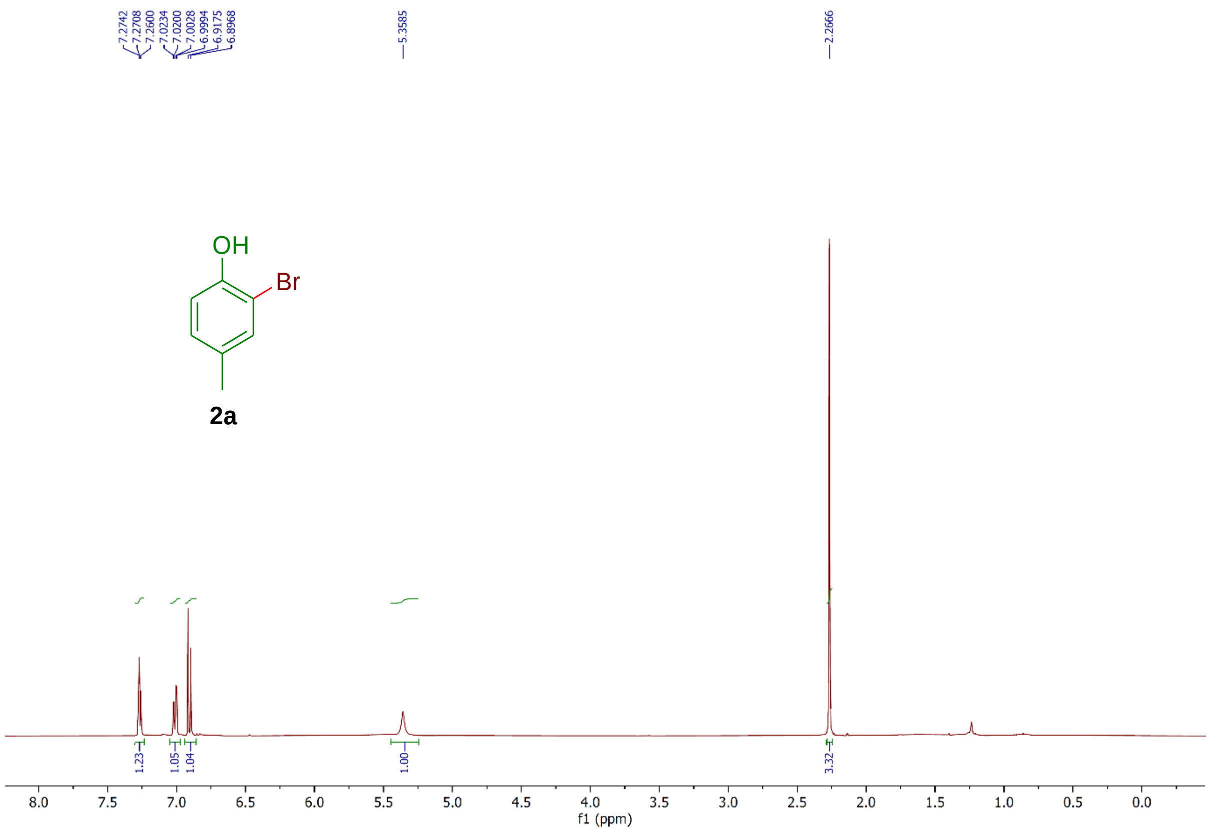

Figure S1: ^{1}H NMR spectrum of compound **2a**, ($CDCl_3$, 400 MHz).

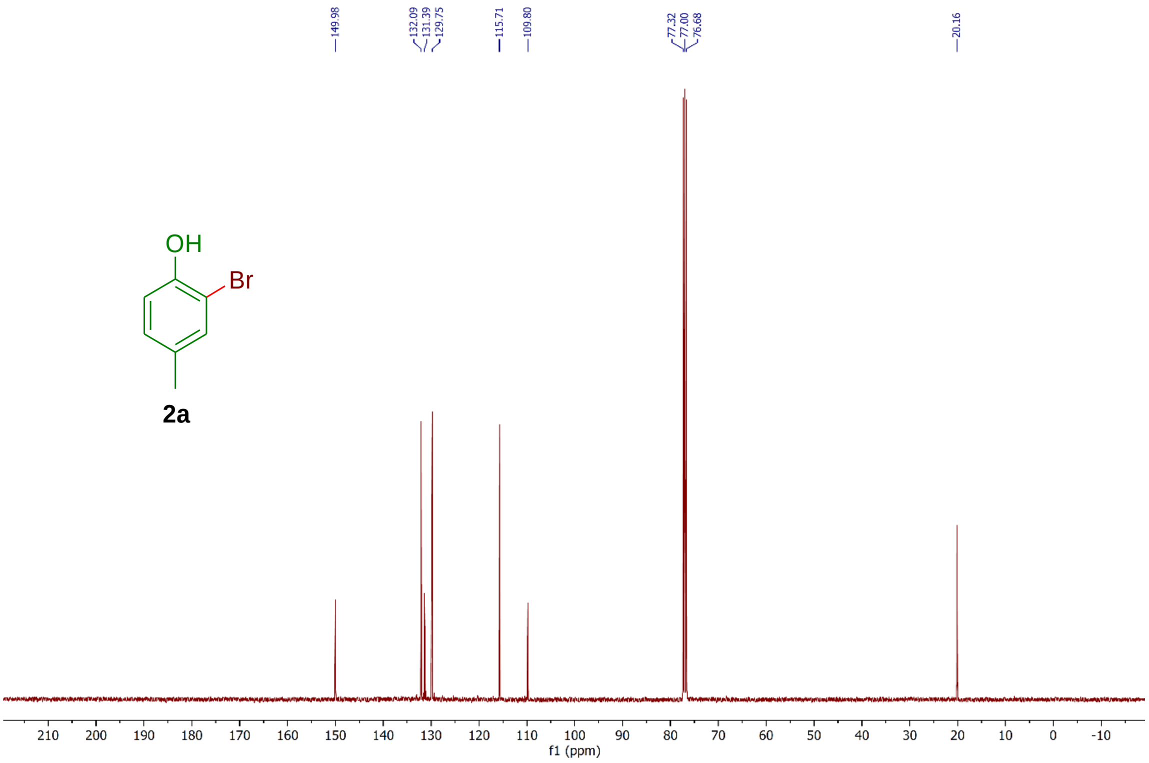

Figure S2: ^{13}C NMR spectrum of compound **2a**, ($CDCl_3$, 100 MHz).

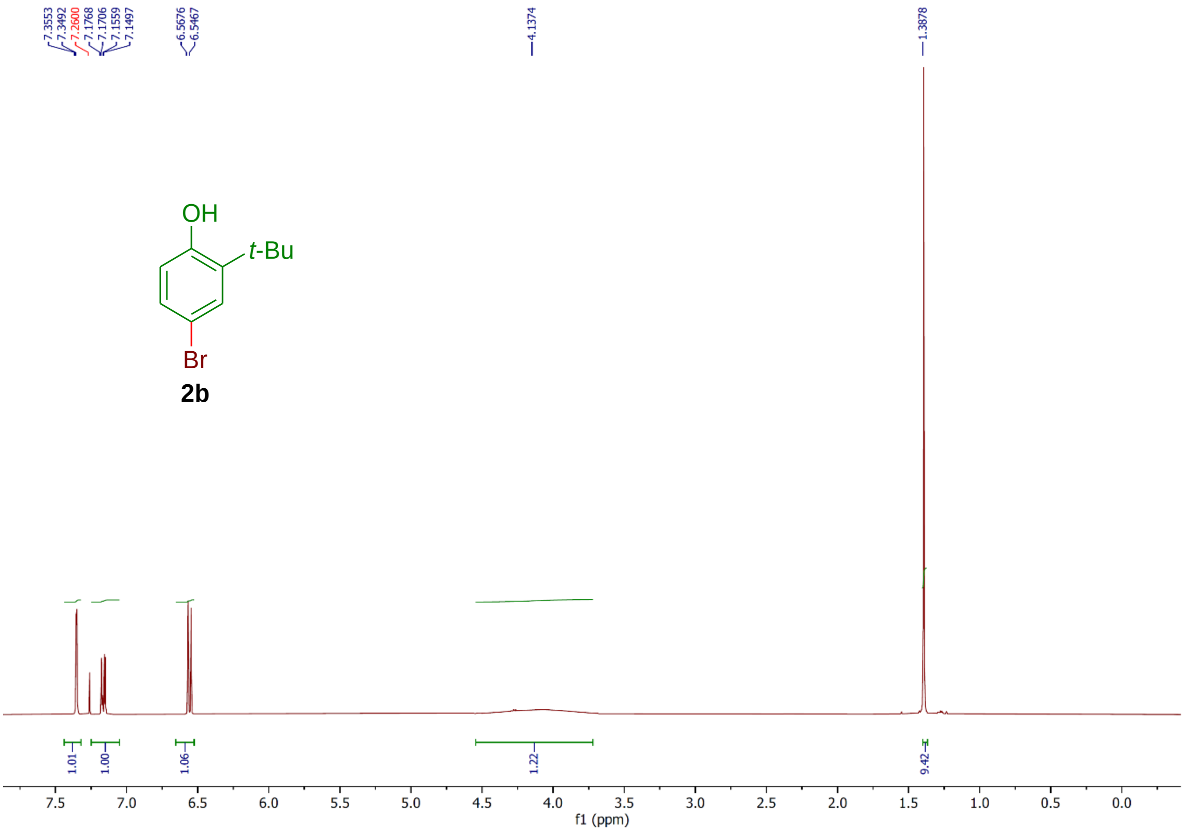

Figure S3: ^{1}H NMR spectrum of compound **2b**, ($CDCl_3$, 400 MHz).

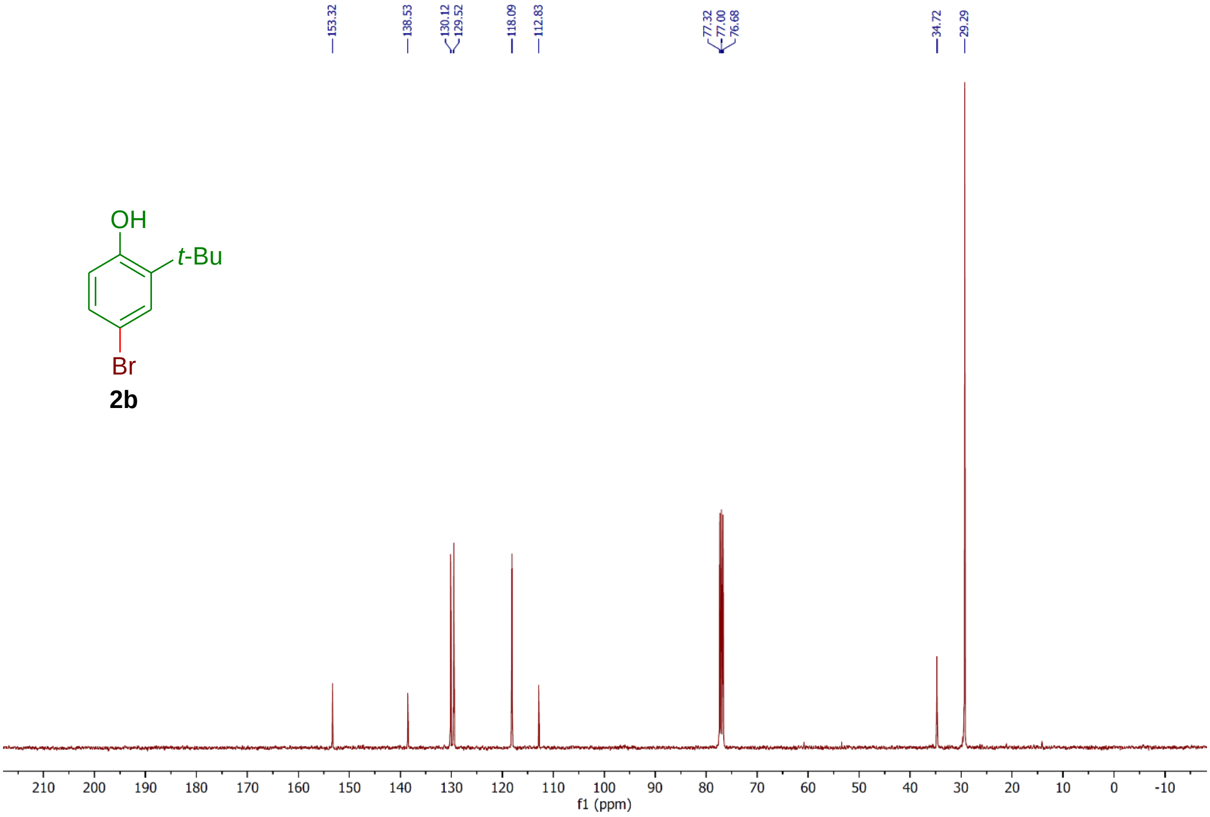

Figure S4: ^{13}C NMR spectrum of compound **2b**, ($CDCl_3$, 100 MHz).

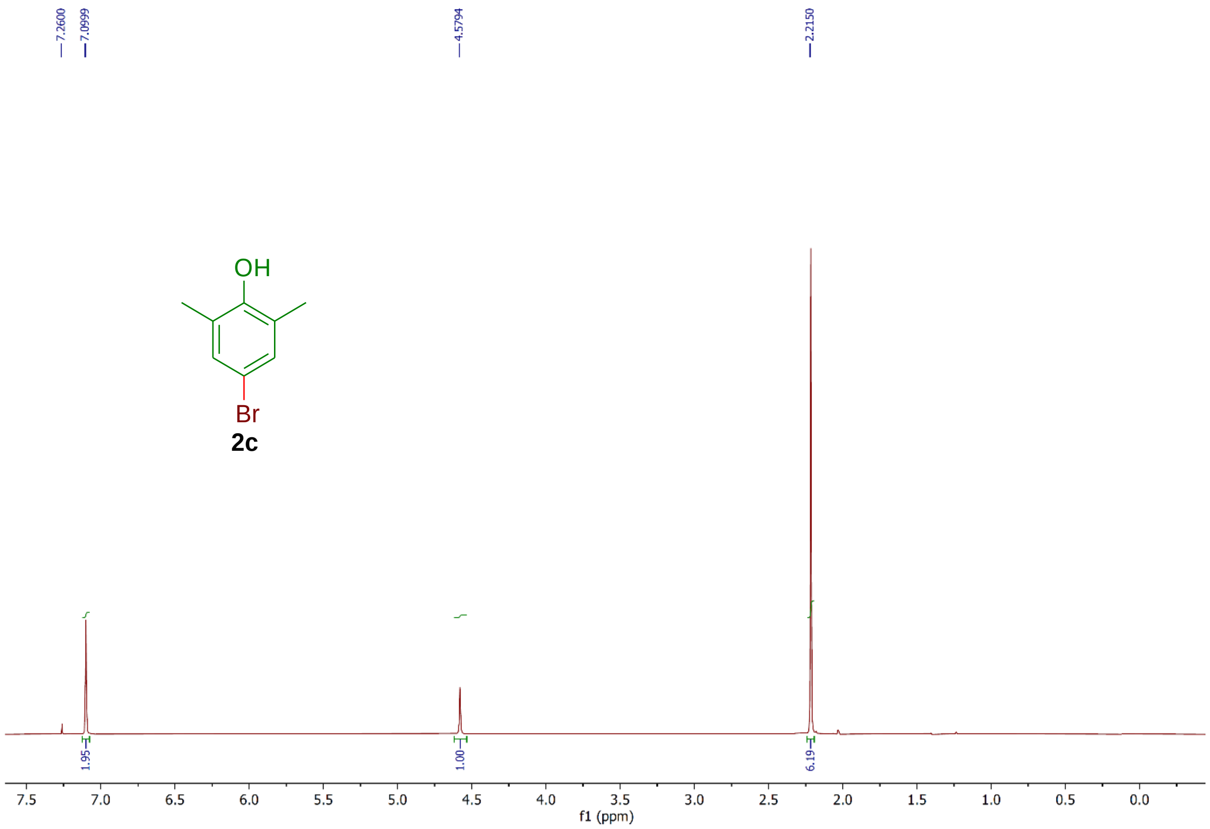

Figure S5: ^{1}H NMR spectrum of compound **2c**, ($CDCl_3$, 400 MHz).

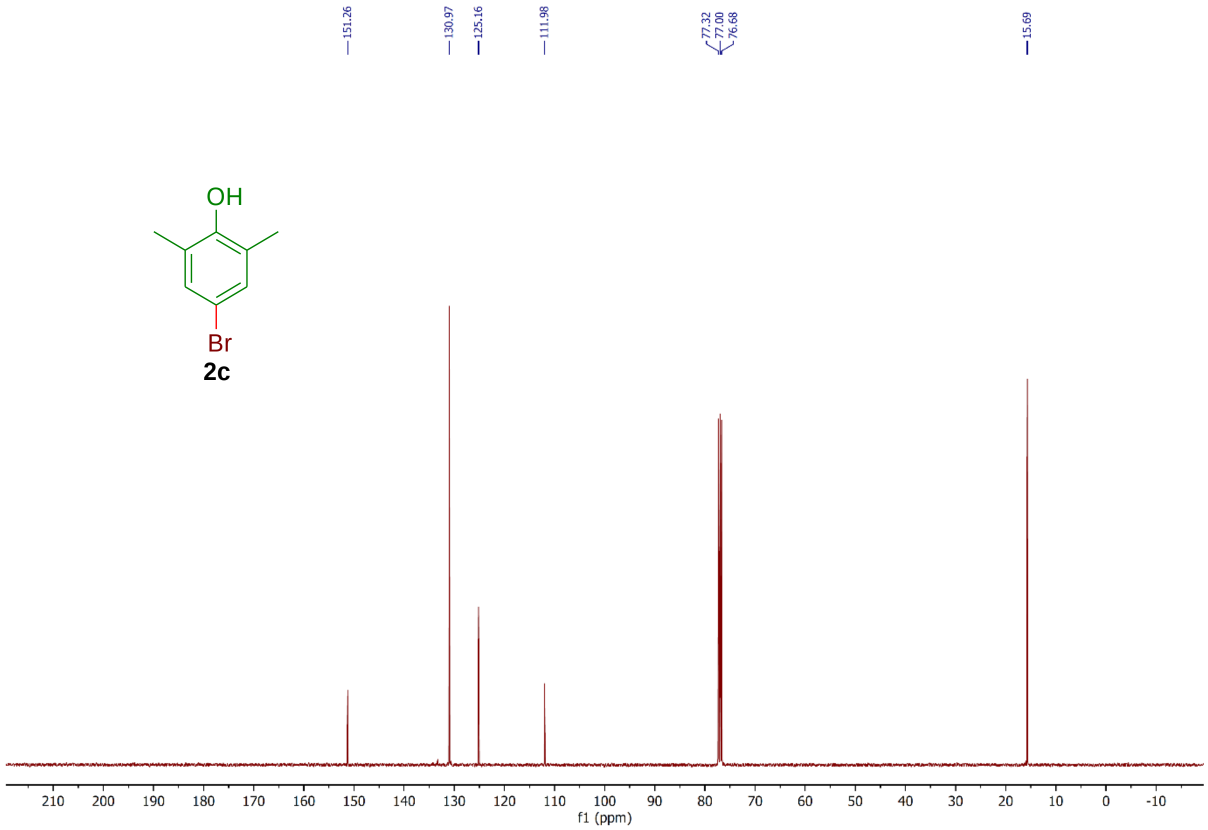

Figure S6: ^{13}C NMR spectrum of compound **2c**, ($CDCl_3$, 100 MHz).

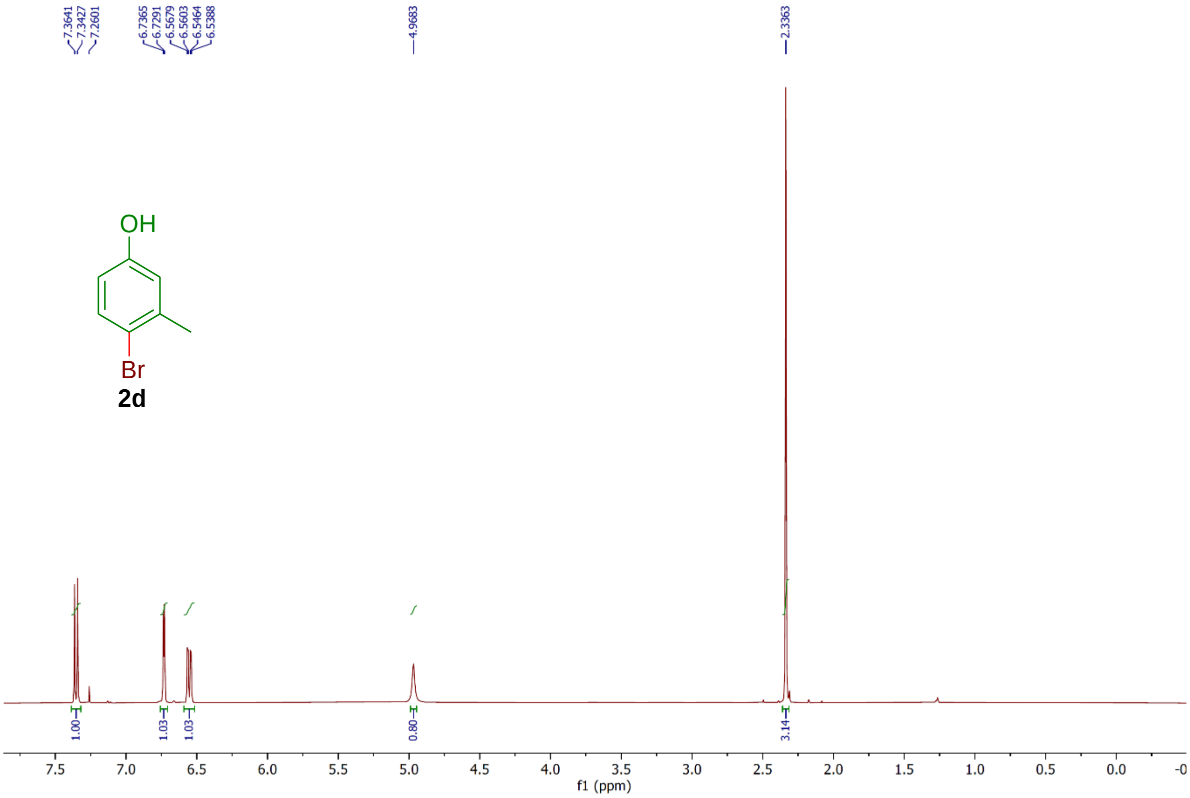

Figure S7: ^{1}H NMR spectrum of compound **2d**, ($CDCl_3$, 400 MHz).

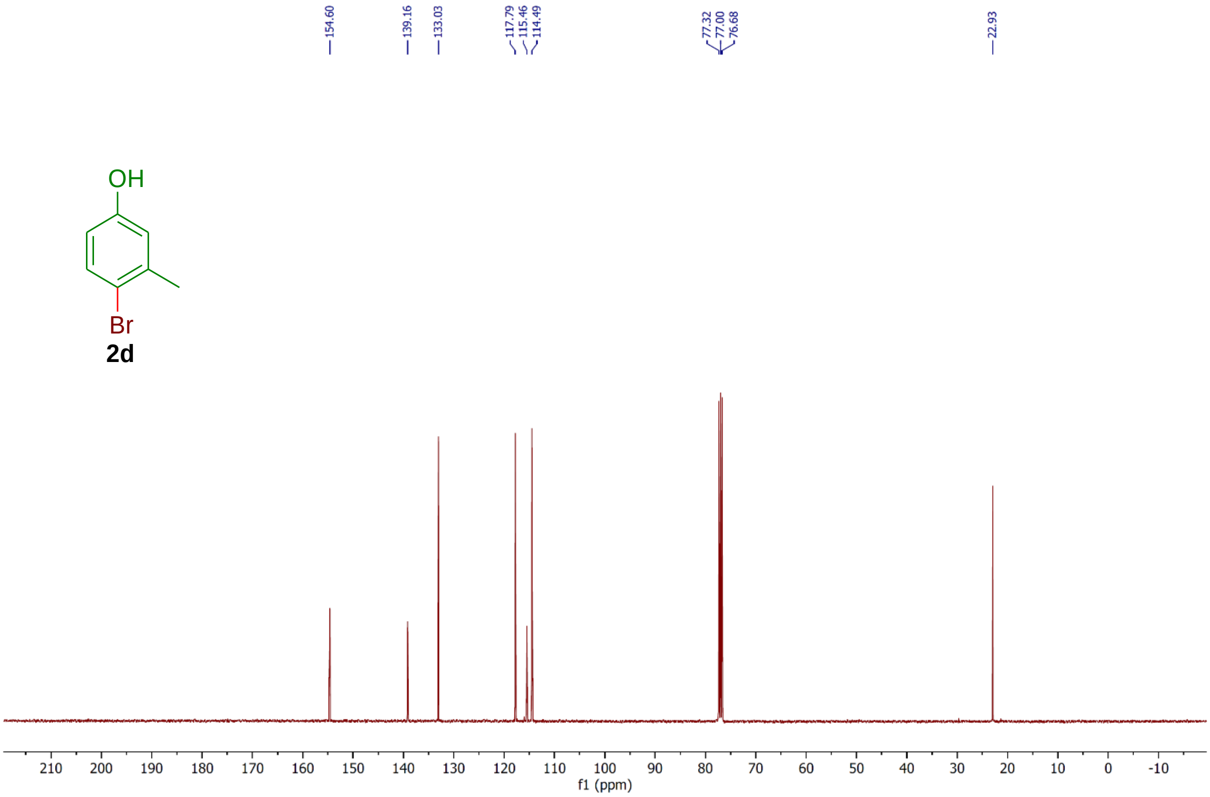

Figure S8: ^{13}C NMR spectrum of compound **2d**, ($CDCl_3$, 100 MHz).

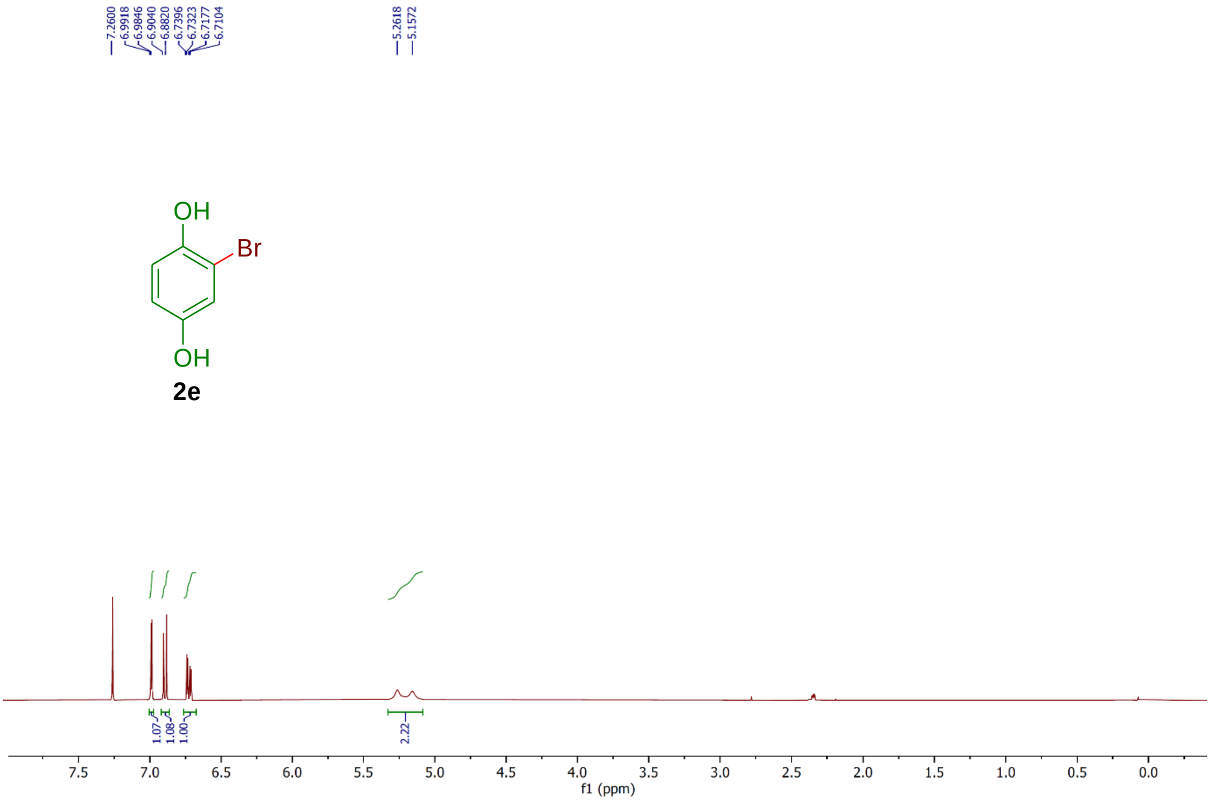

Figure S9: ^{1}H NMR spectrum of compound **2e**, ($CDCl_3$, 400 MHz).

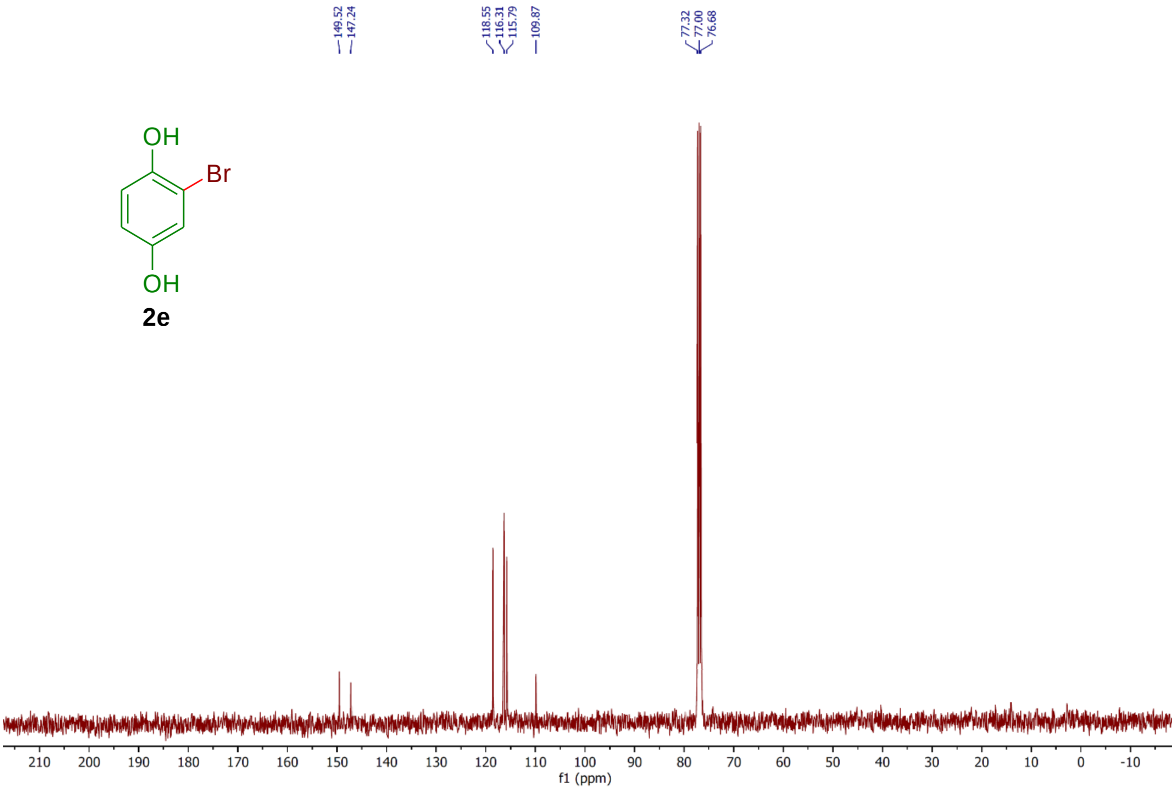

Figure S10: ^{13}C NMR spectrum of compound **2e**, ($CDCl_3$, 100 MHz).

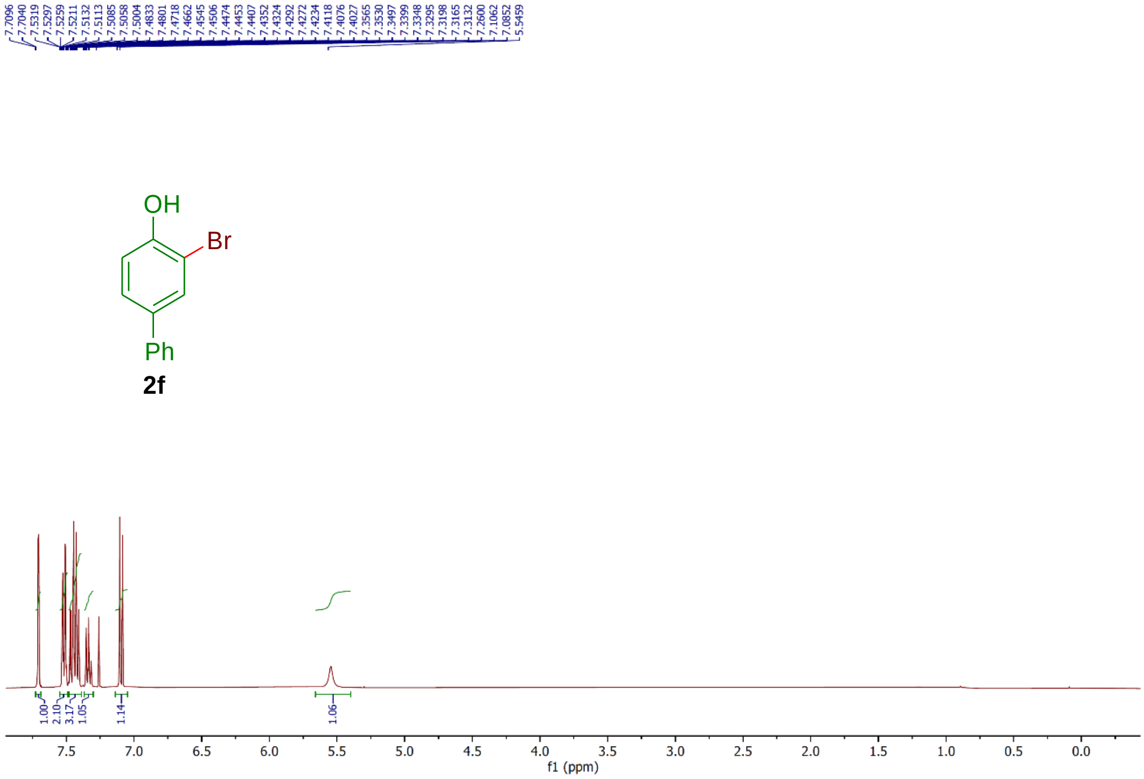

Figure S11: ^{1}H NMR spectrum of compound **2f**, ($CDCl_3$, 400 MHz).

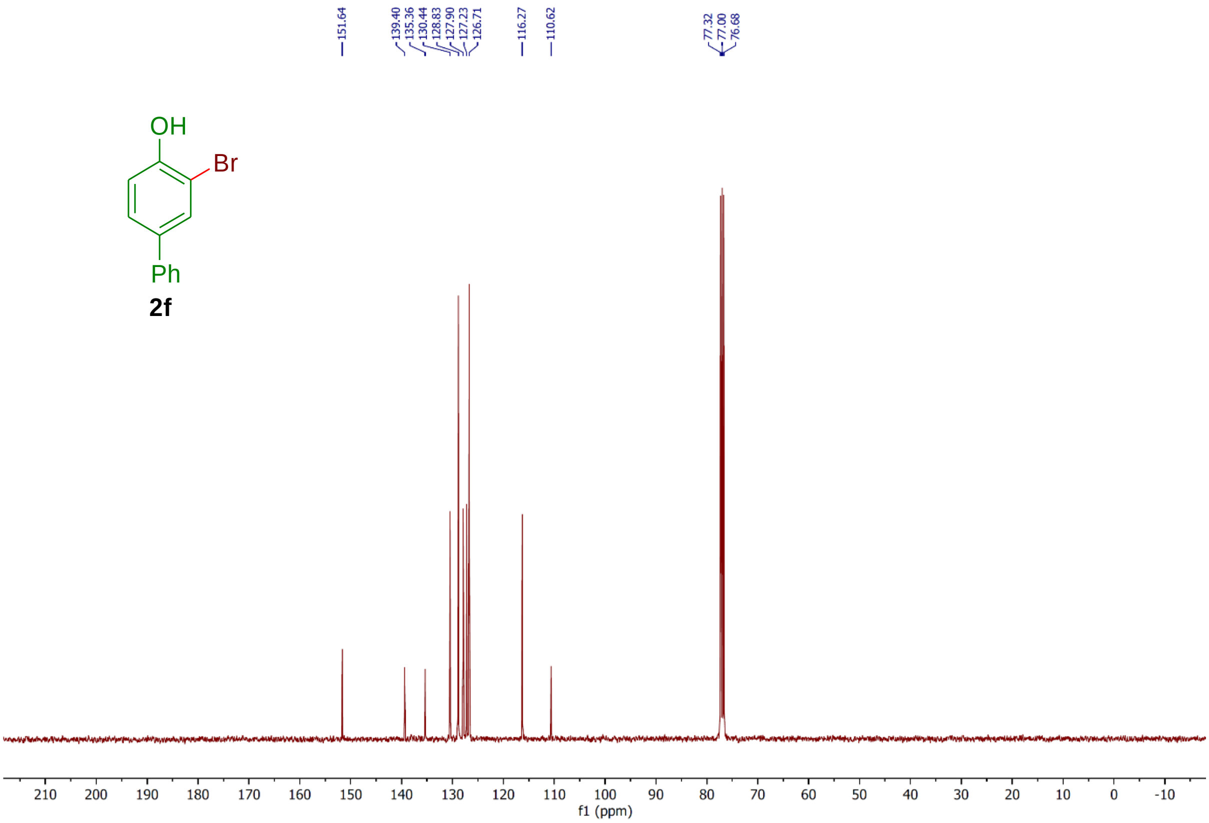

Figure S12: ^{13}C NMR spectrum of compound **2f**, ($CDCl_3$, 100 MHz).

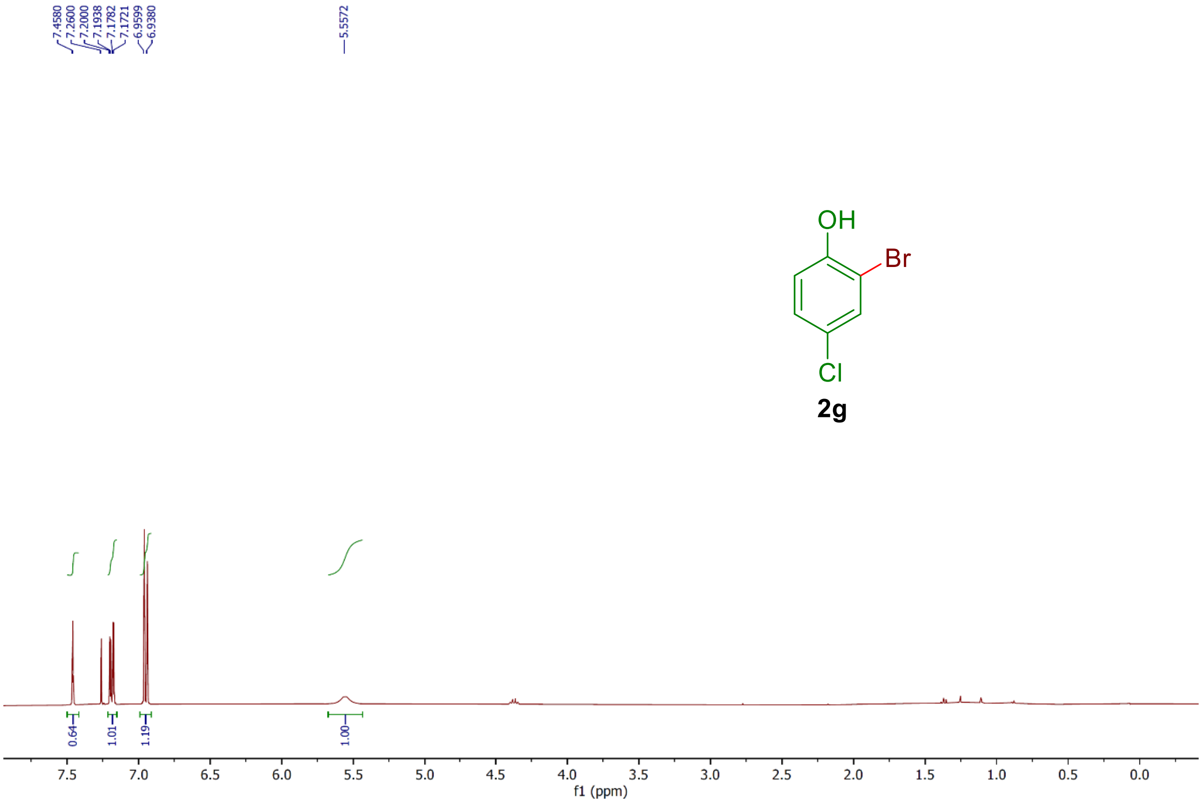

Figure S13: ^{1}H NMR spectrum of compound **2g**, ($CDCl_3$, 400 MHz).

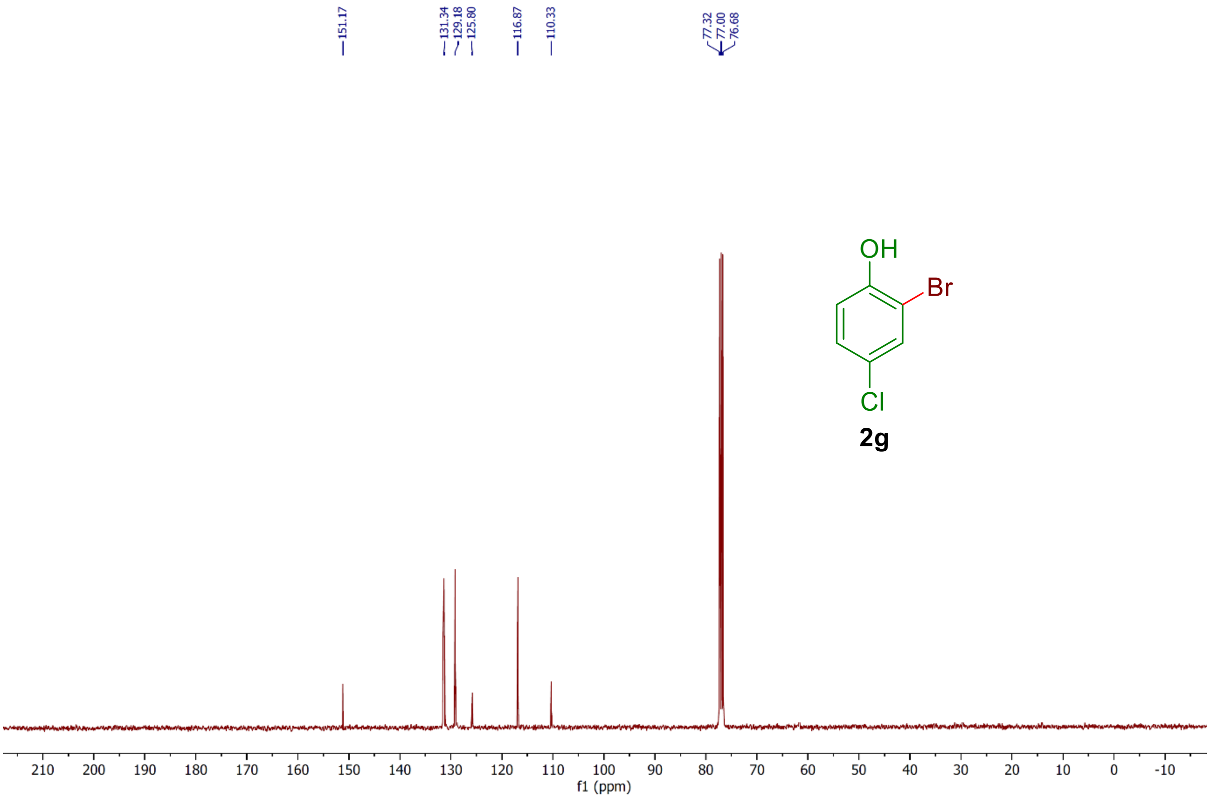

Figure S14: ^{13}C NMR spectrum of compound **2g**, ($CDCl_3$, 100 MHz).

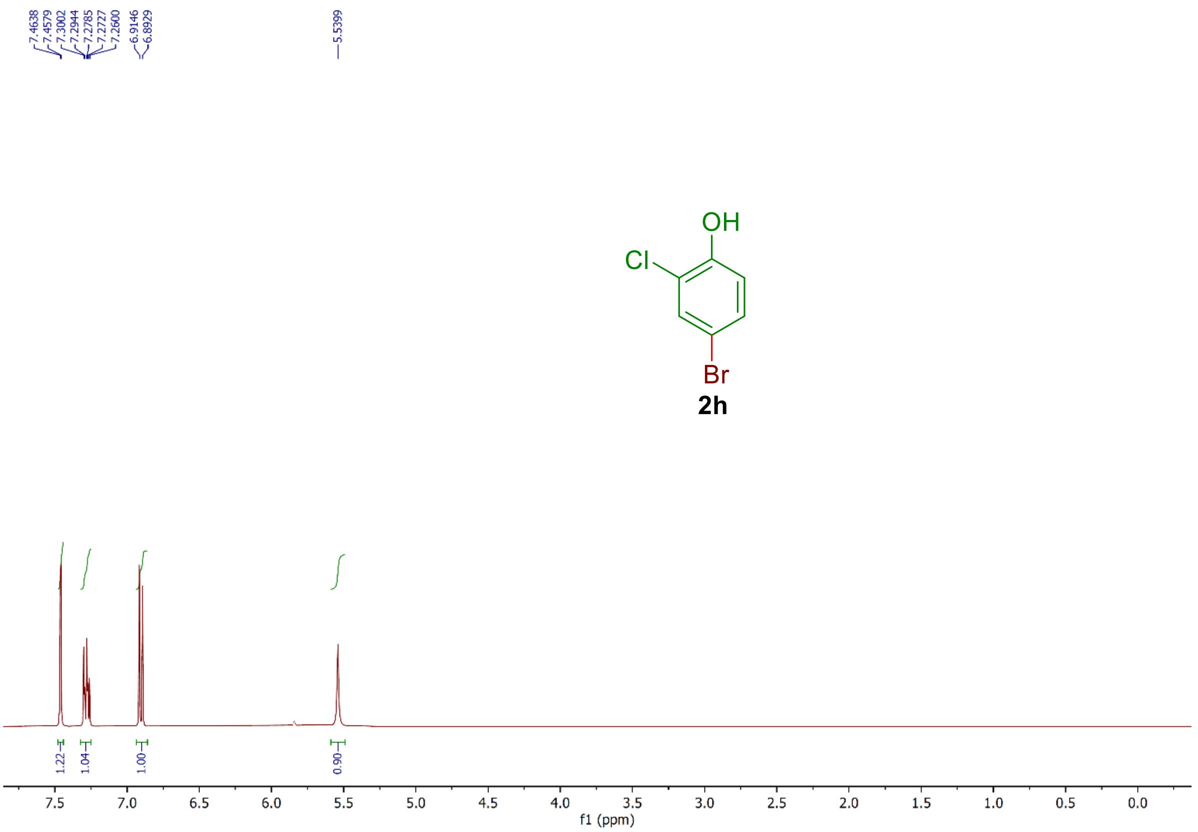

Figure S15: ^{1}H NMR spectrum of compound **2h**, ($CDCl_3$, 400 MHz).

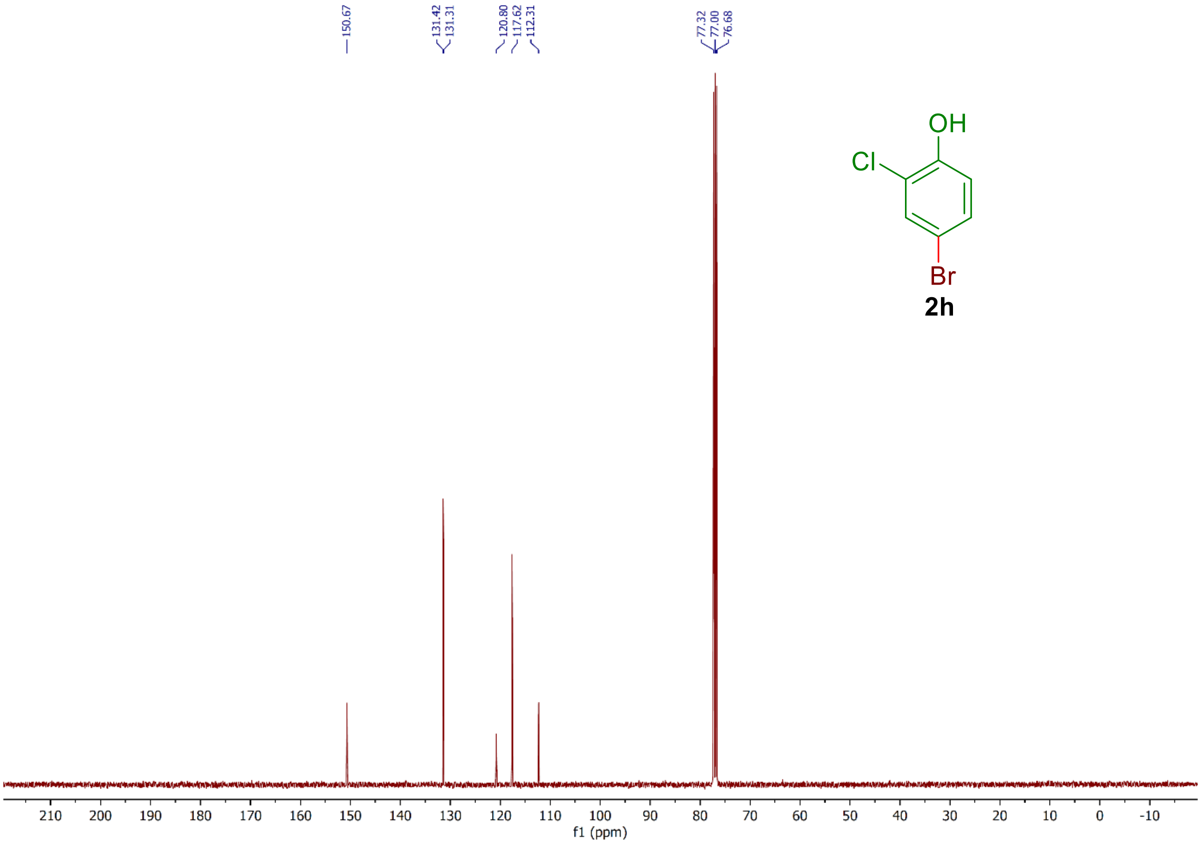

Figure S16: ^{13}C NMR spectrum of compound **2h**, ($CDCl_3$, 100 MHz).

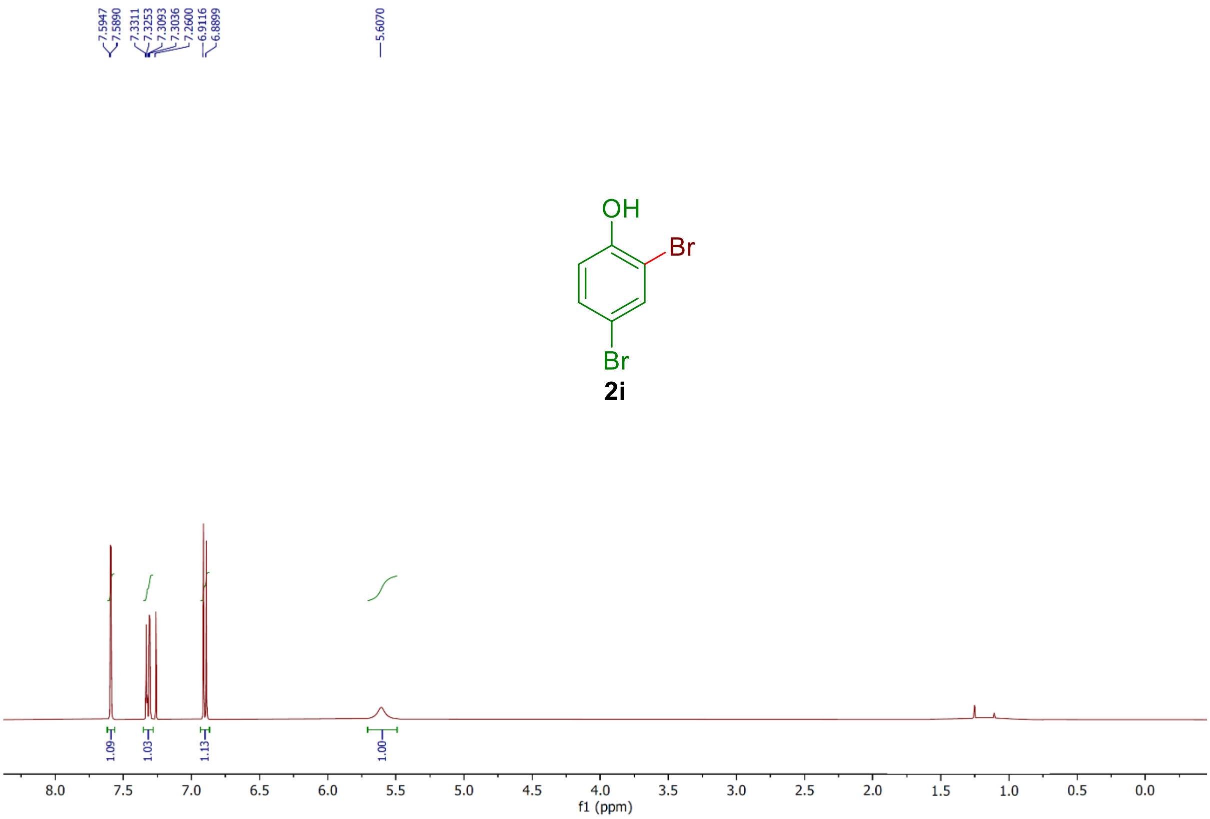

Figure S17: ^{1}H NMR spectrum of compound **2i**, ($CDCl_3$, 400 MHz).

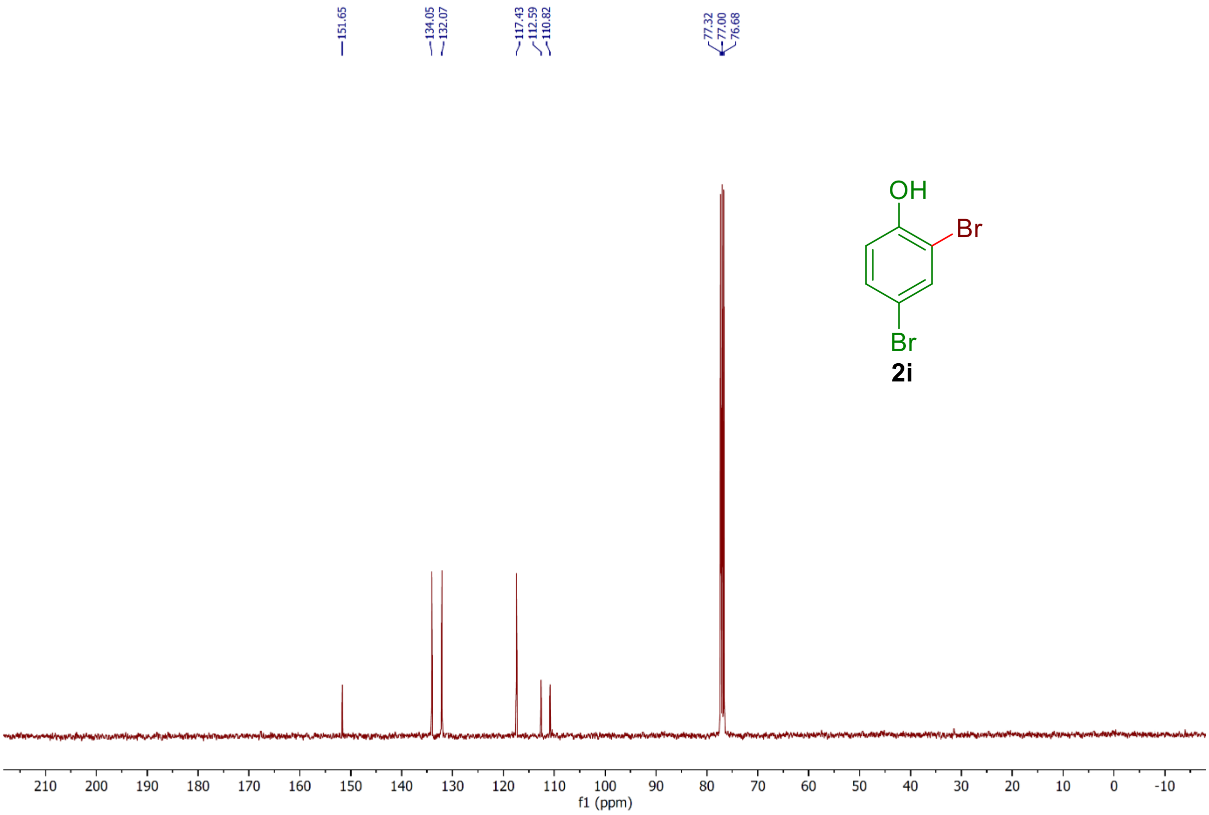

Figure S18: ^{13}C NMR spectrum of compound **2i**, ($CDCl_3$, 100 MHz).

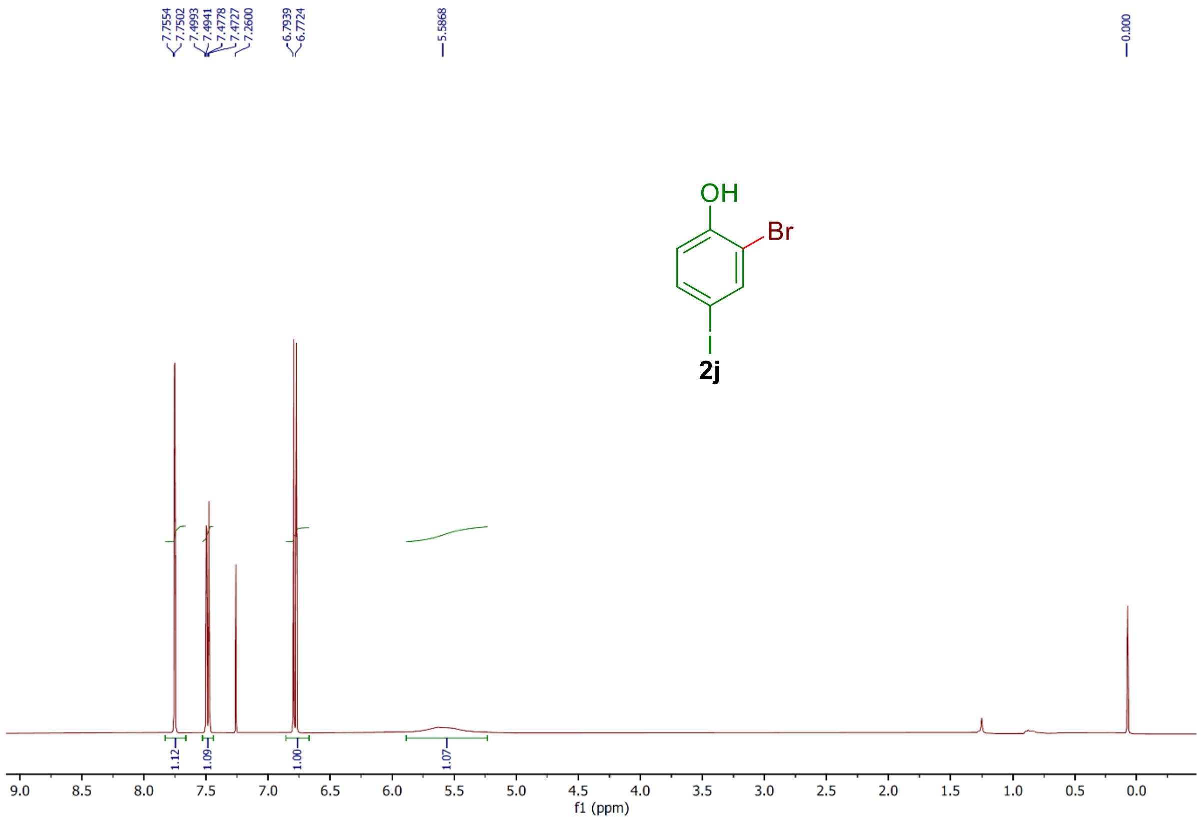

Figure S19: ^{1}H NMR spectrum of compound **2j**, ($CDCl_3$, 400 MHz).

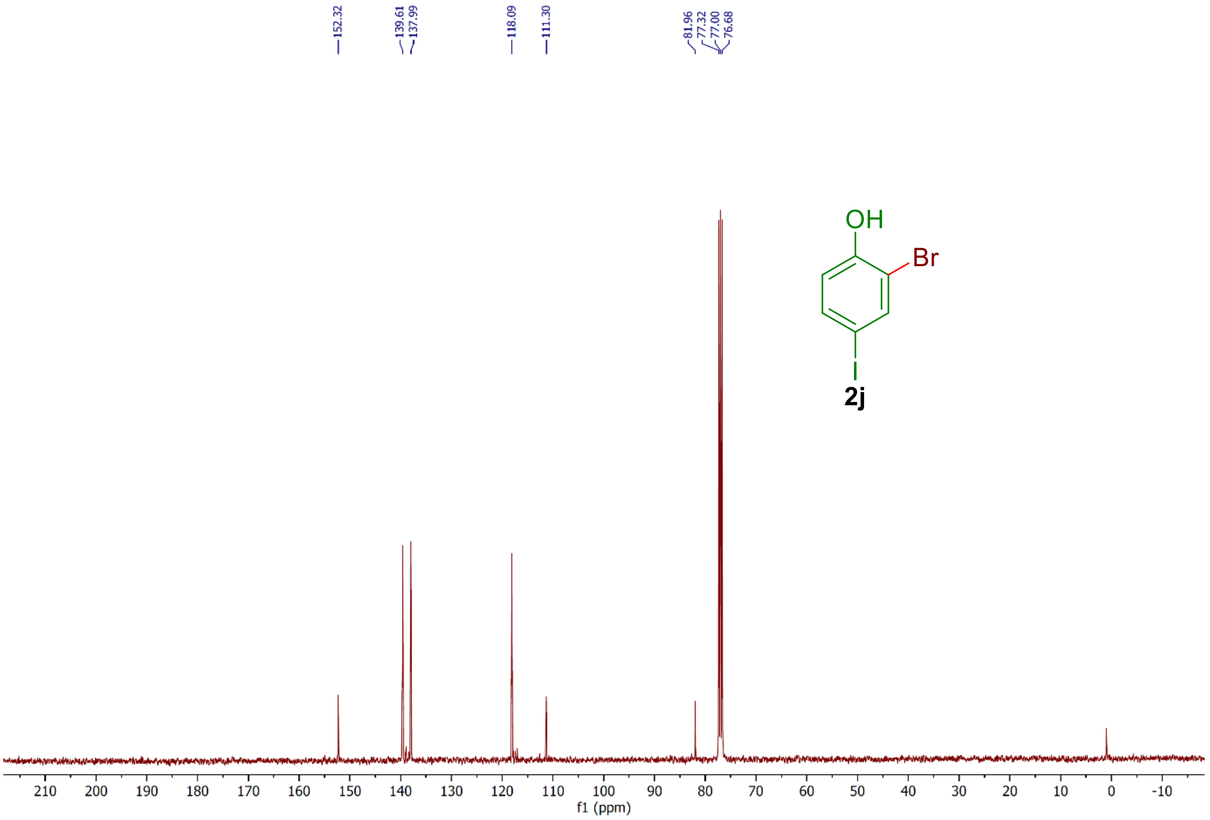

Figure S20: ^{13}C NMR spectrum of compound **2j**, ($CDCl_3$, 100 MHz).

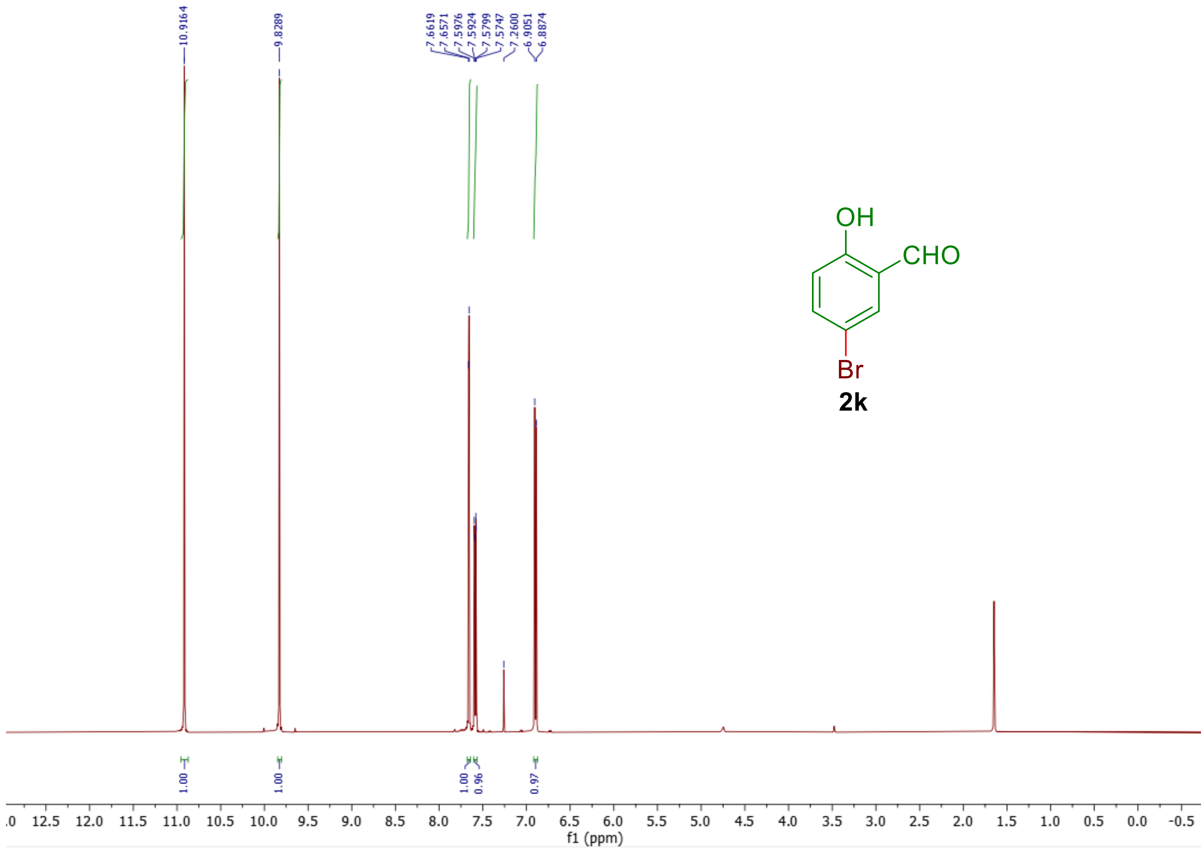

Figure S21: ^{1}H NMR spectrum of compound **2k**, ($CDCl_3$, 500 MHz).

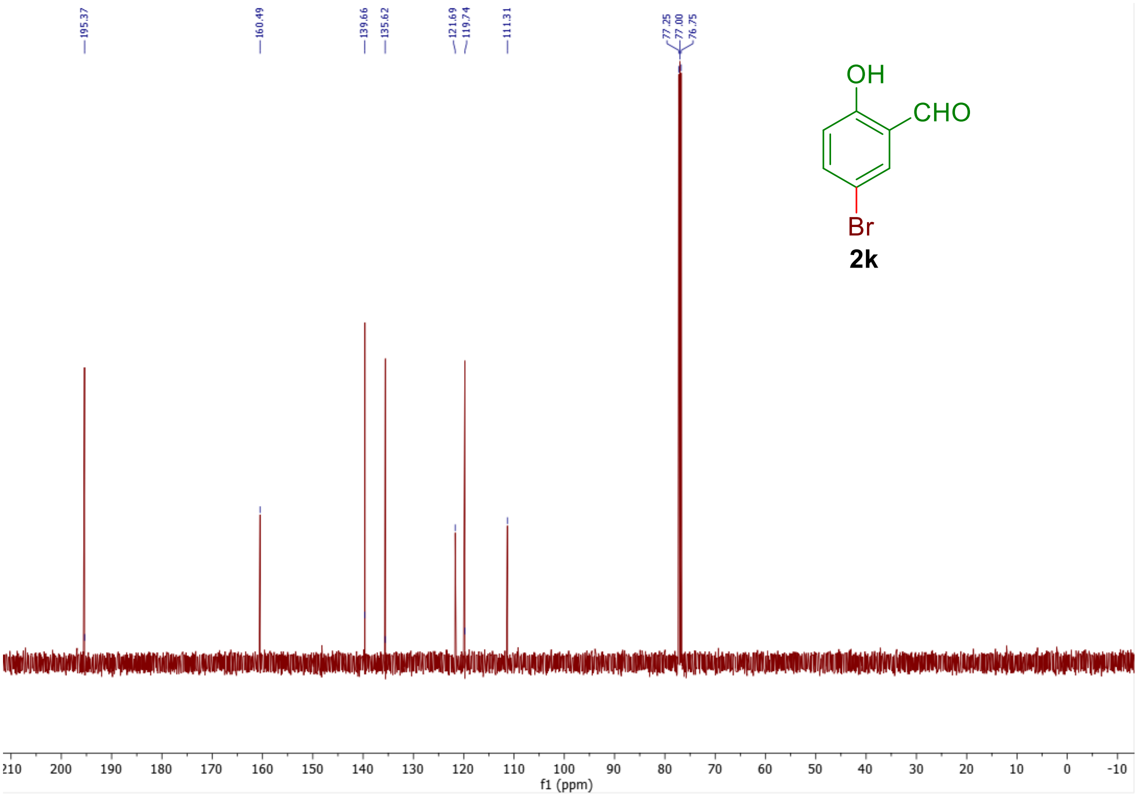

Figure S22: ^{13}C NMR spectrum of compound **2k**, ($CDCl_3$, 125 MHz).

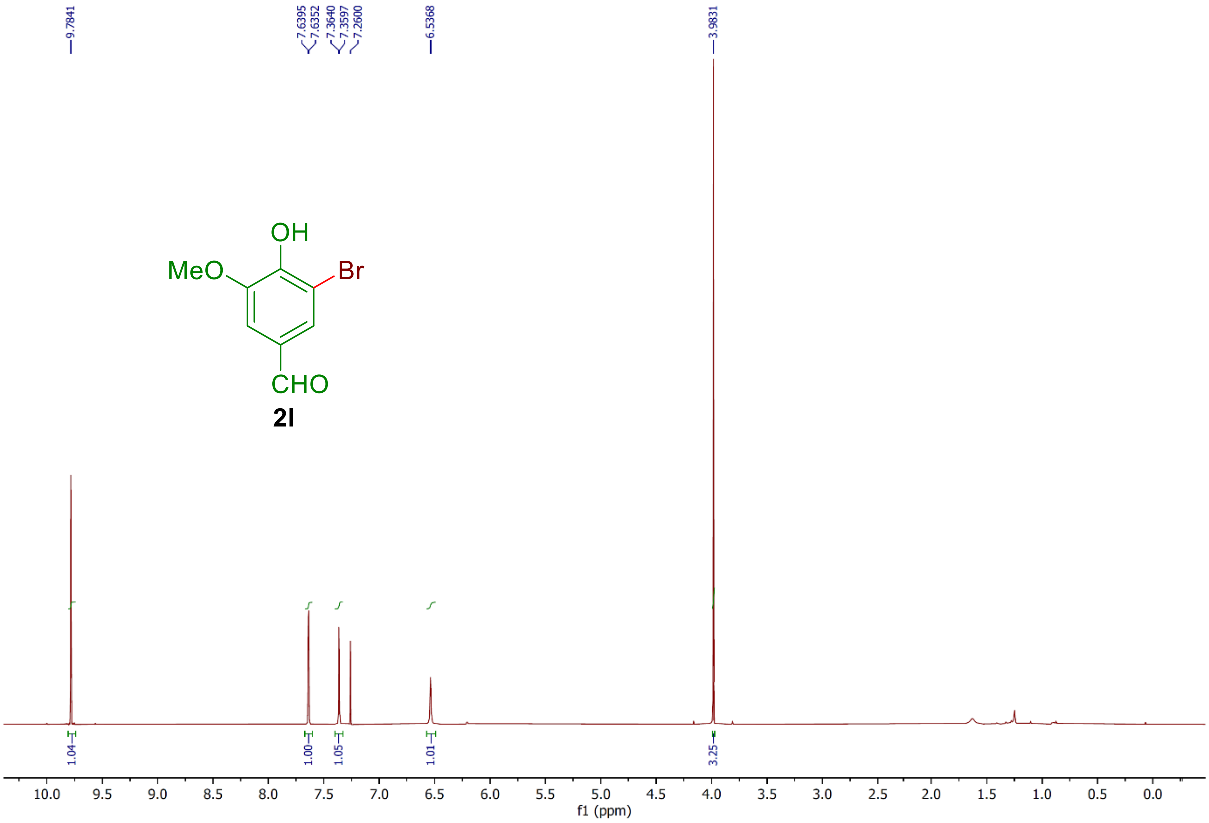

Figure S23: ^{1}H NMR spectrum of compound **2l**, ($CDCl_3$, 400 MHz).

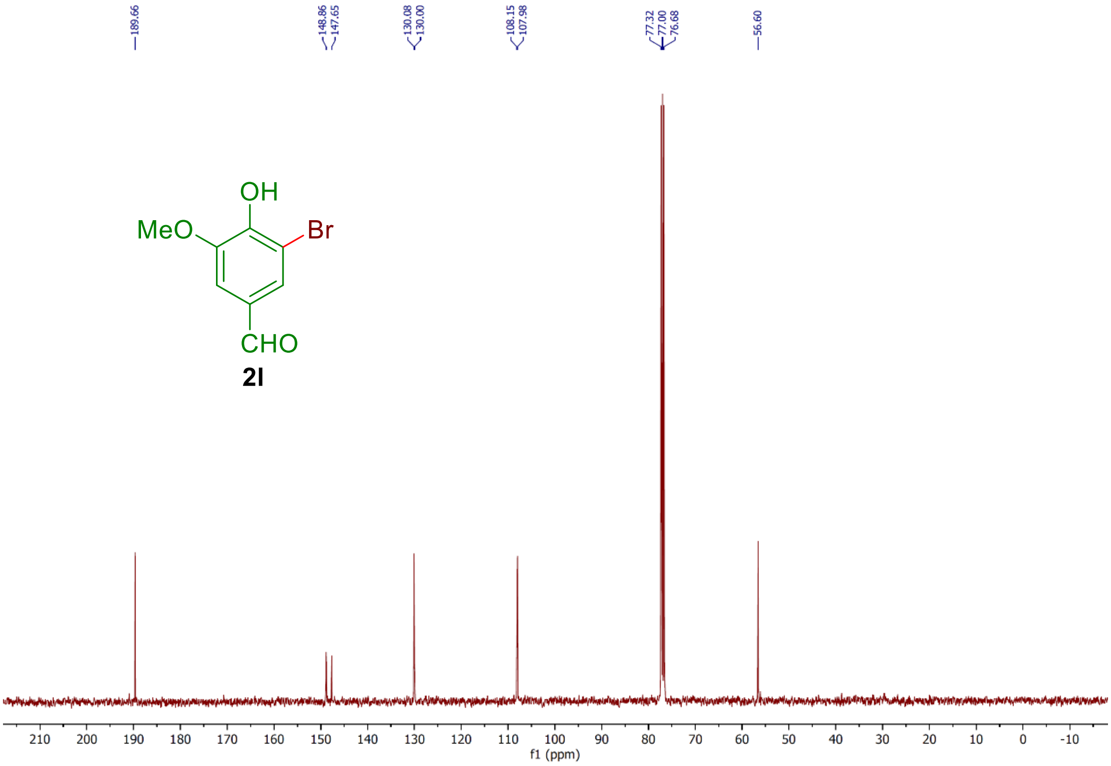

Figure S24: ^{13}C NMR spectrum of compound **2l**, ($CDCl_3$, 100 MHz).

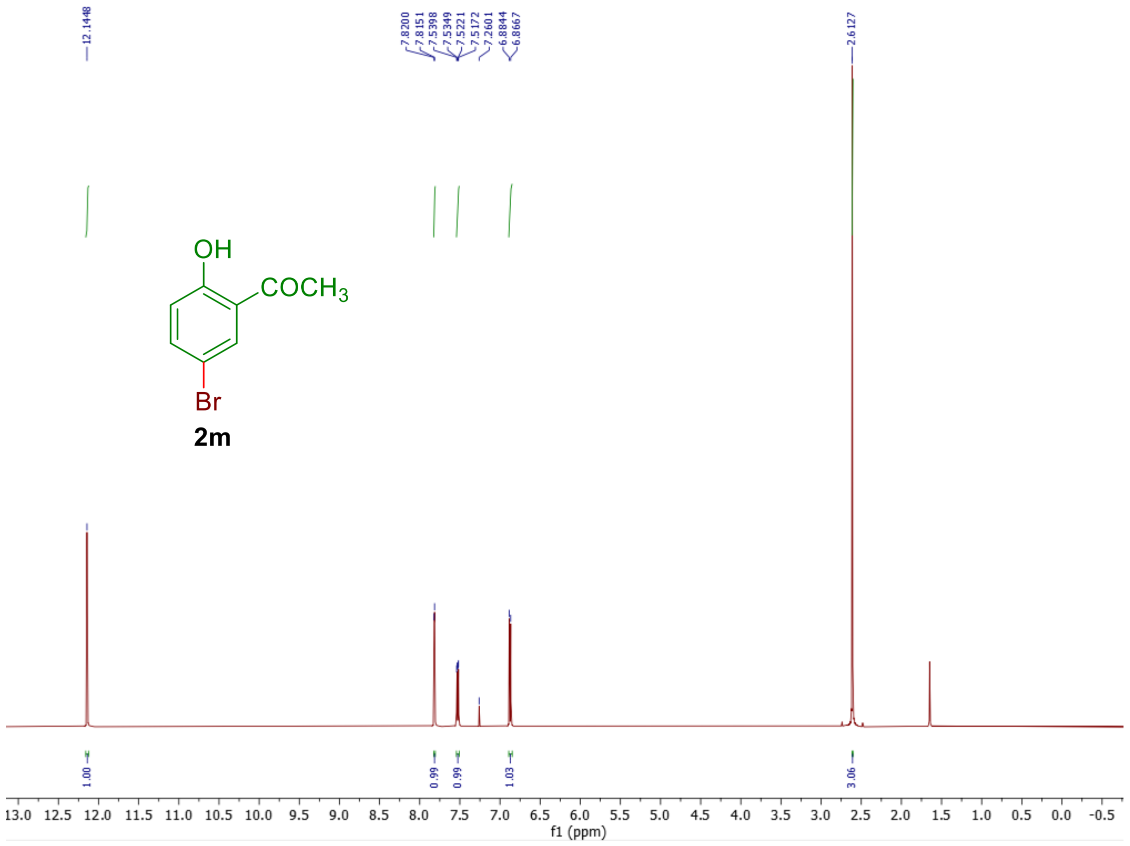

Figure S25: ^{1}H NMR spectrum of compound **2m**, ($CDCl_3$, 500 MHz).

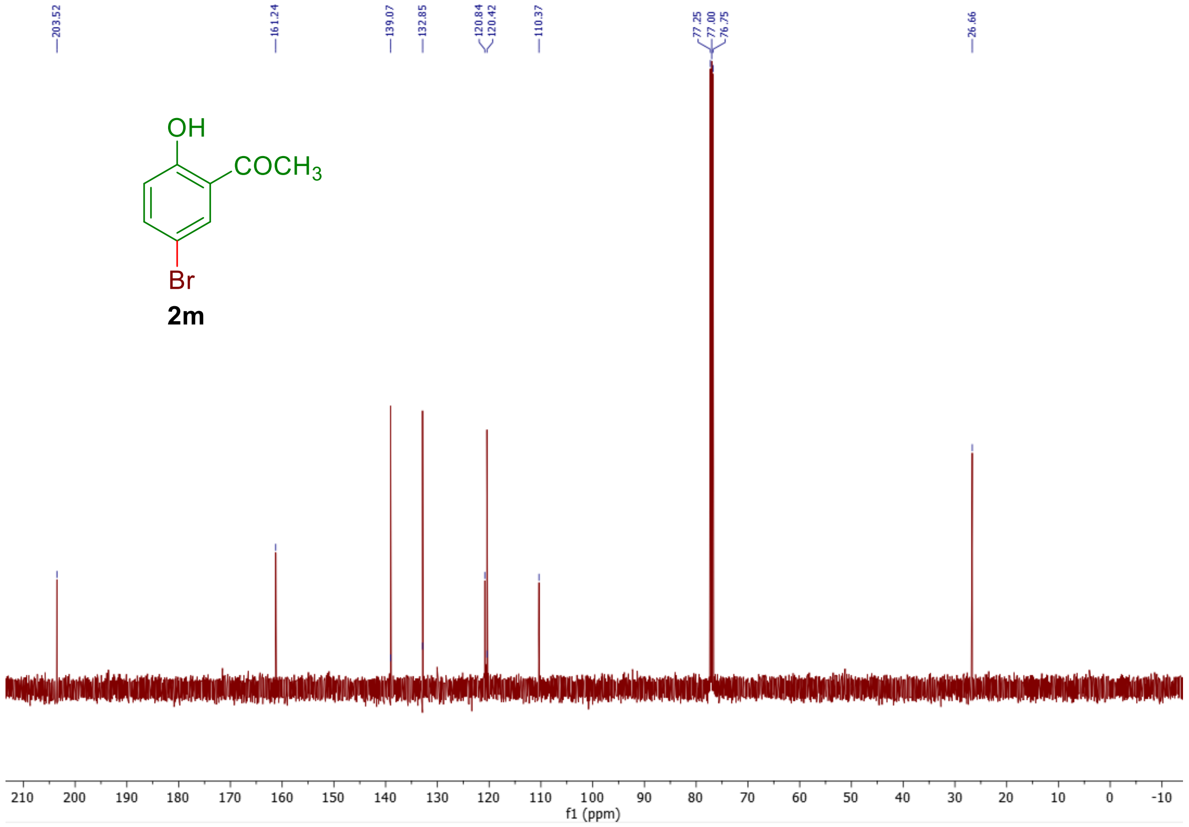

Figure S26: ^{13}C NMR spectrum of compound **2m**, ($CDCl_3$, 125 MHz).

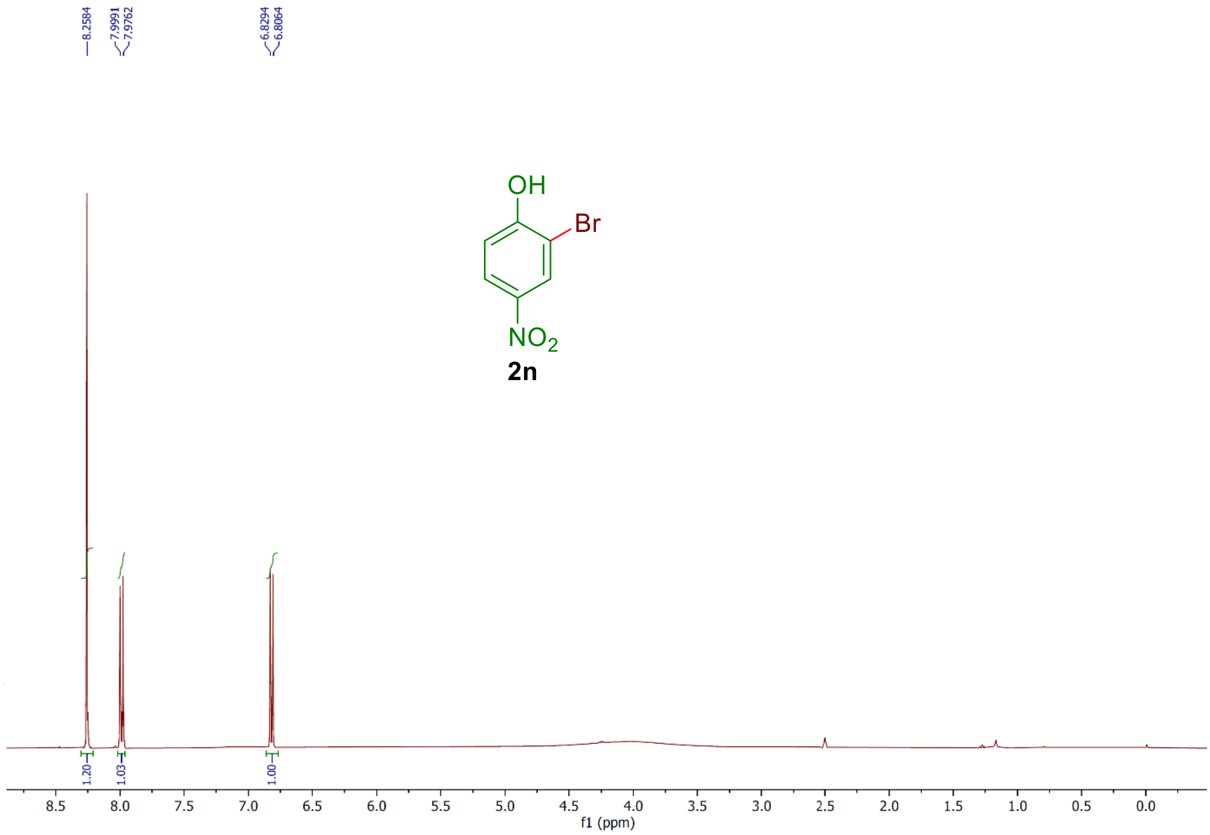

Figure S27: ^{1}H NMR spectrum of compound **2n**, ($CDCl_3$, 400 MHz).

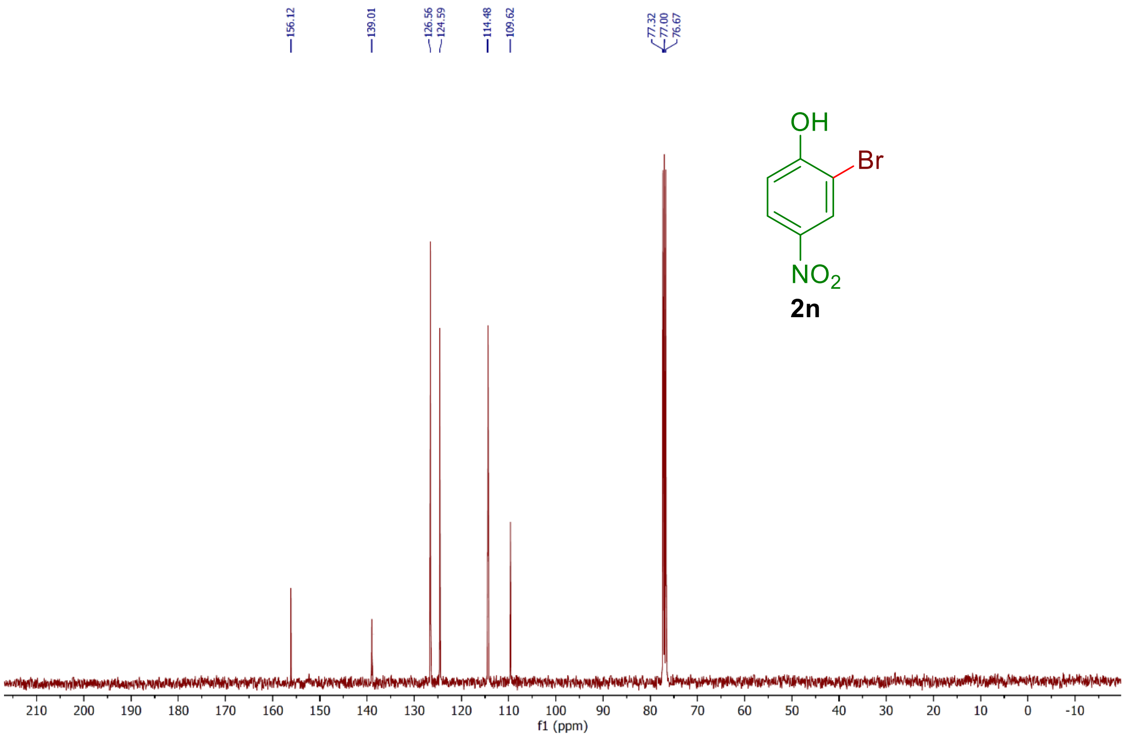

Figure S28: ^{13}C NMR spectrum of compound **2n**, ($CDCl_3$, 100 MHz).

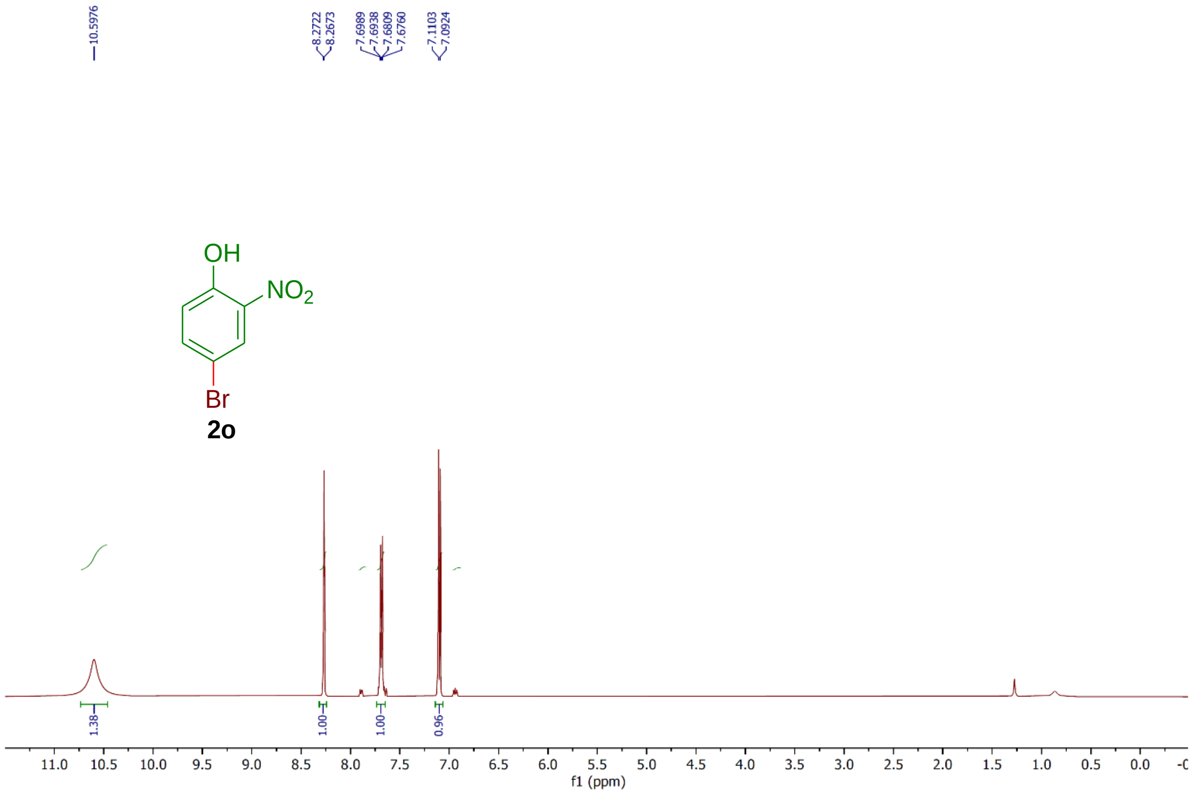

Figure S29: ^{1}H NMR spectrum of compound **2o**, ($CDCl_3$, 500 MHz).

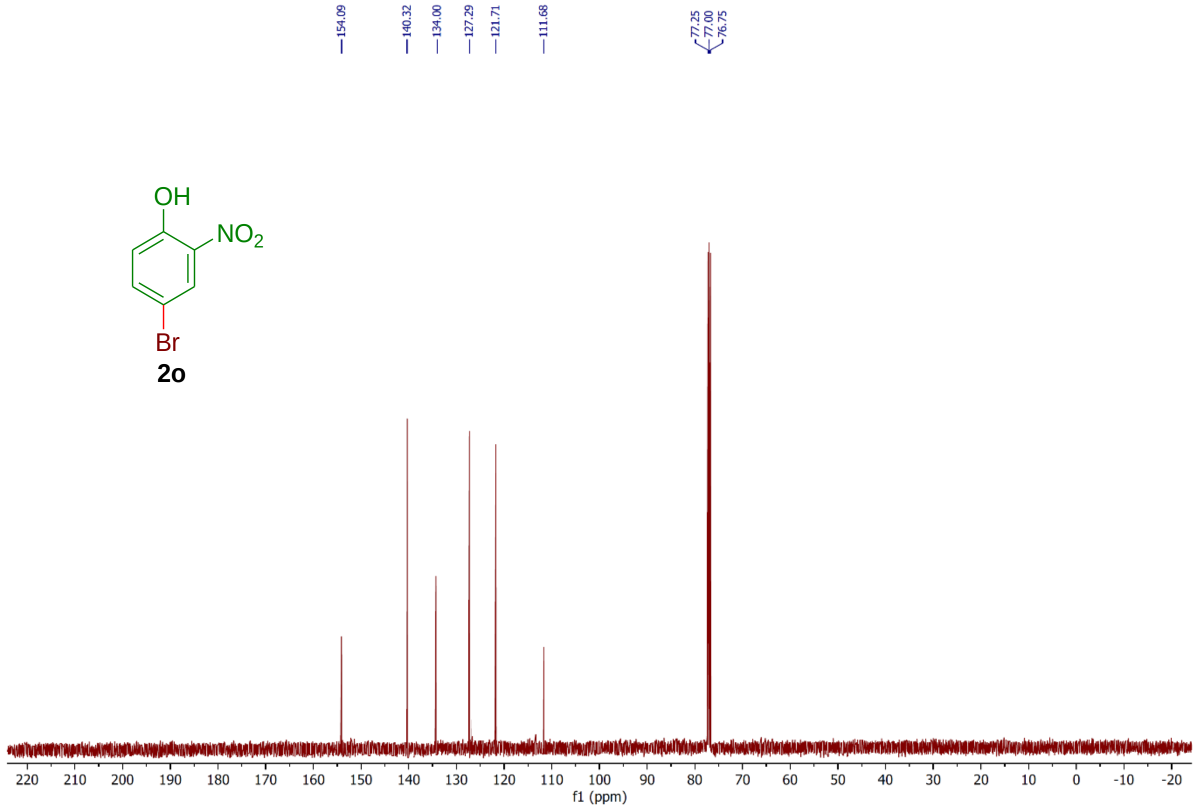

Figure S30: ^{13}C NMR spectrum of compound **2o**, ($CDCl_3$, 125 MHz).

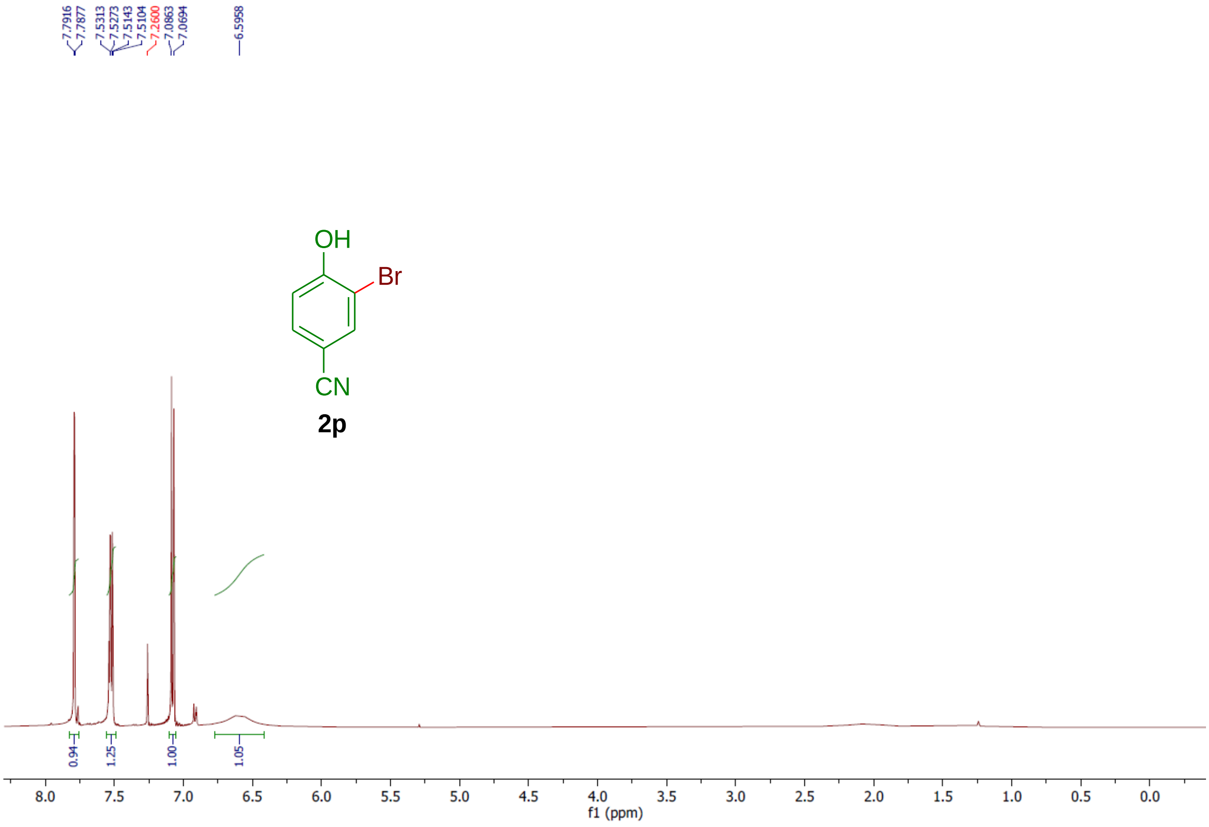

Figure S31: ^{1}H NMR spectrum of compound **2p**, ($CDCl_3$, 500 MHz).

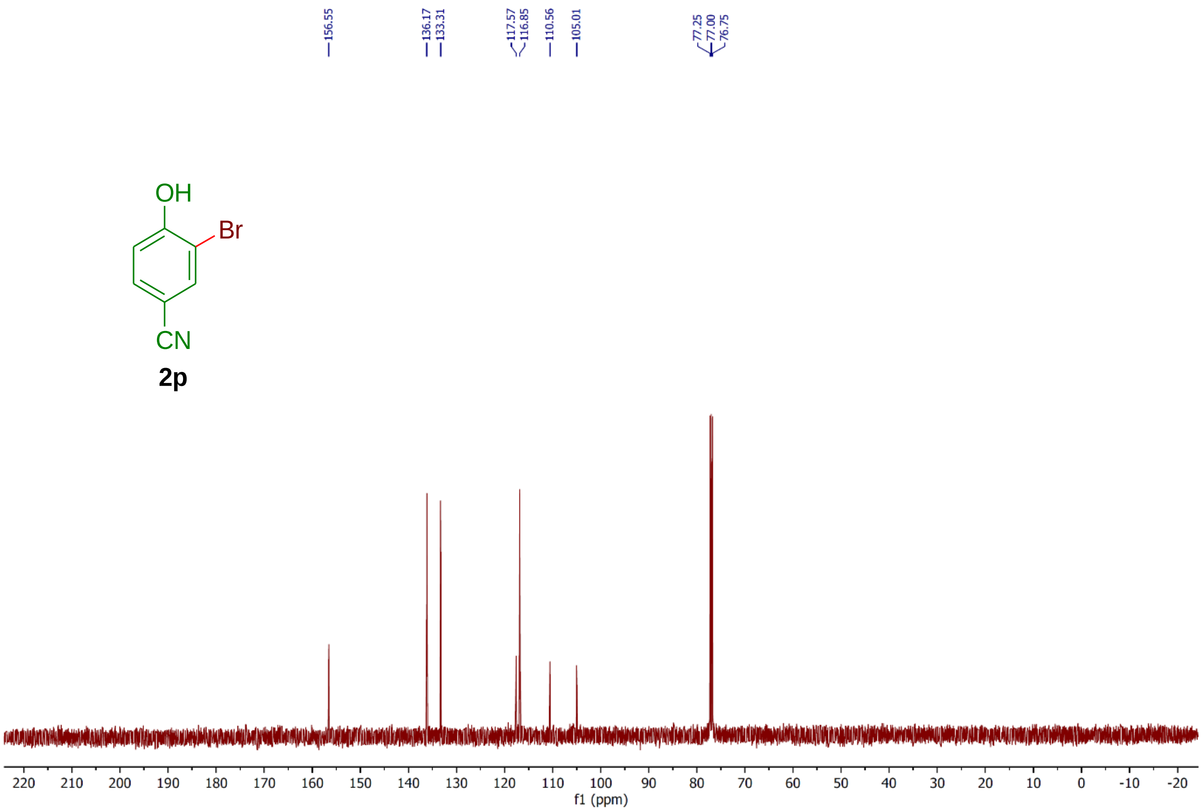

Figure S32: ^{13}C NMR spectrum of compound **2p**, ($CDCl_3$, 125 MHz).

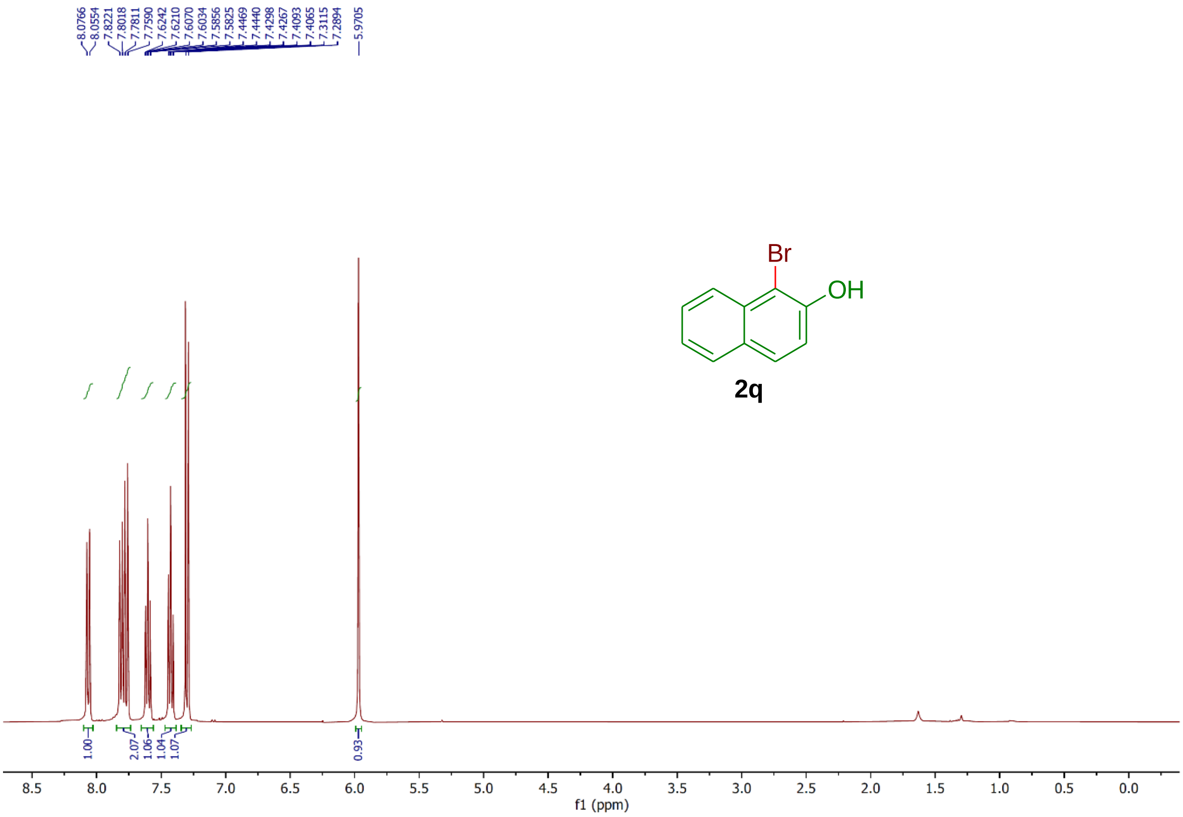

Figure S33: ^{1}H NMR spectrum of compound **2q**, ($CDCl_3$, 400 MHz).

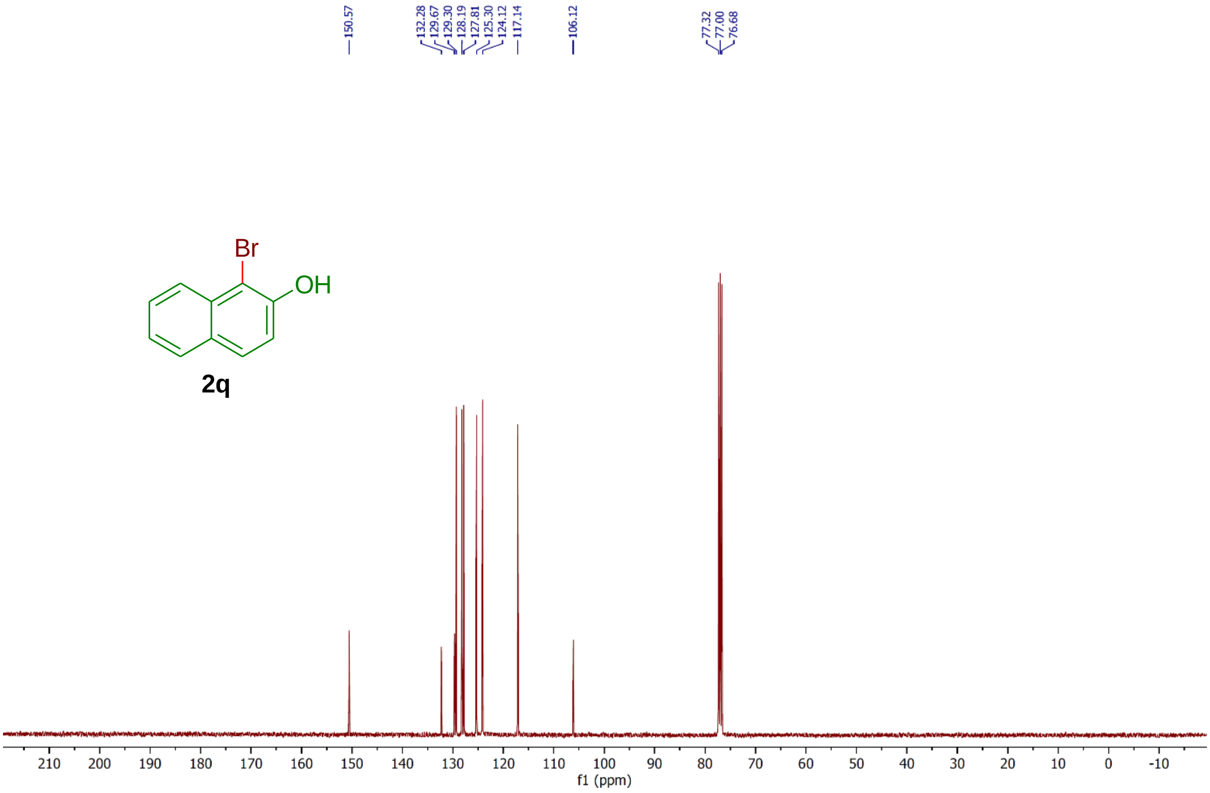

Figure S34: ^{13}C NMR spectrum of compound **2q**, ($CDCl_3$, 100 MHz).

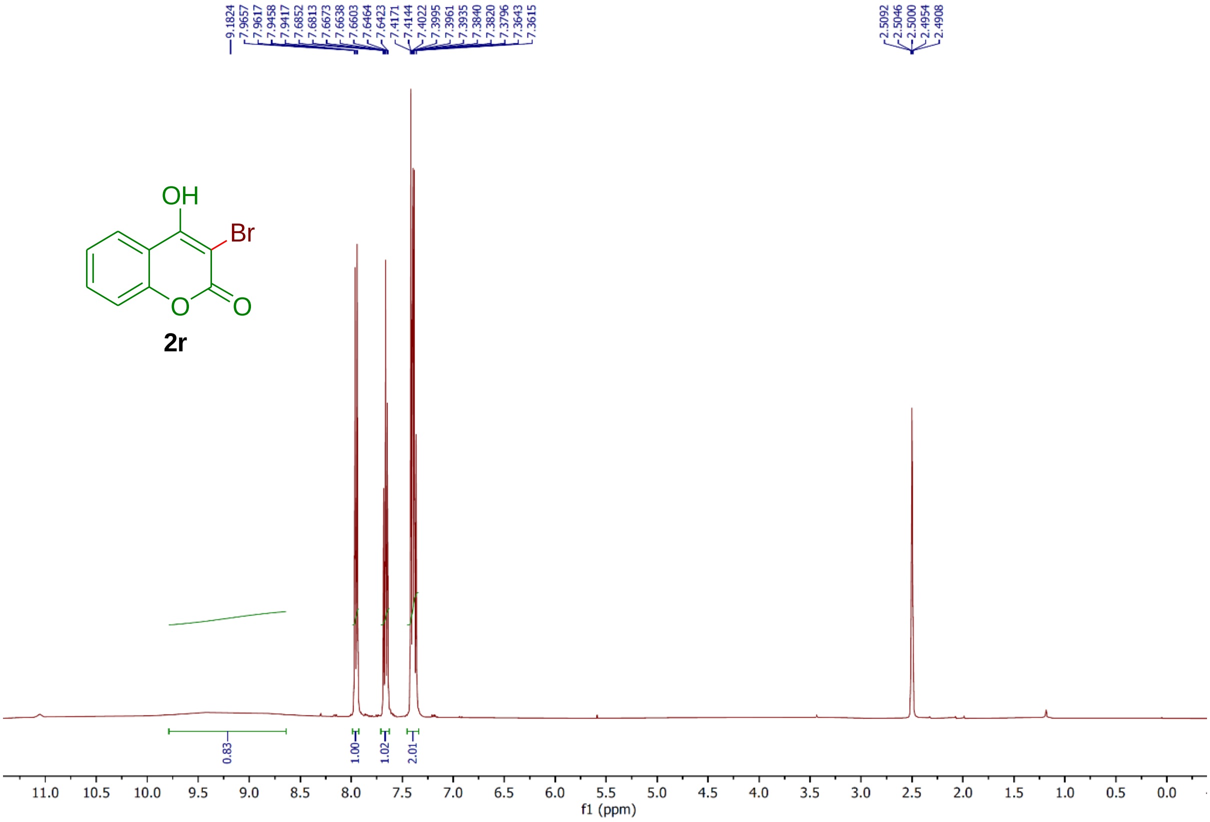

Figure S35: ^{1}H NMR spectrum of compound **2r**, ($CDCl_3$, 400 MHz).

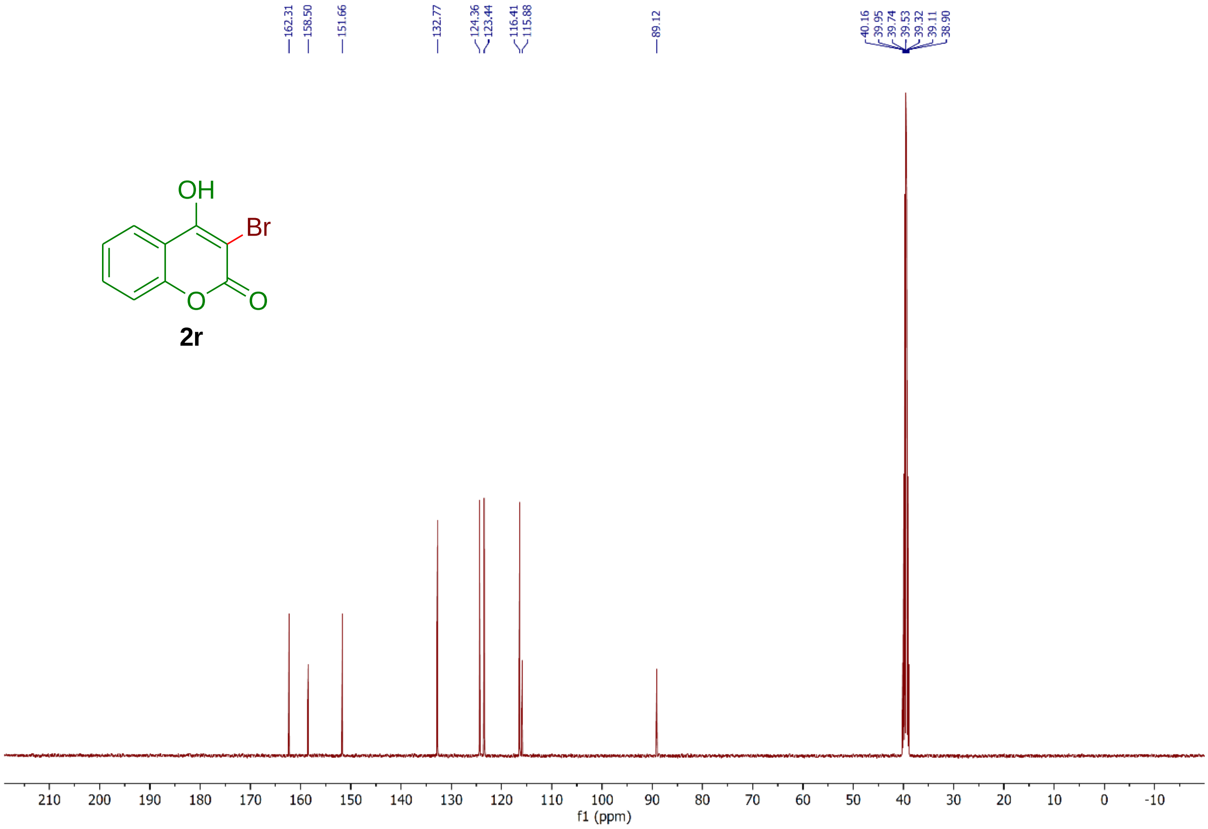

Figure S36: ^{13}C NMR spectrum of compound **2r**, ($CDCl_3$, 100 MHz).

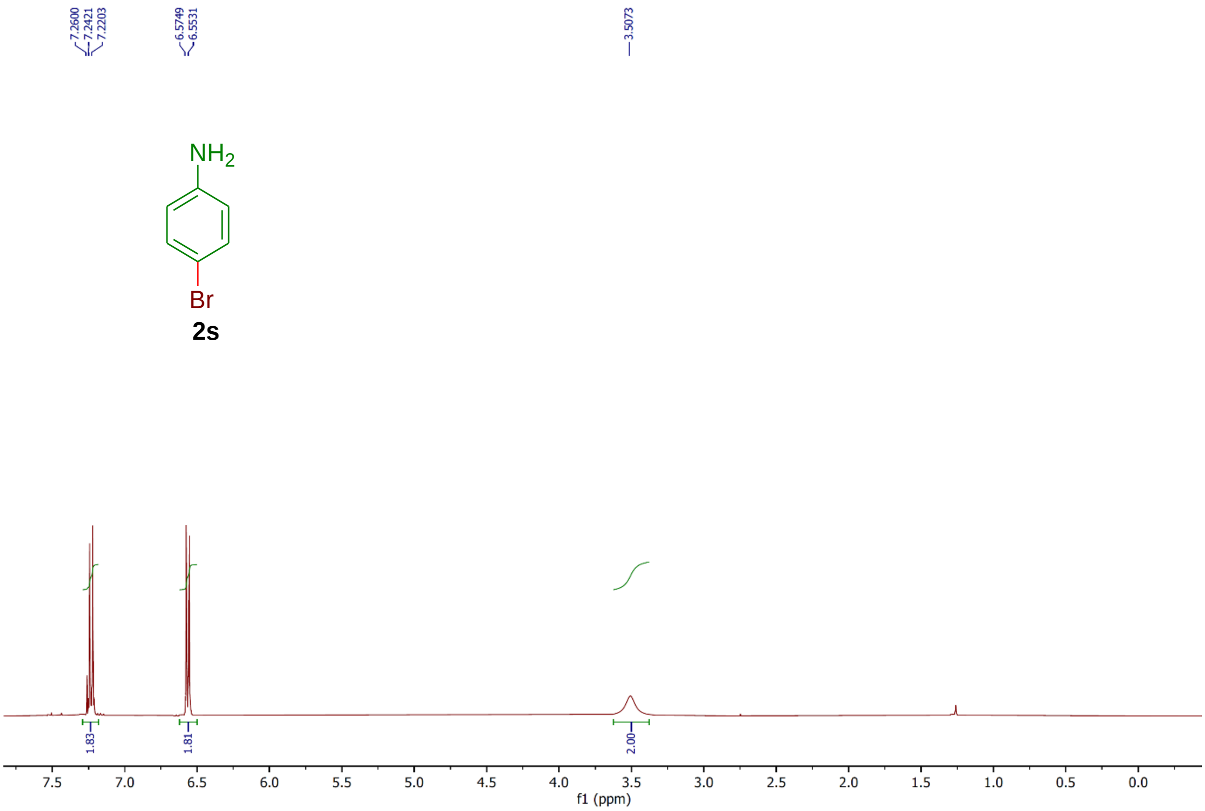

Figure S37: ^{1}H NMR spectrum of compound **2s**, ($CDCl_3$, 400 MHz).

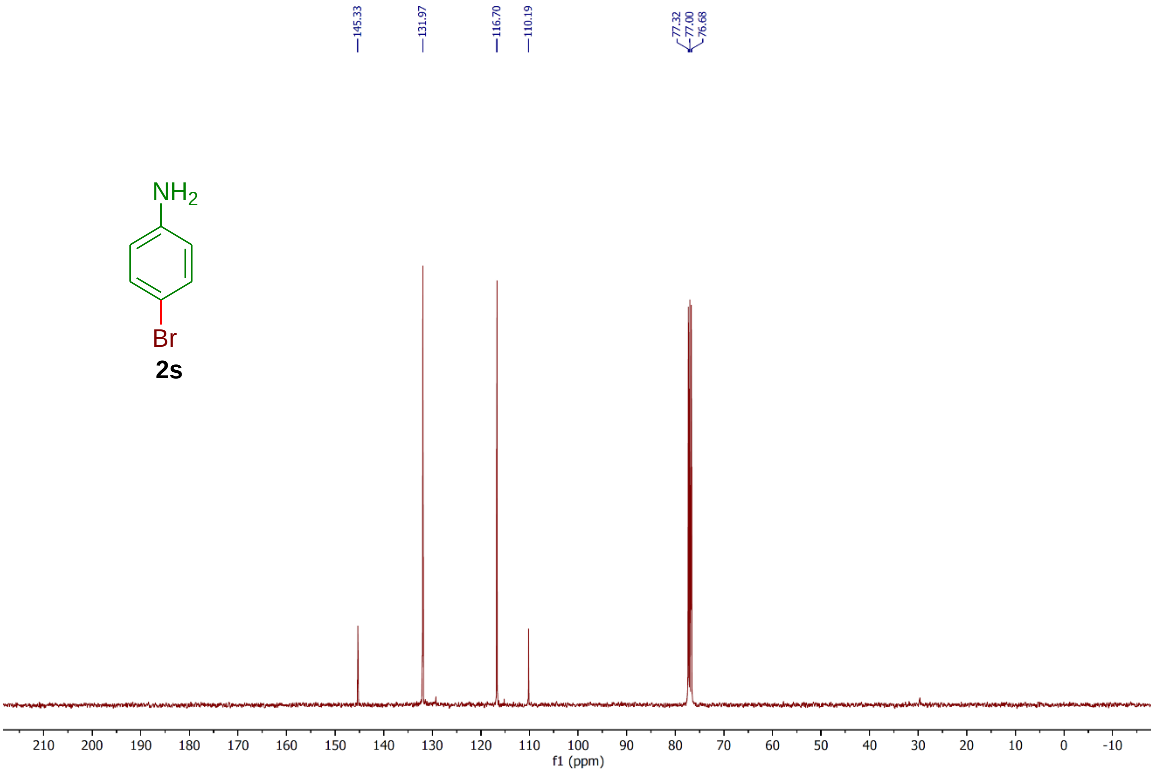

Figure S38: ^{13}C NMR spectrum of compound **2s**, ($CDCl_3$, 100 MHz).

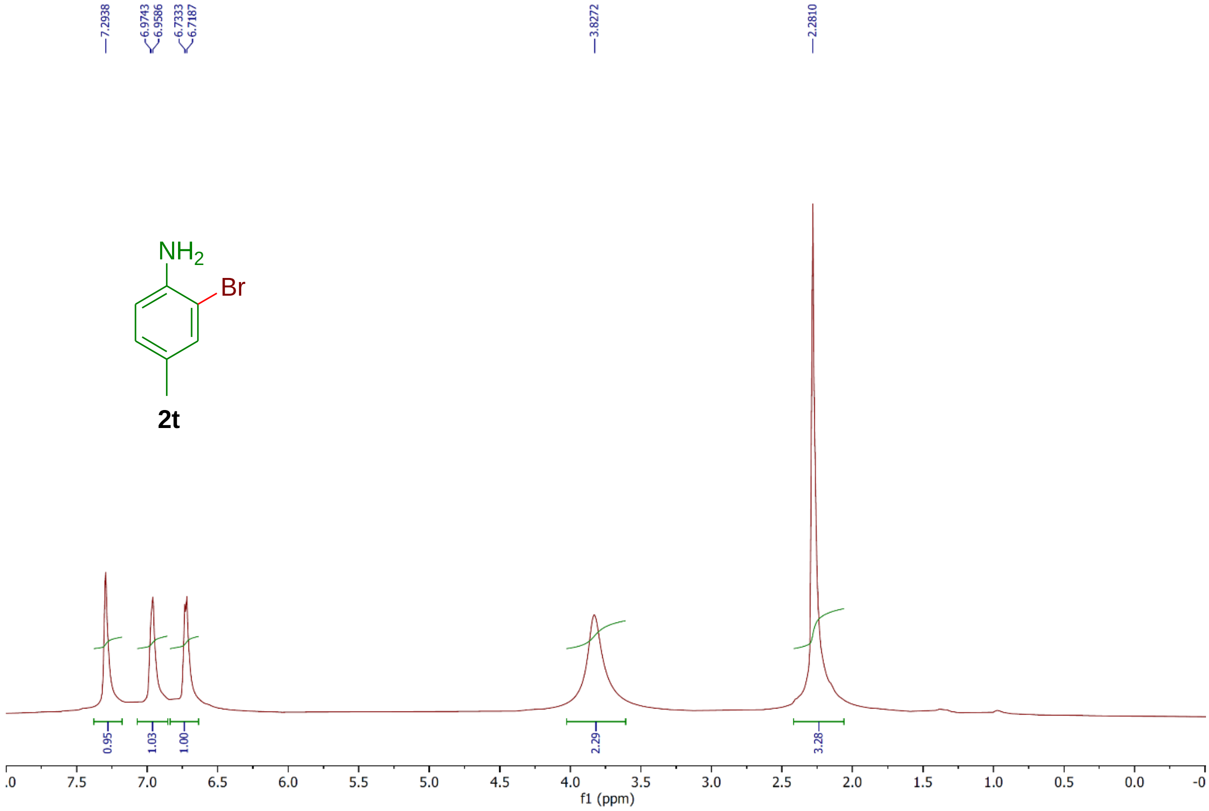

Figure S39: ^{1}H NMR spectrum of compound **2t**, ($CDCl_3$, 500 MHz).

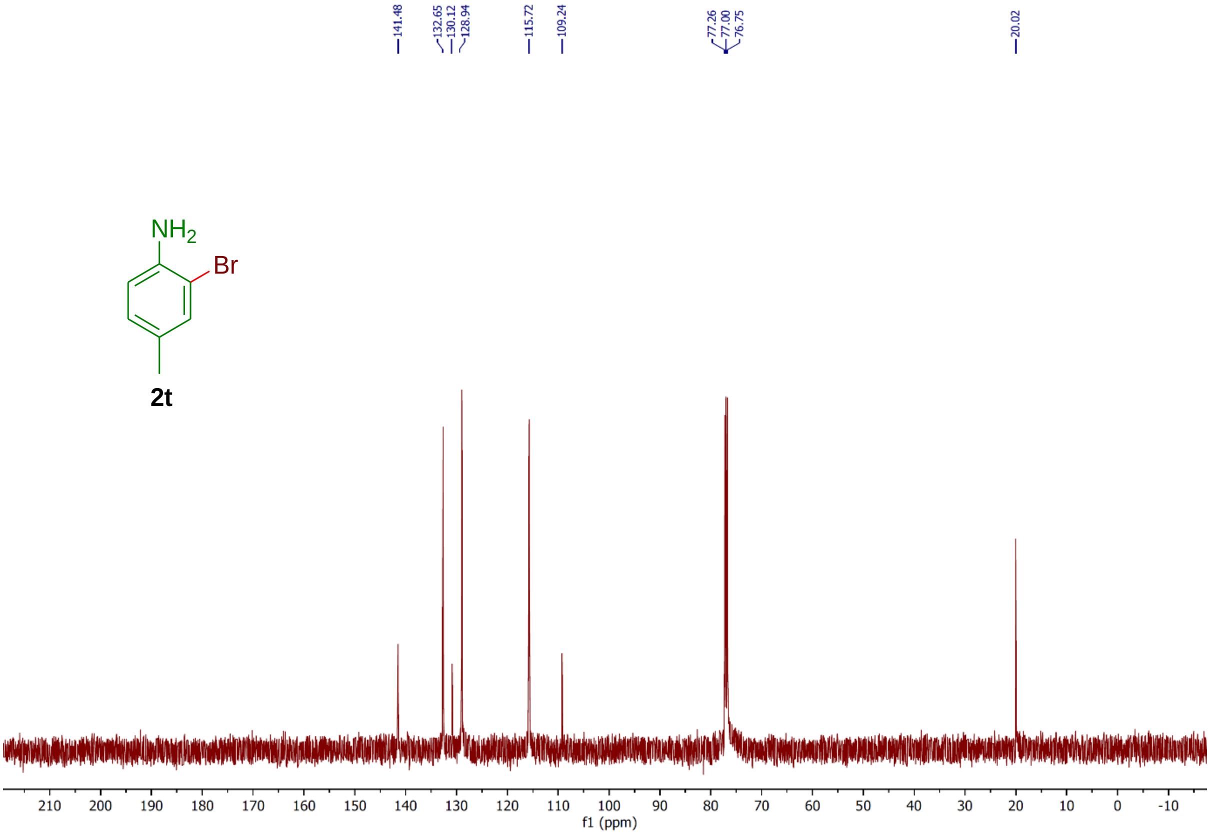

Figure S40: ^{13}C NMR spectrum of compound **2t**, ($CDCl_3$, 125 MHz).

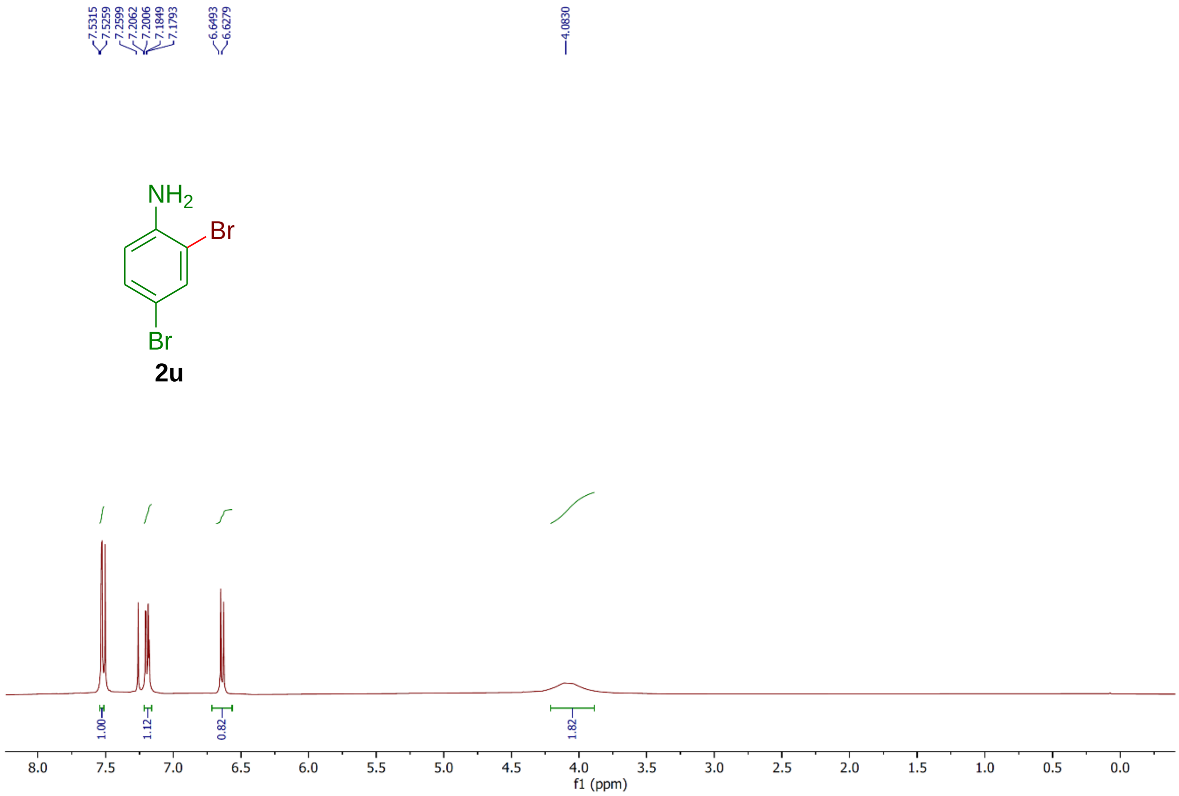

Figure S41: ^{1}H NMR spectrum of compound **2u**, ($CDCl_3$, 400 MHz).

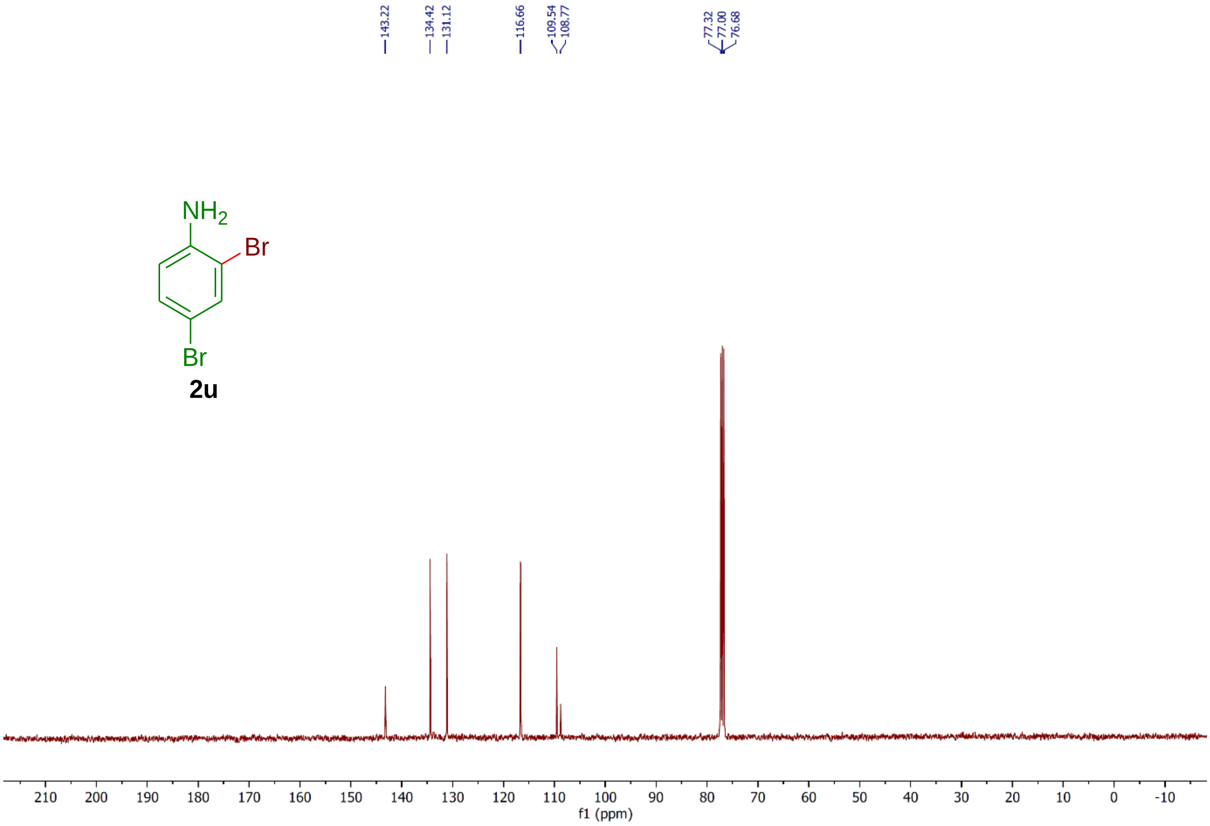

Figure S42: ^{13}C NMR spectrum of compound **2u**, ($CDCl_3$, 100 MHz).

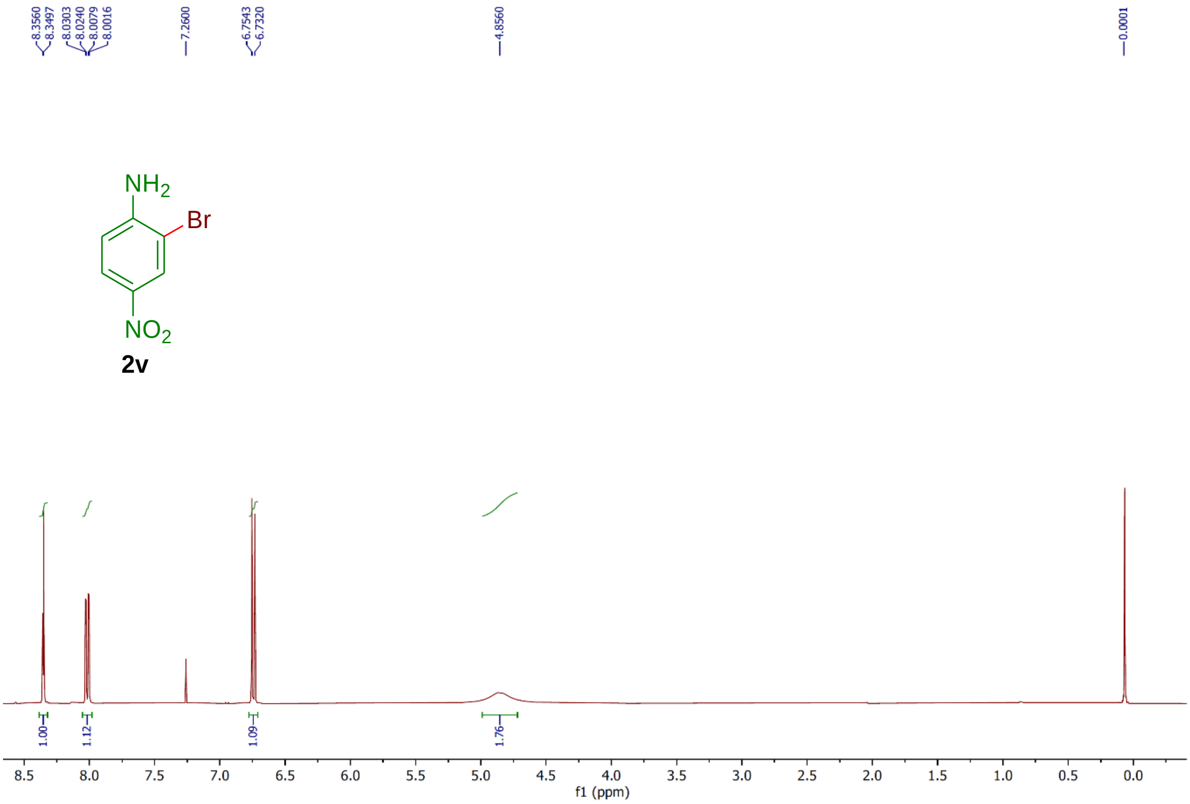

Figure S43: ^{1}H NMR spectrum of compound **2v**, ($CDCl_3$, 400 MHz).

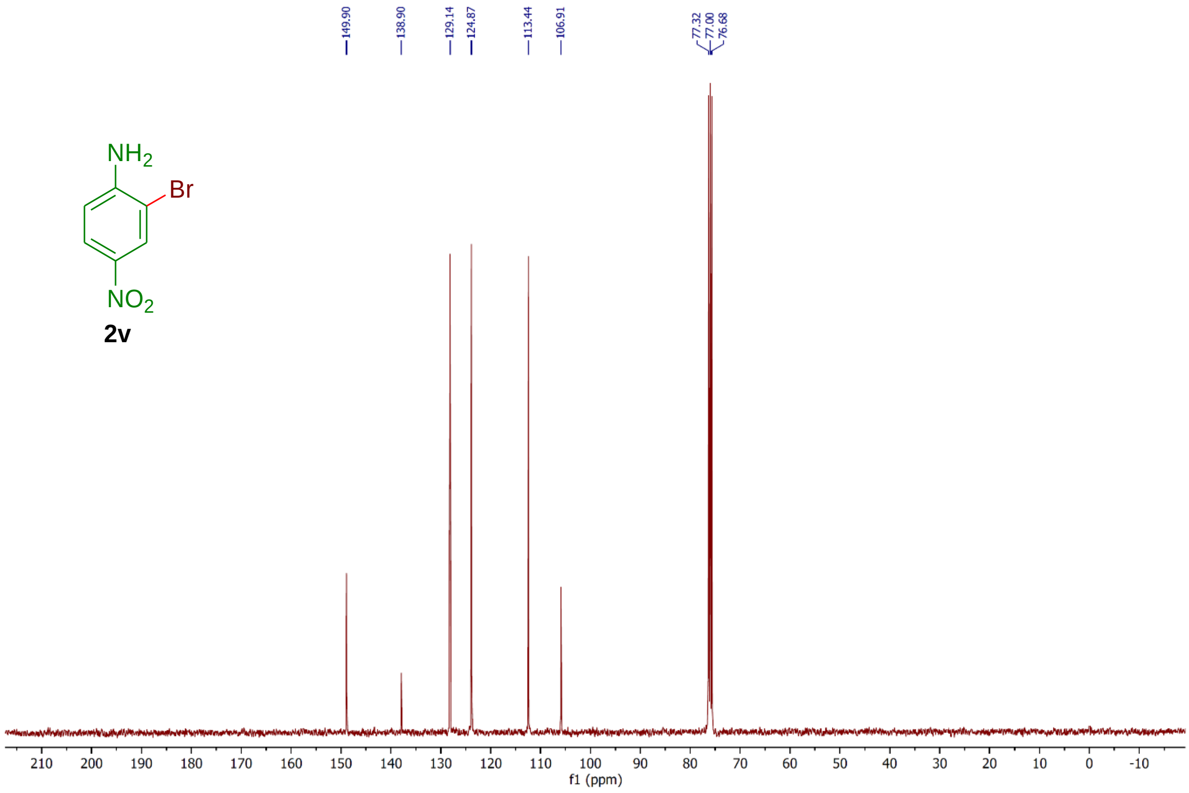

Figure S44: ^{13}C NMR spectrum of compound **2v**, ($CDCl_3$, 100 MHz).

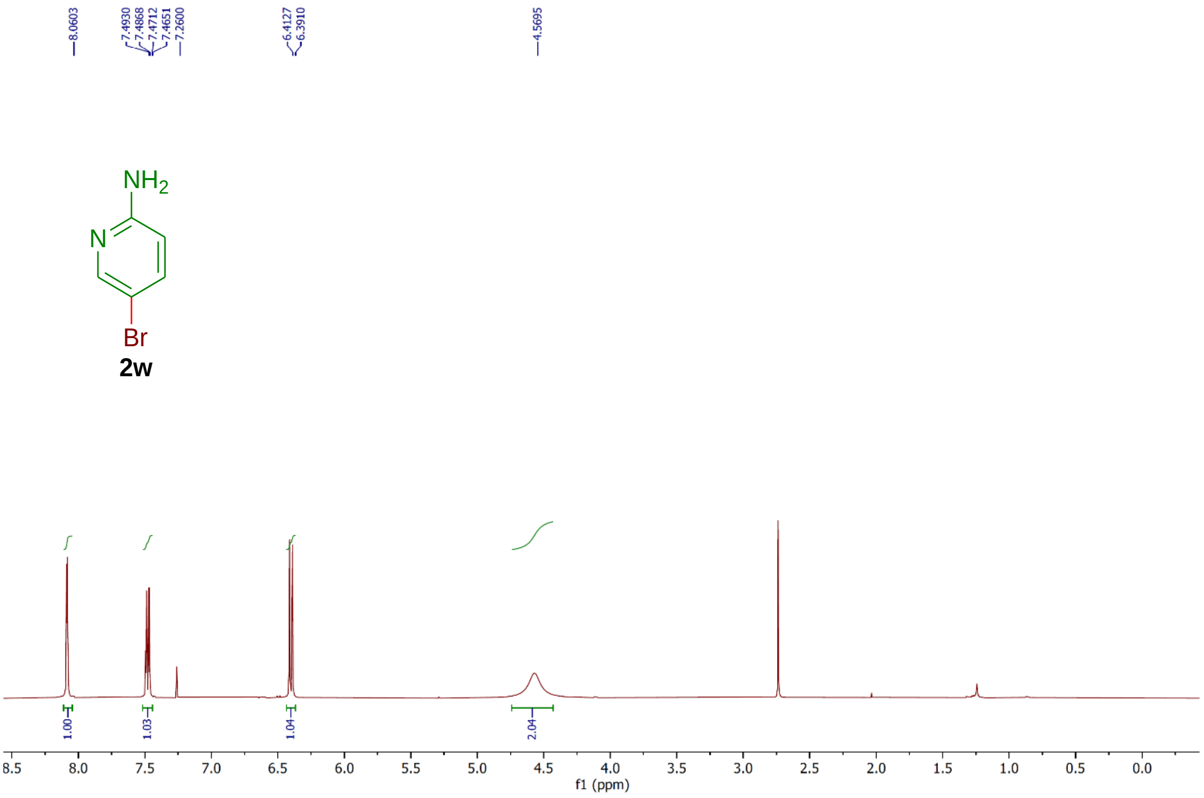

Figure S45: ^{1}H NMR spectrum of compound **2w**, ($CDCl_3$, 400 MHz).

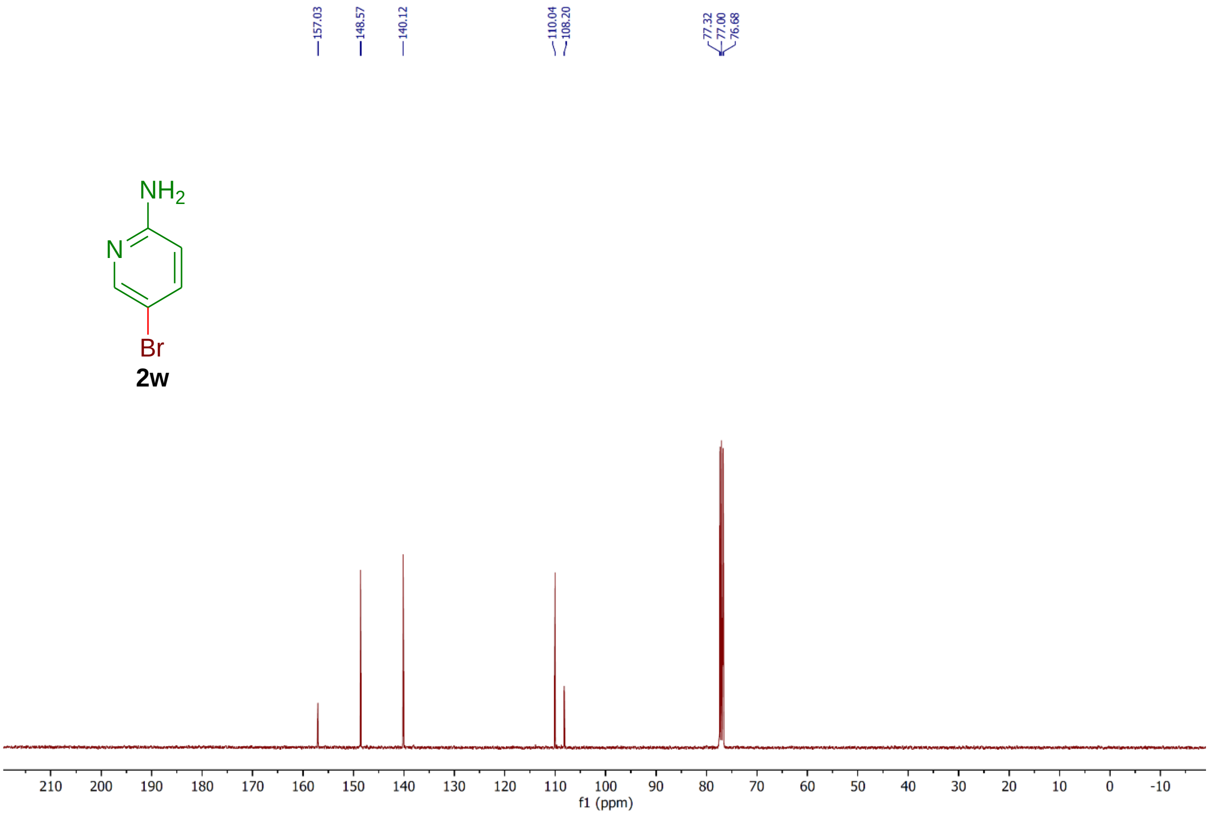

Figure S46: ^{13}C NMR spectrum of compound **2w**, ($CDCl_3$, 100 MHz).

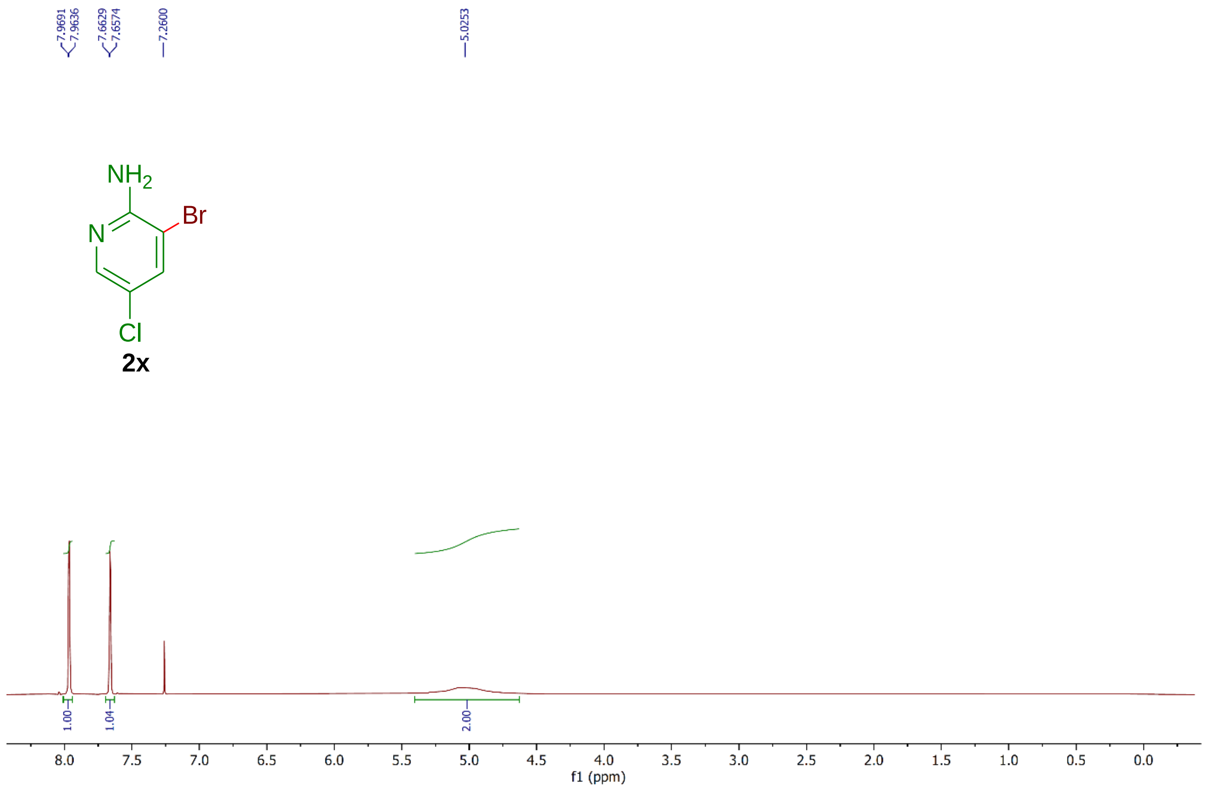

Figure S47: ^{1}H NMR spectrum of compound **2x**, ($CDCl_3$, 400 MHz).

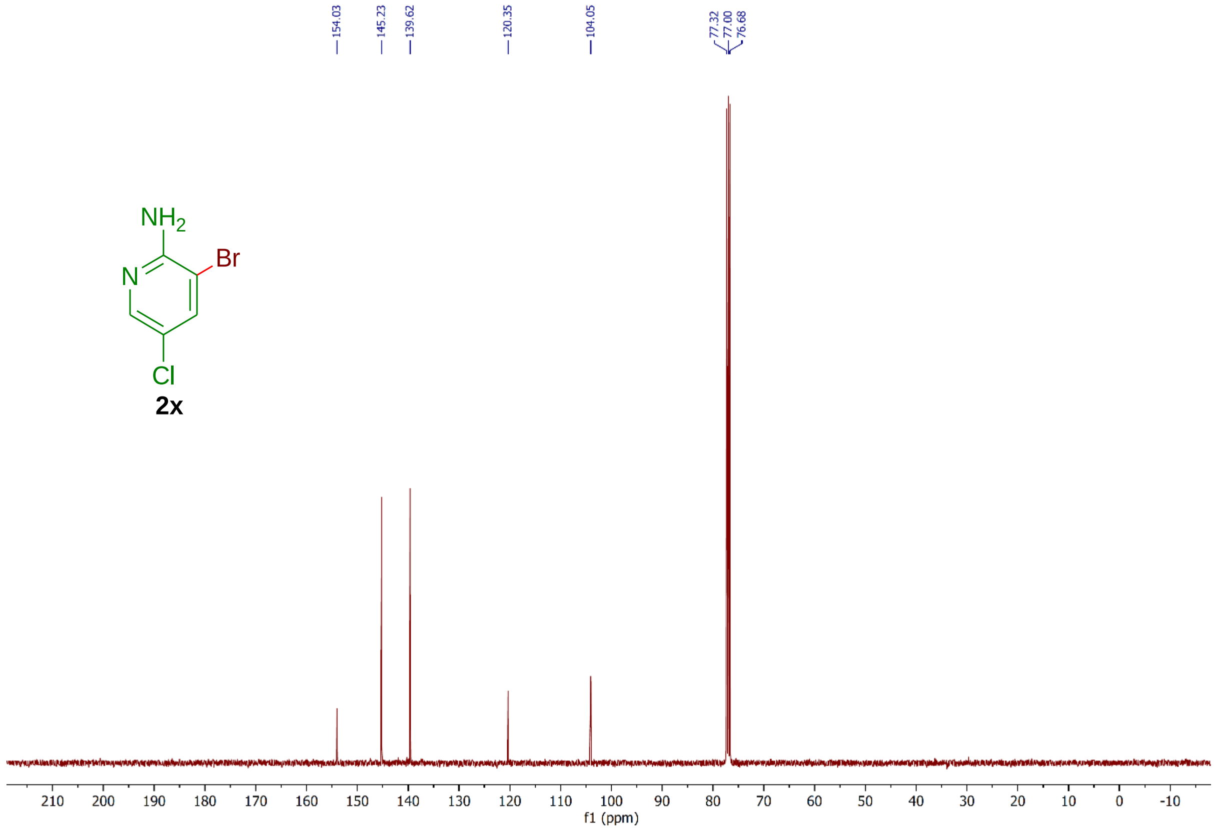

Figure S48: ^{13}C NMR spectrum of compound **2x**, ($CDCl_3$, 100 MHz).

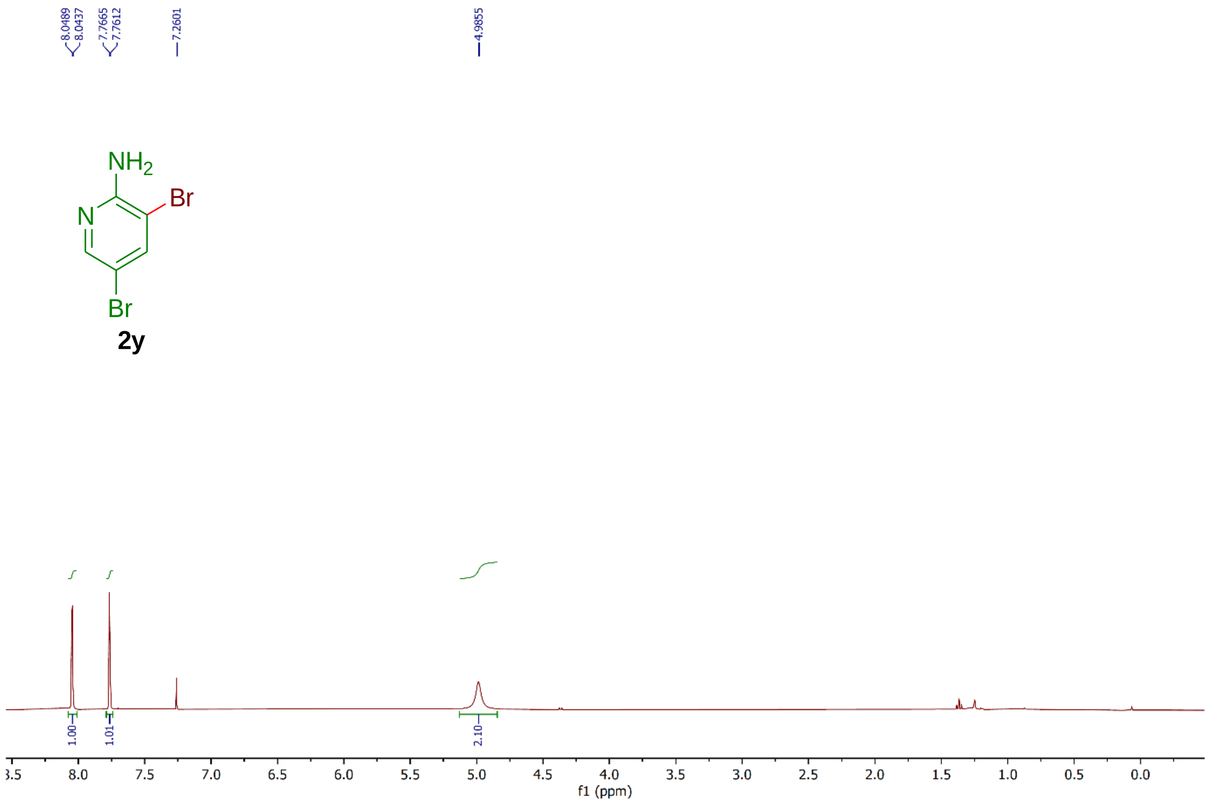

Figure S49: ^{1}H NMR spectrum of compound **2y**, ($CDCl_3$, 400 MHz).

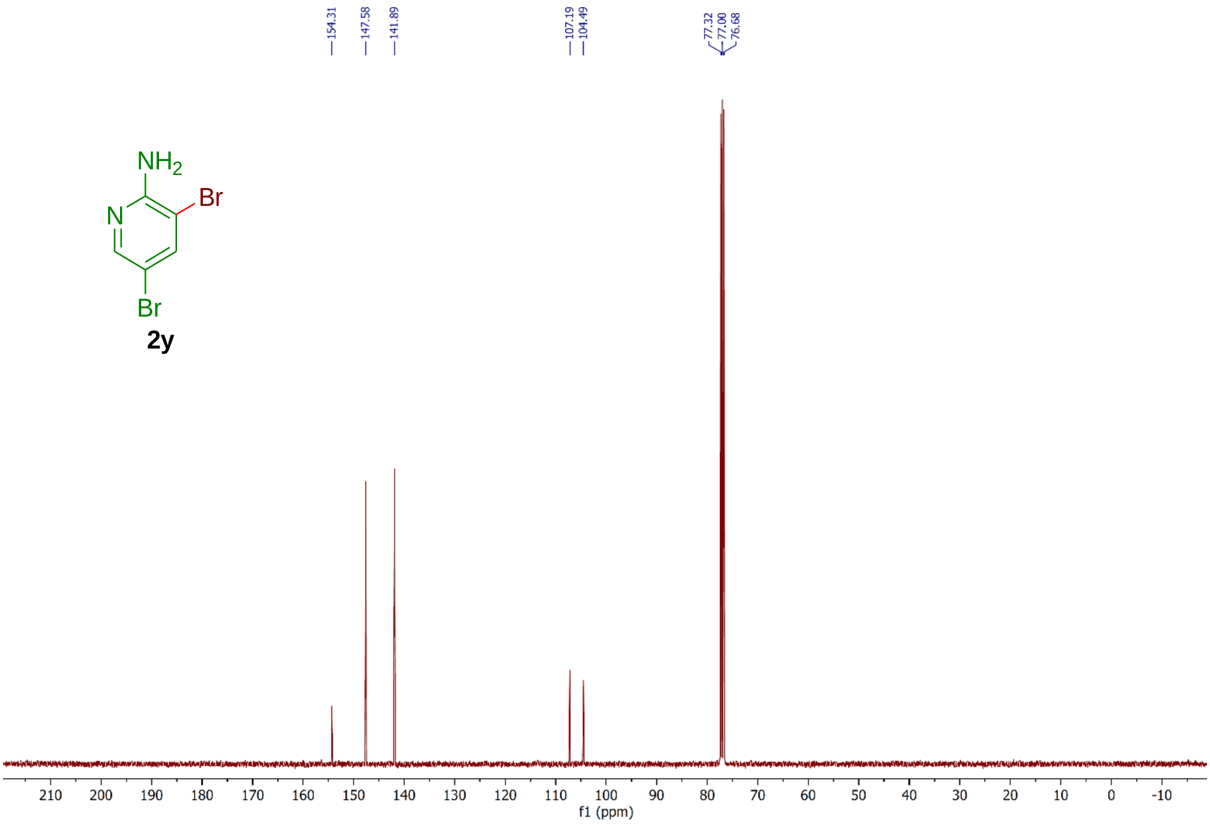

Figure S50: ^{13}C NMR spectrum of compound **2y**, ($CDCl_3$, 100 MHz).

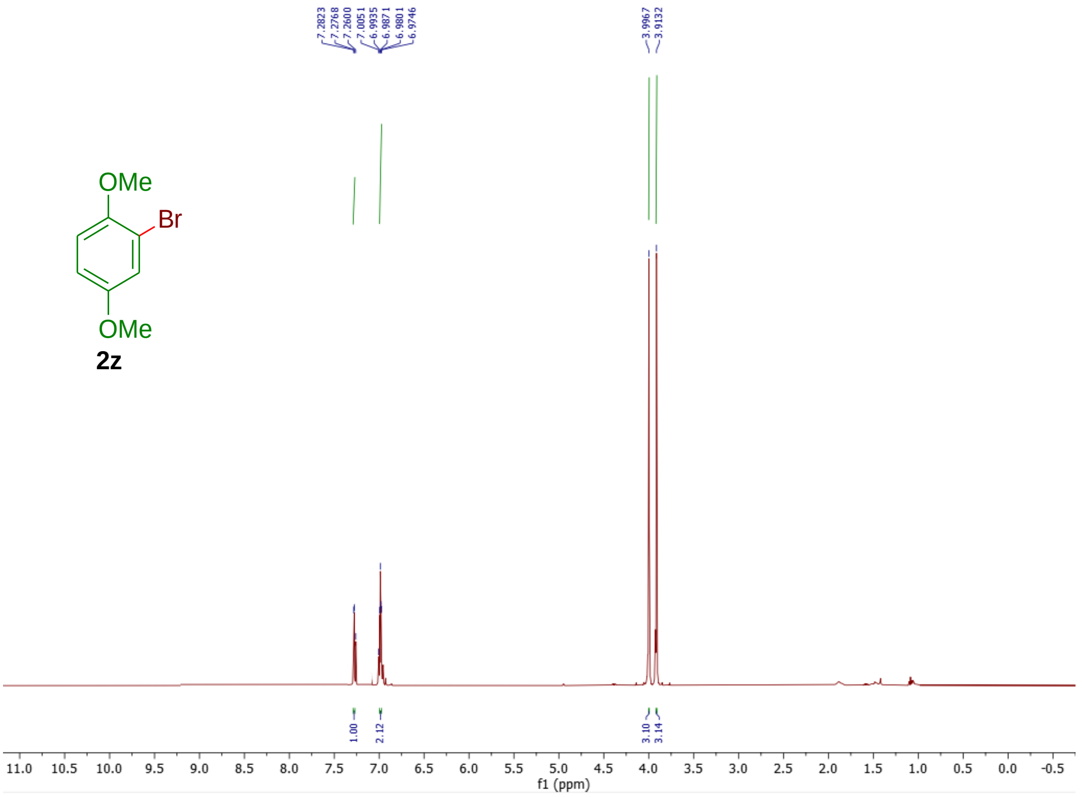

Figure S51: ^{1}H NMR spectrum of compound **2z**, ($CDCl_3$, 500 MHz).

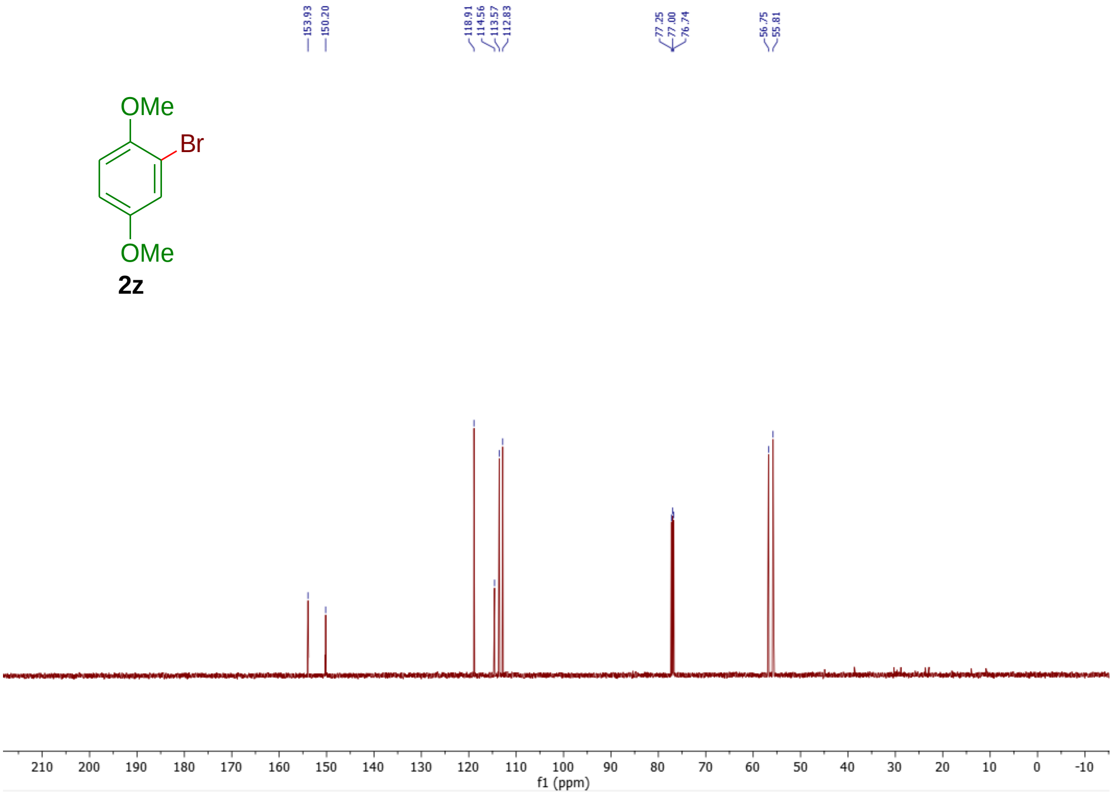

Figure S52: ^{13}C NMR spectrum of compound **2z**, ($CDCl_3$, 125 MHz).

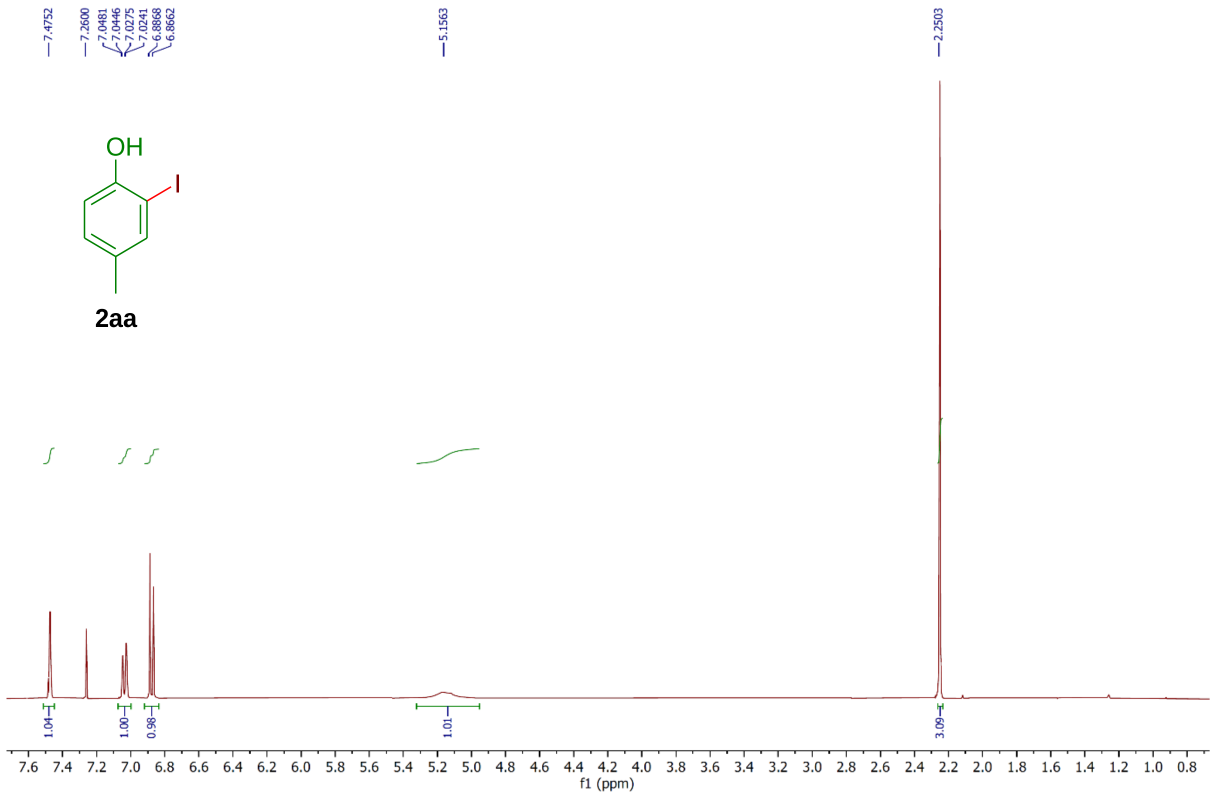

Figure S53: ^{1}H NMR spectrum of compound **2aa**, ($CDCl_3$, 400 MHz).

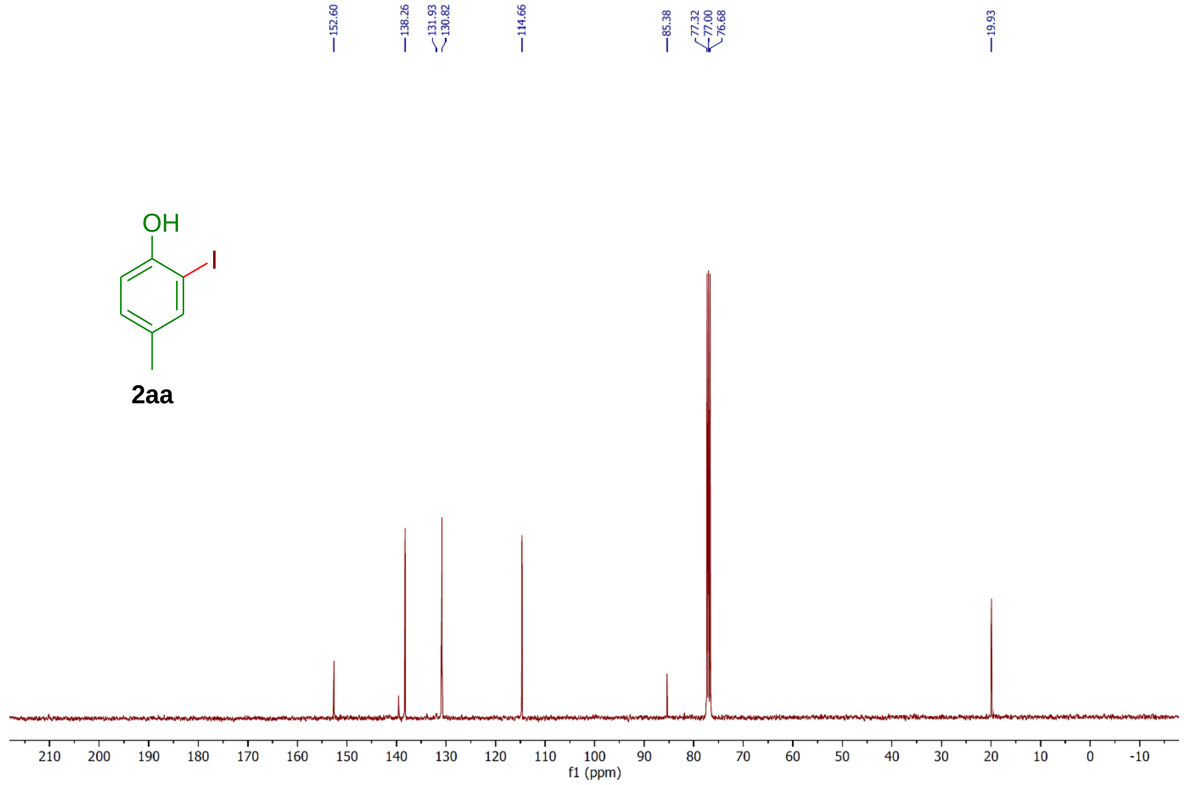

Figure S54: ^{13}C NMR spectrum of compound **2aa**, ($CDCl_3$, 100 MHz).

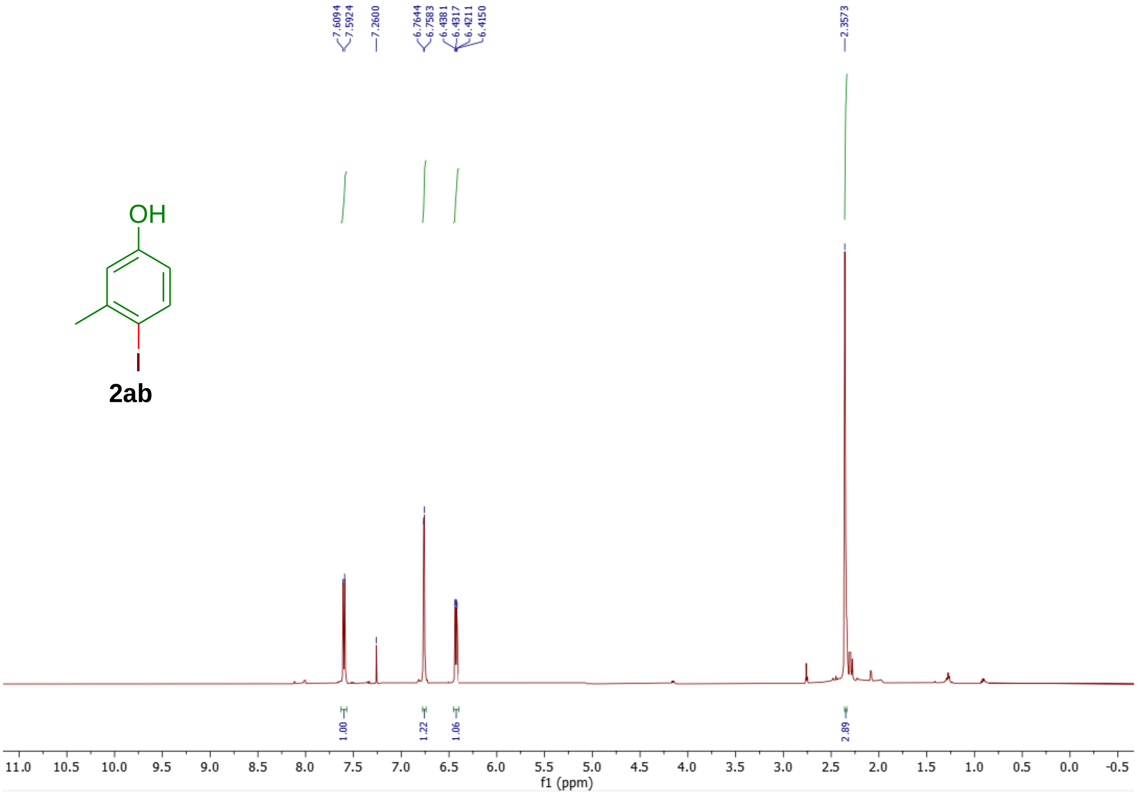

Figure S55: ^{1}H NMR spectrum of compound **2ab**, ($CDCl_3$, 500 MHz).

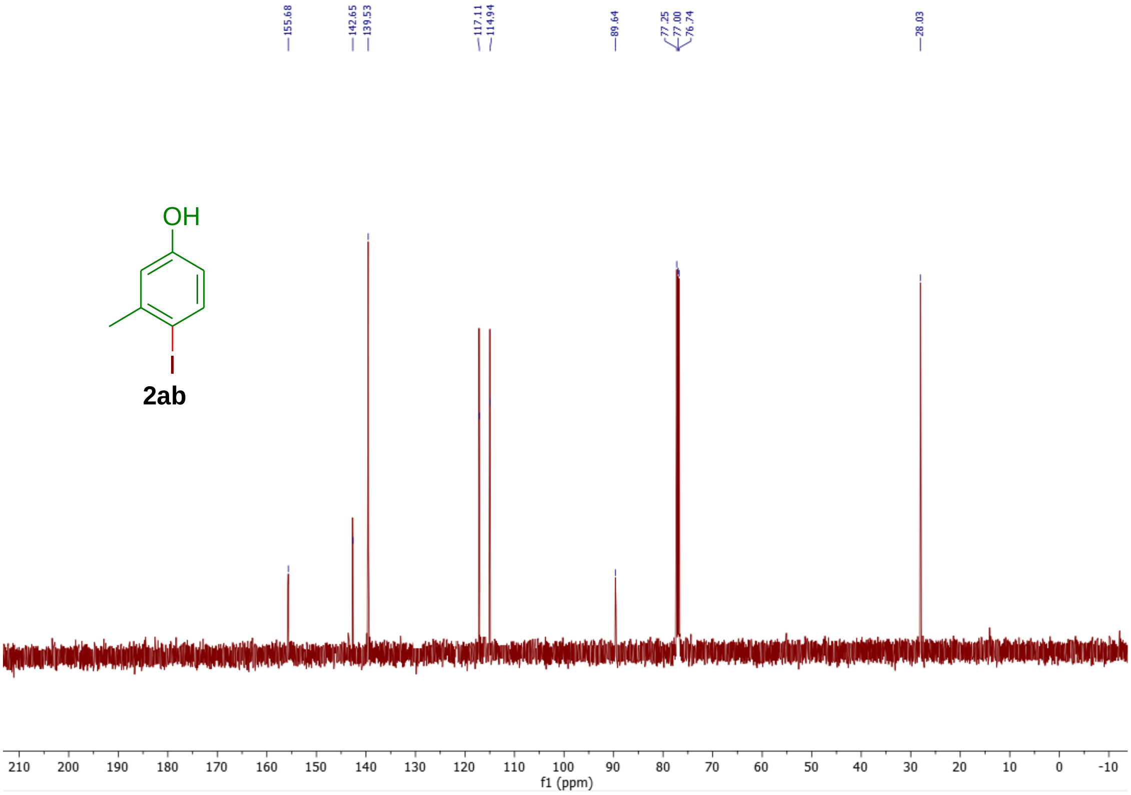

Figure S56: ^{13}C NMR spectrum of compound **2ab**, ($CDCl_3$, 125 MHz).

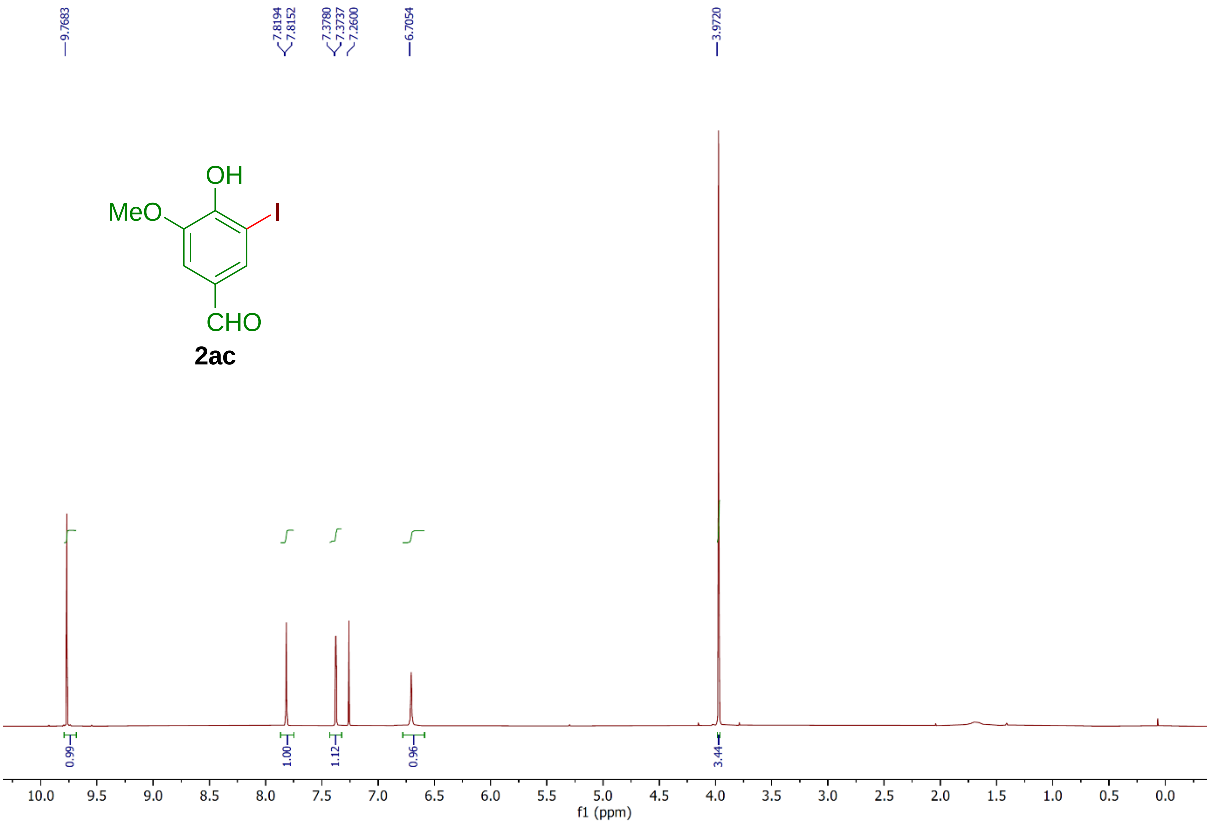

Figure S57: ^{1}H NMR spectrum of compound **2ac**, ($CDCl_3$, 400 MHz).

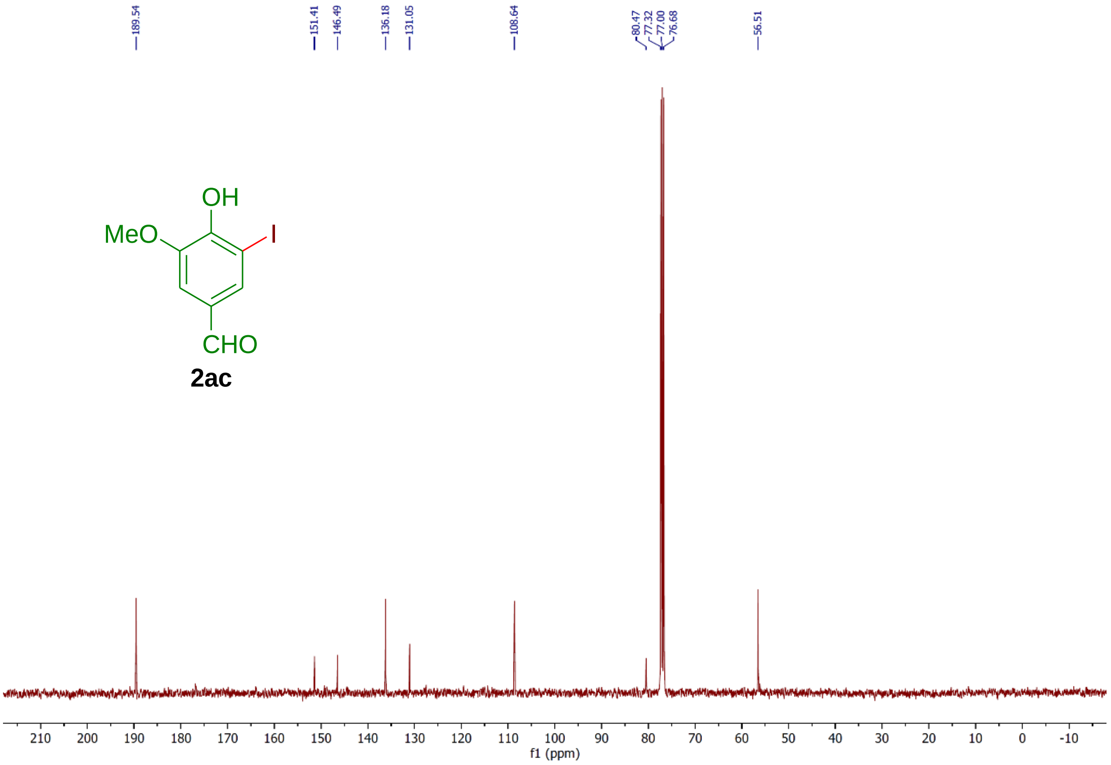

Figure S58: ^{13}C NMR spectrum of compound **2ac**, ($CDCl_3$, 100 MHz).

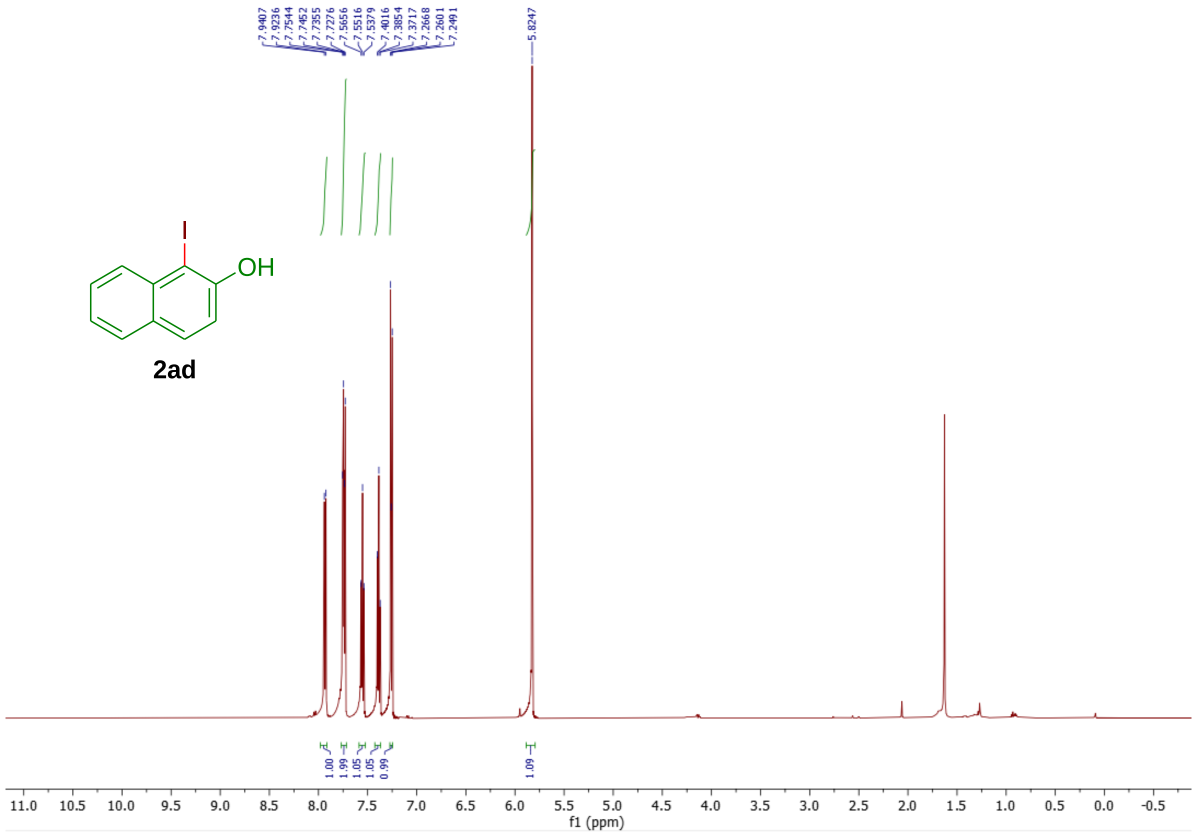

Figure S59: ^{1}H NMR spectrum of compound **2ad**, ($CDCl_3$, 500 MHz).

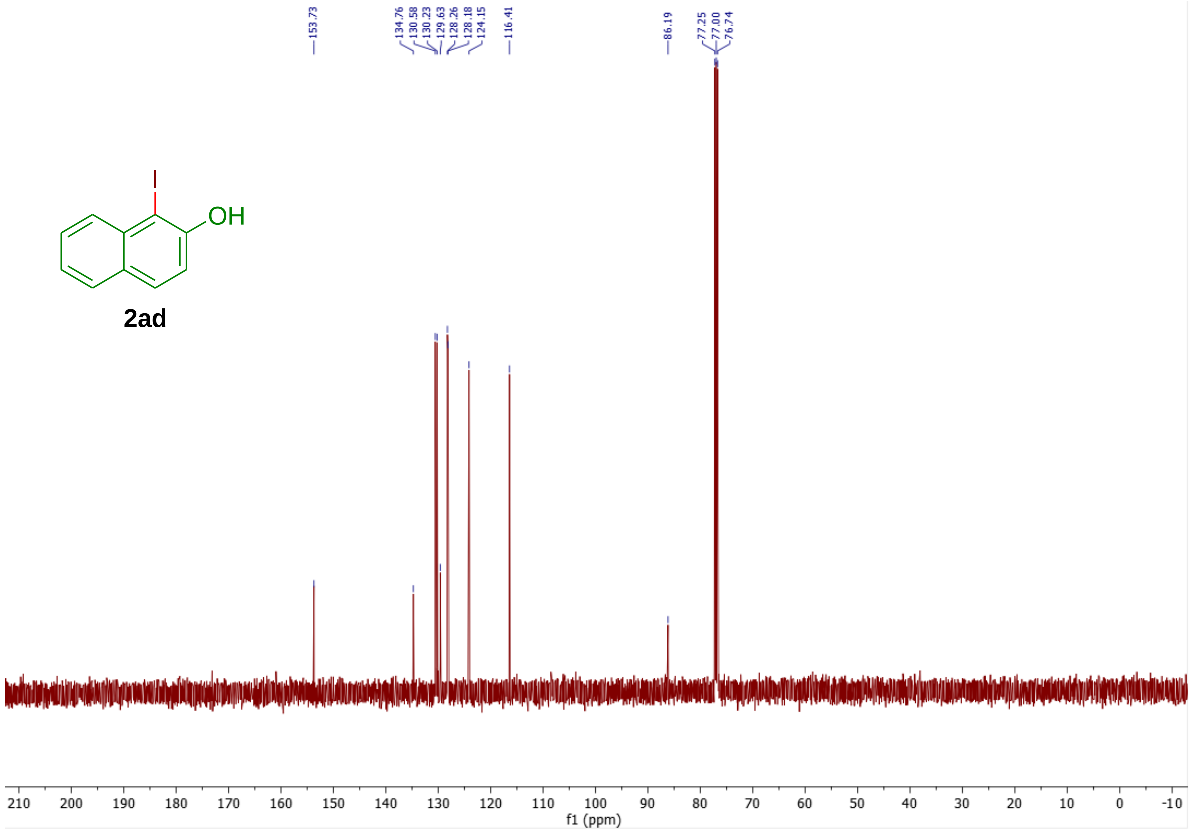

Figure S60: ^{13}C NMR spectrum of compound **2ad**, ($CDCl_3$, 125 MHz).

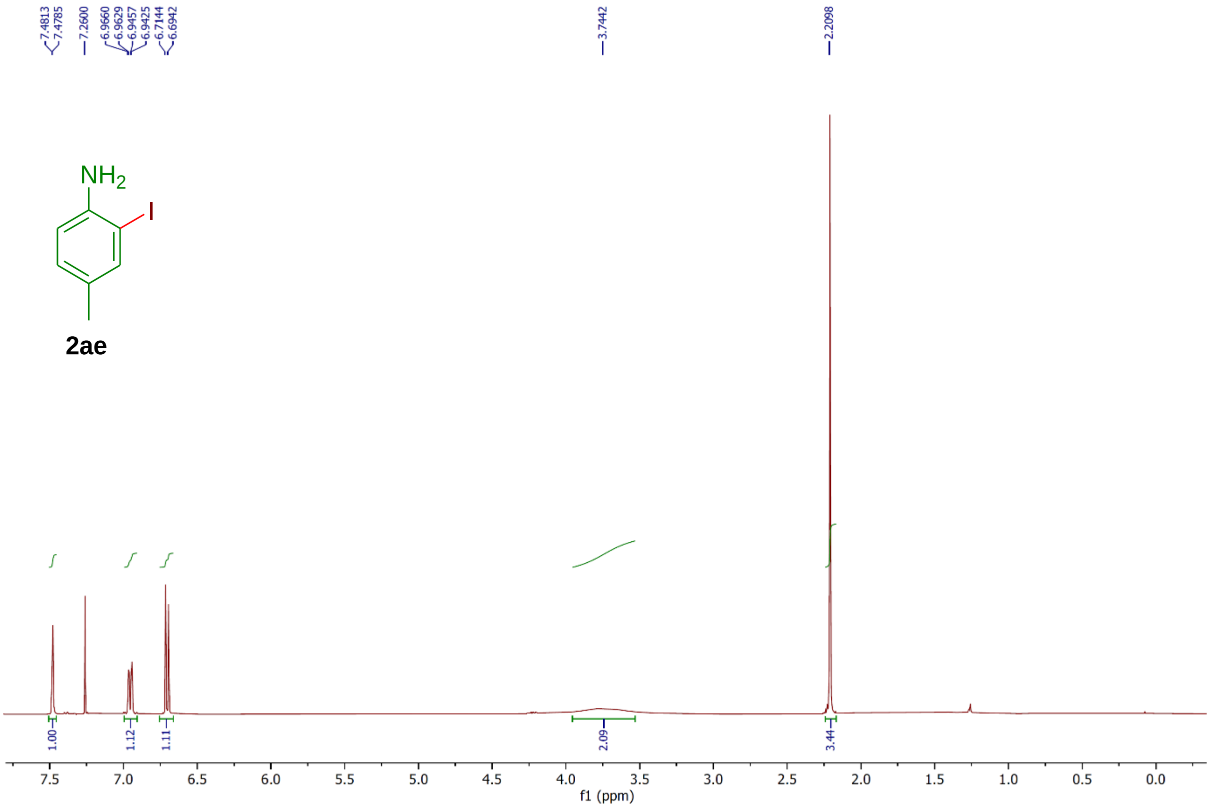

Figure S61: ^{1}H NMR spectrum of compound **2ae**, ($CDCl_3$, 400 MHz).

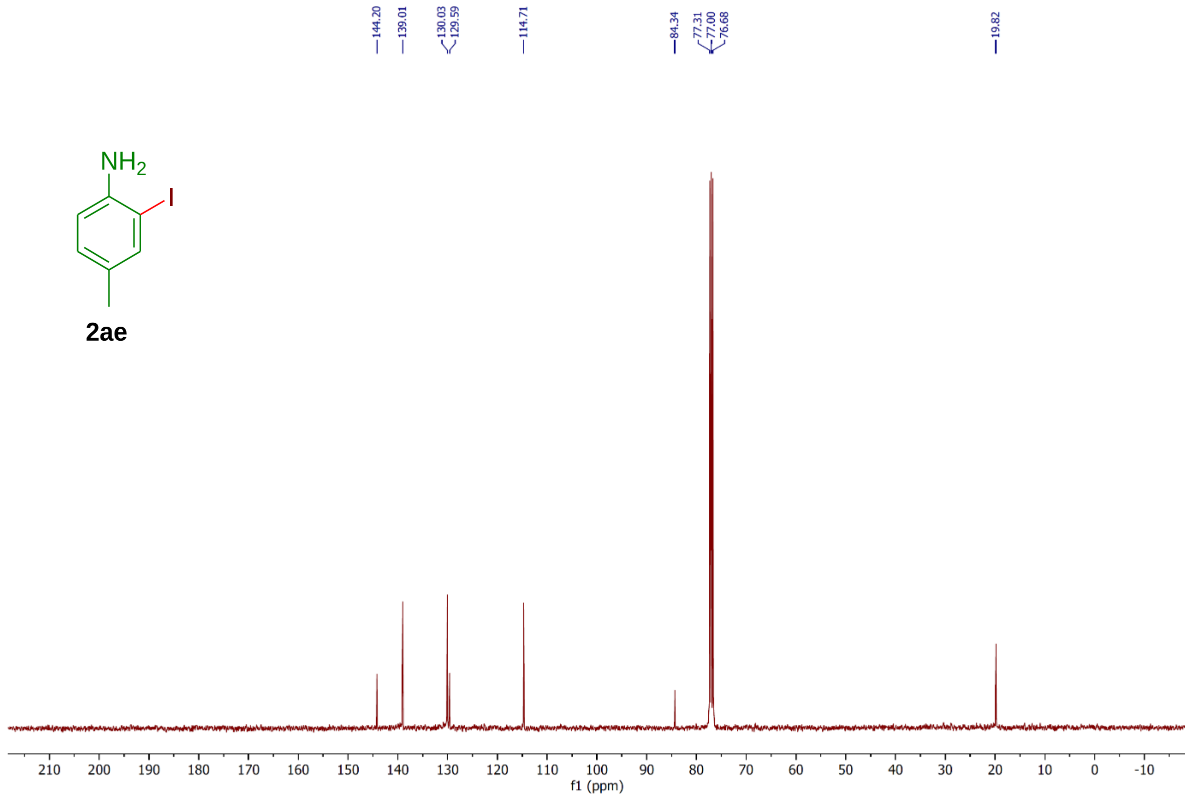

Figure S62: ^{13}C NMR spectrum of compound **2ae**, ($CDCl_3$, 100 MHz).

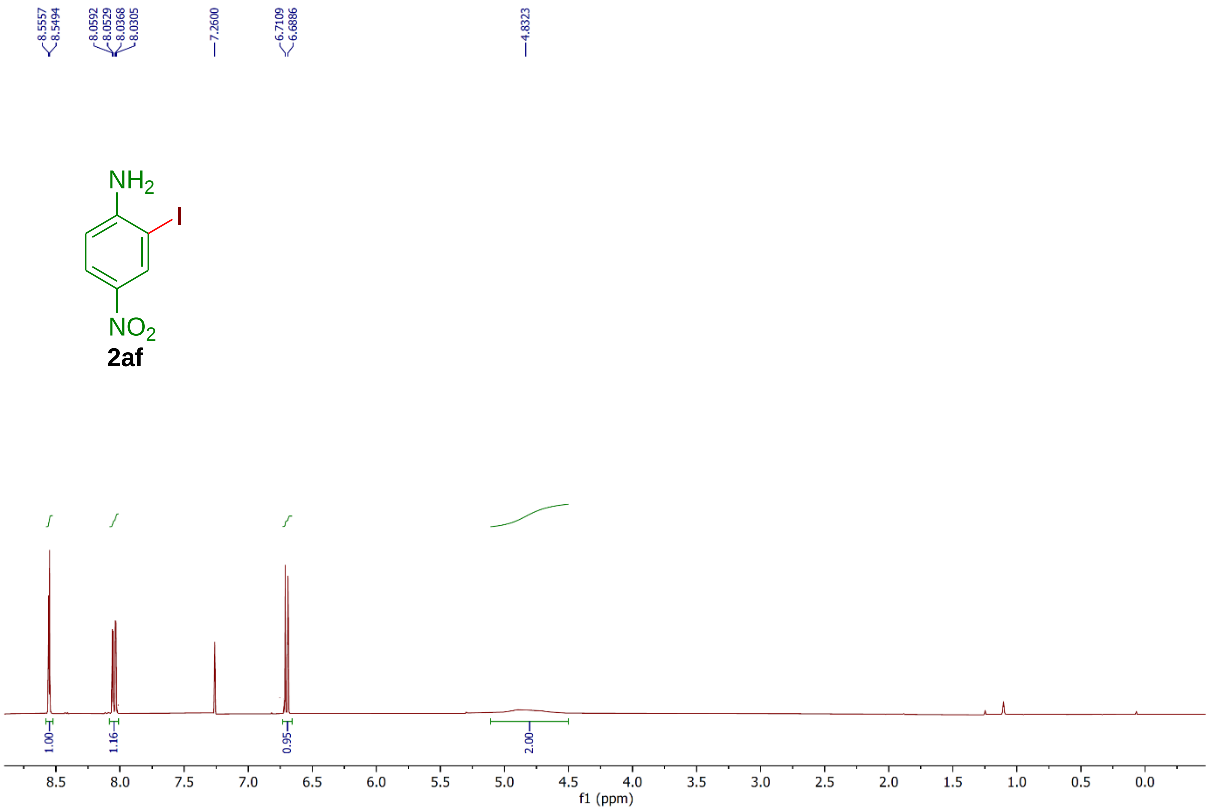

Figure S63: ^{1}H NMR spectrum of compound **2af**, ($CDCl_3$, 400 MHz).

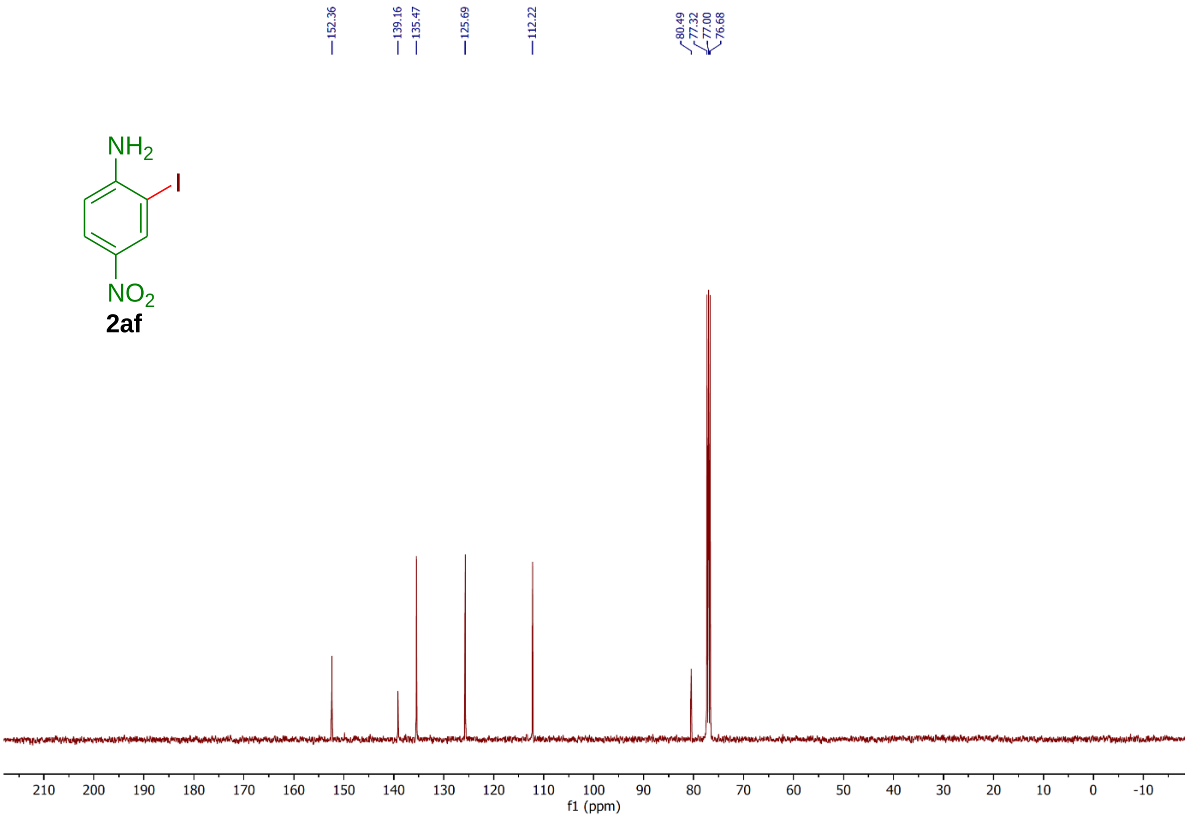

Figure S64: ^{13}C NMR spectrum of compound **2af**, ($CDCl_3$, 100 MHz).

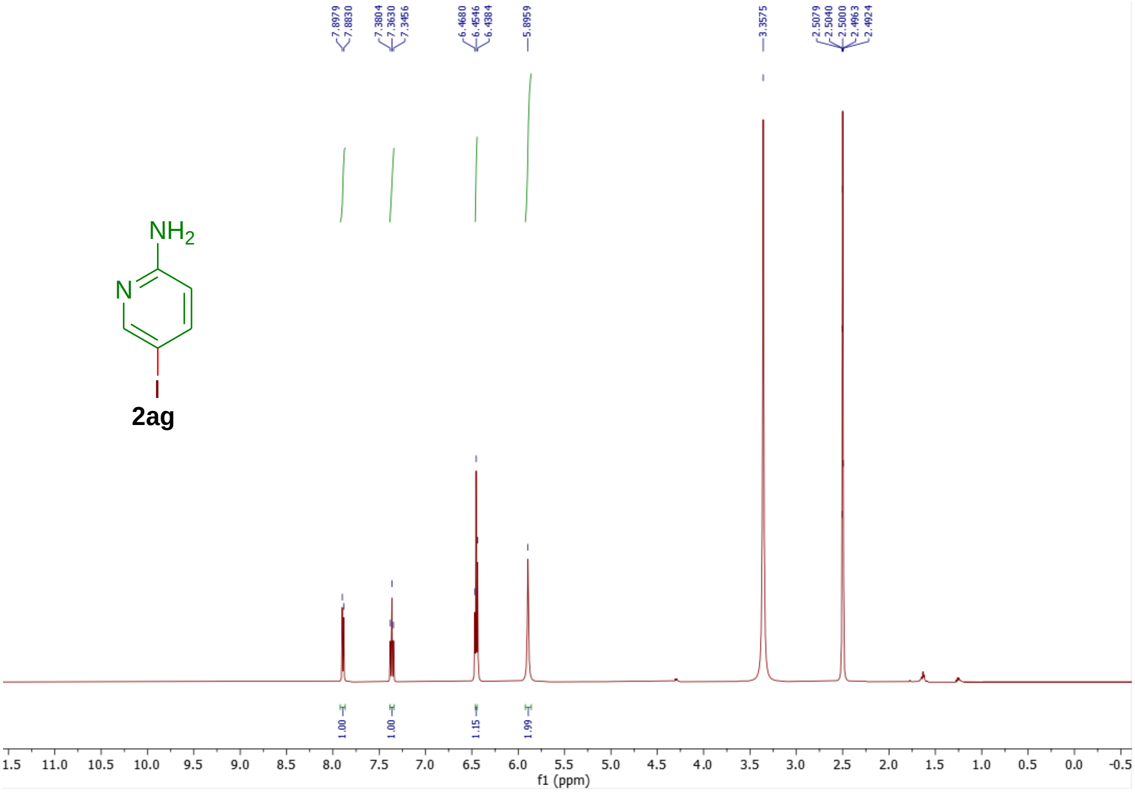

Figure S65: ^{1}H NMR spectrum of compound **2ag**, (DMSO-d_6, 500 MHz).

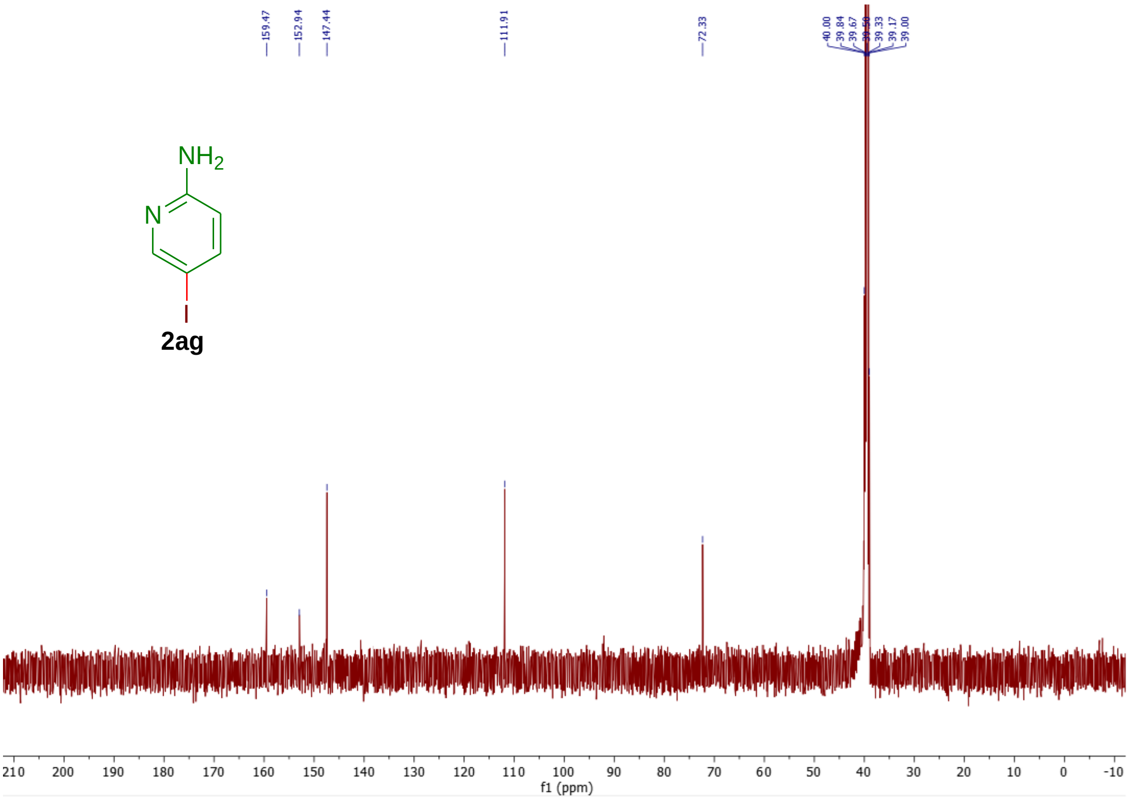

Figure S66: ^{13}C NMR spectrum of compound **2ag**, (DMSO-d_6, 125 MHz).

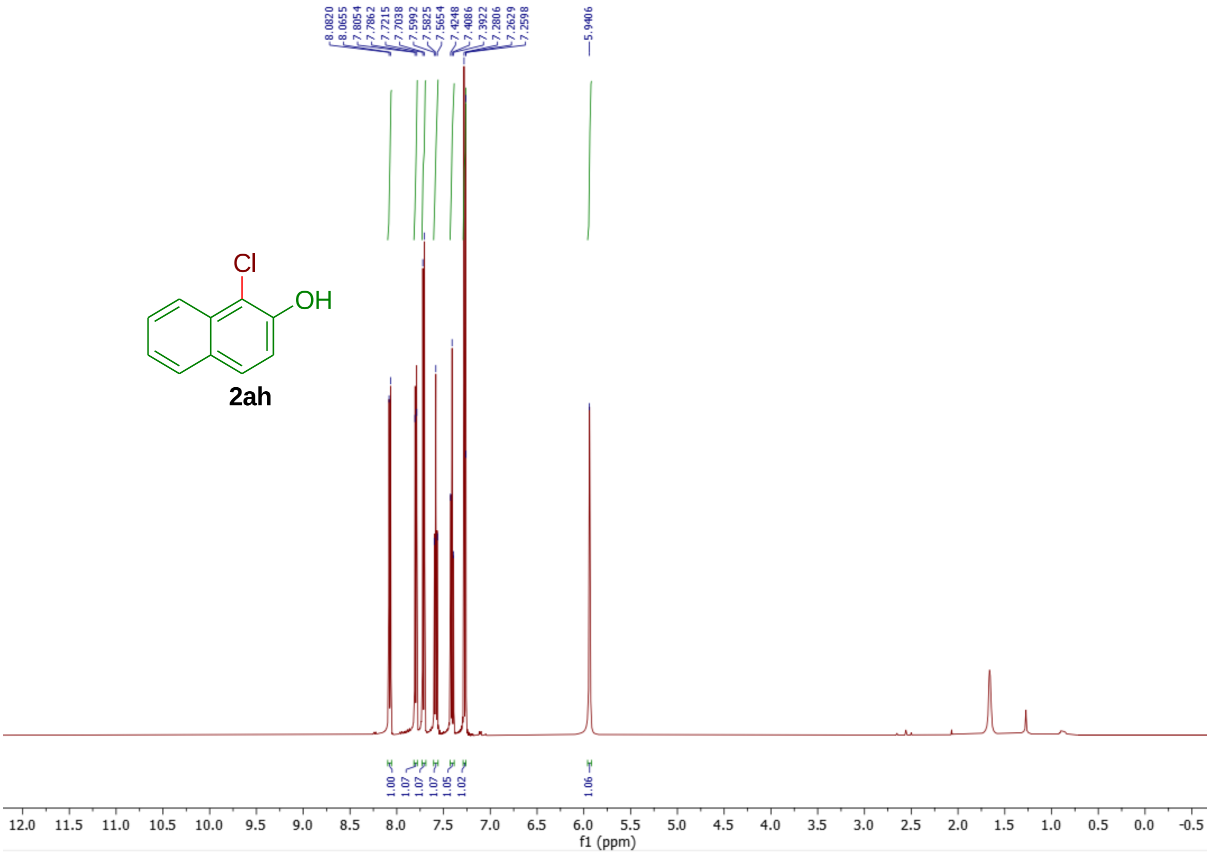

Figure S67: ^{1}H NMR spectrum of compound **2ah**, ($CDCl_3$, 500 MHz).

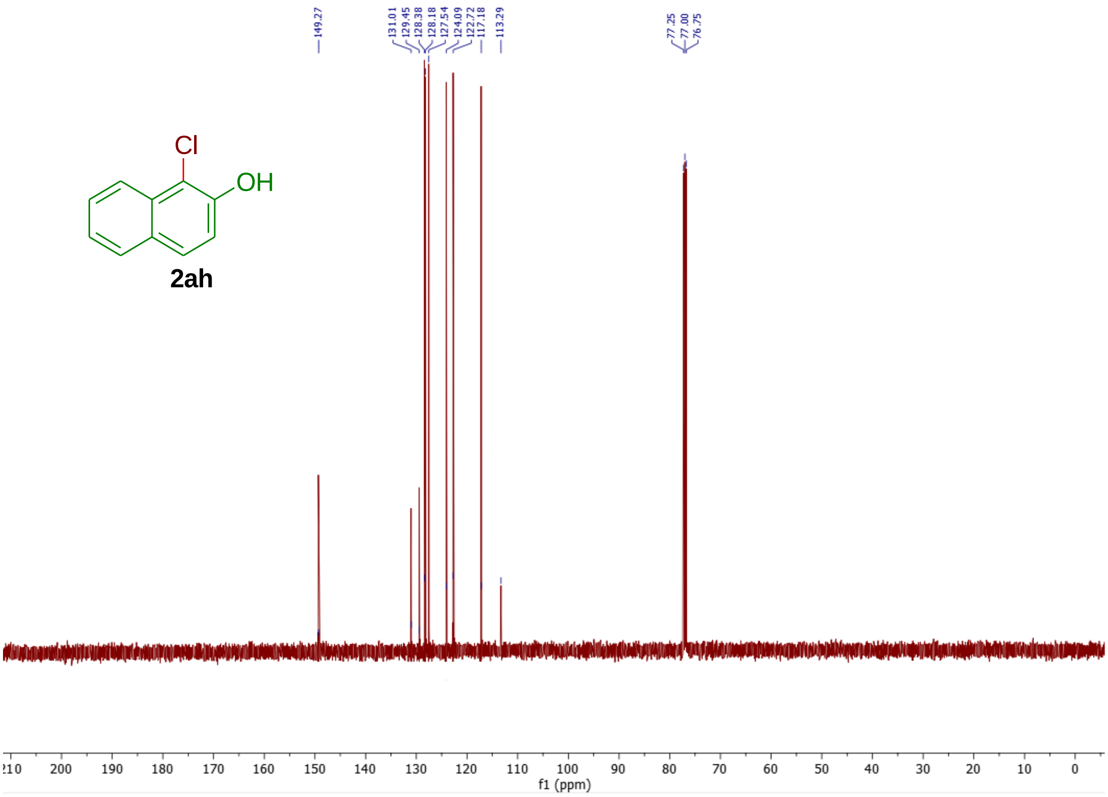

Figure S68: ^{13}C NMR spectrum of compound **2ah**, ($CDCl_3$, 125 MHz).

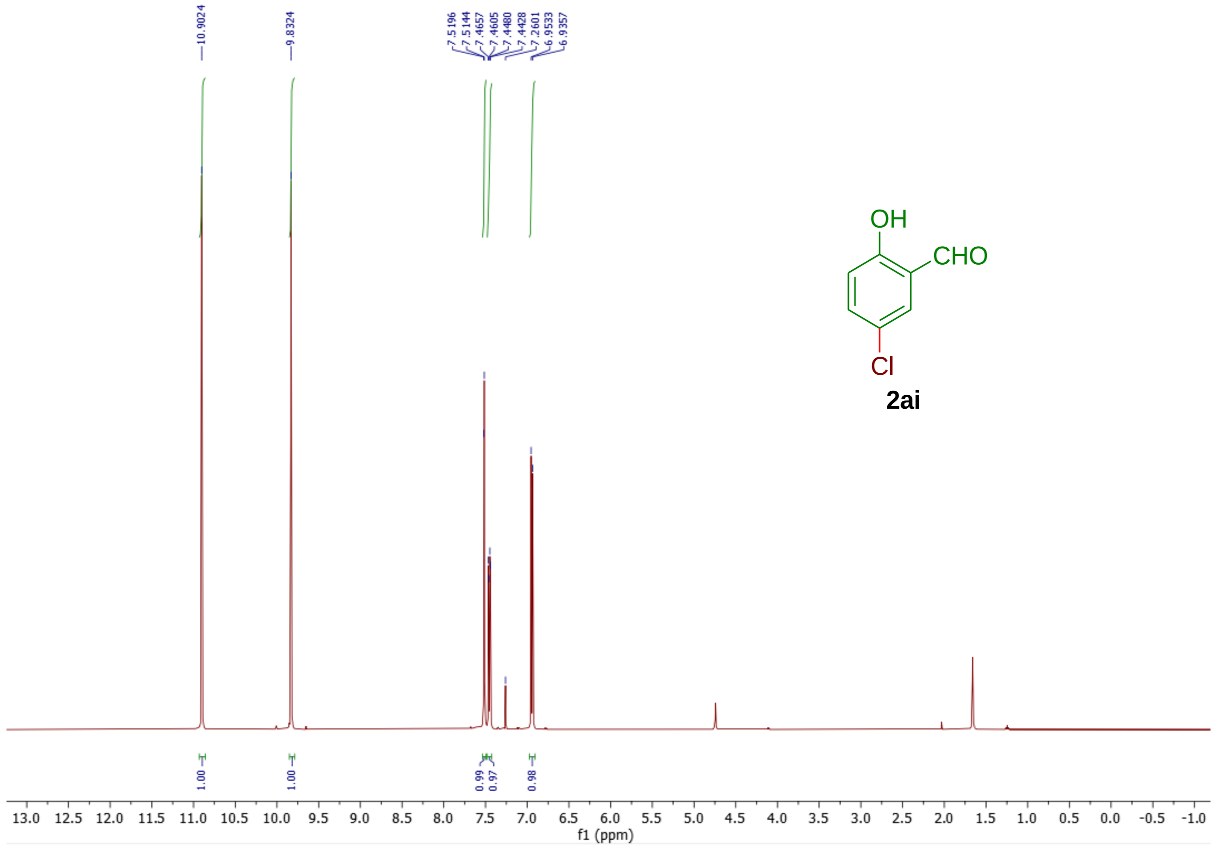

Figure S69: ^{1}H NMR spectrum of compound **2ai**, ($CDCl_3$, 500 MHz).

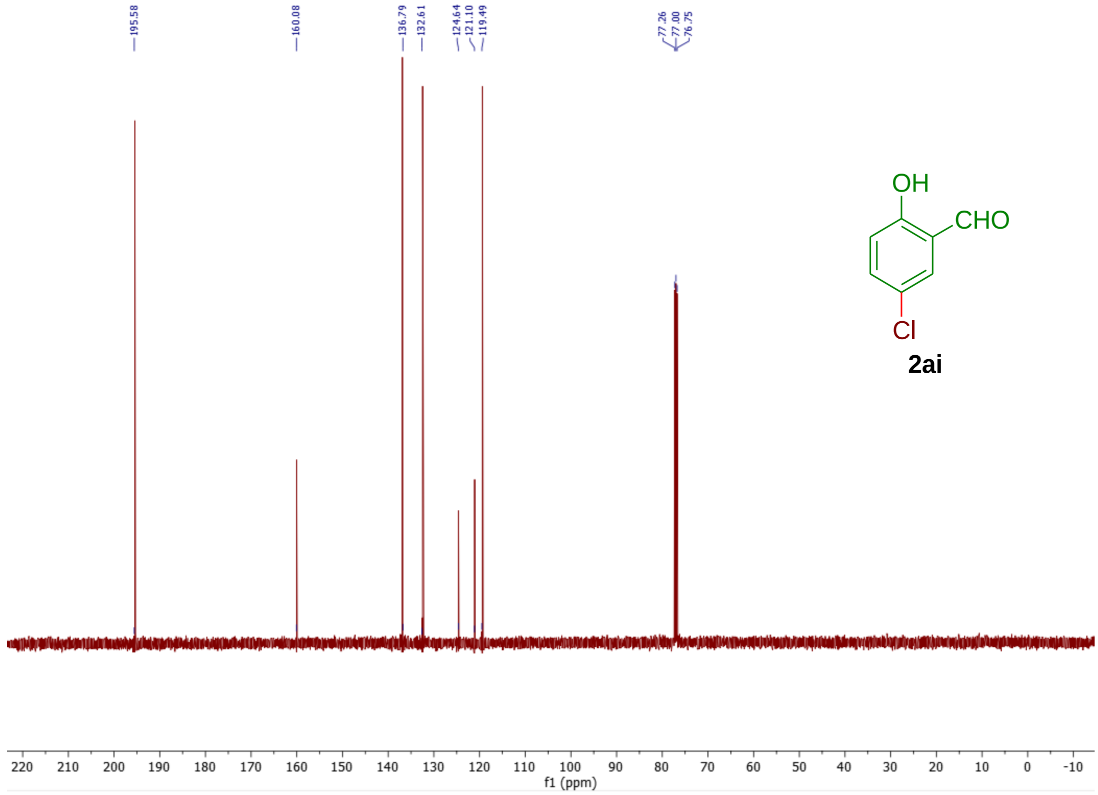

Figure S70: ^{13}C NMR spectrum of compound **2ai**, ($CDCl_3$, 125 MHz).

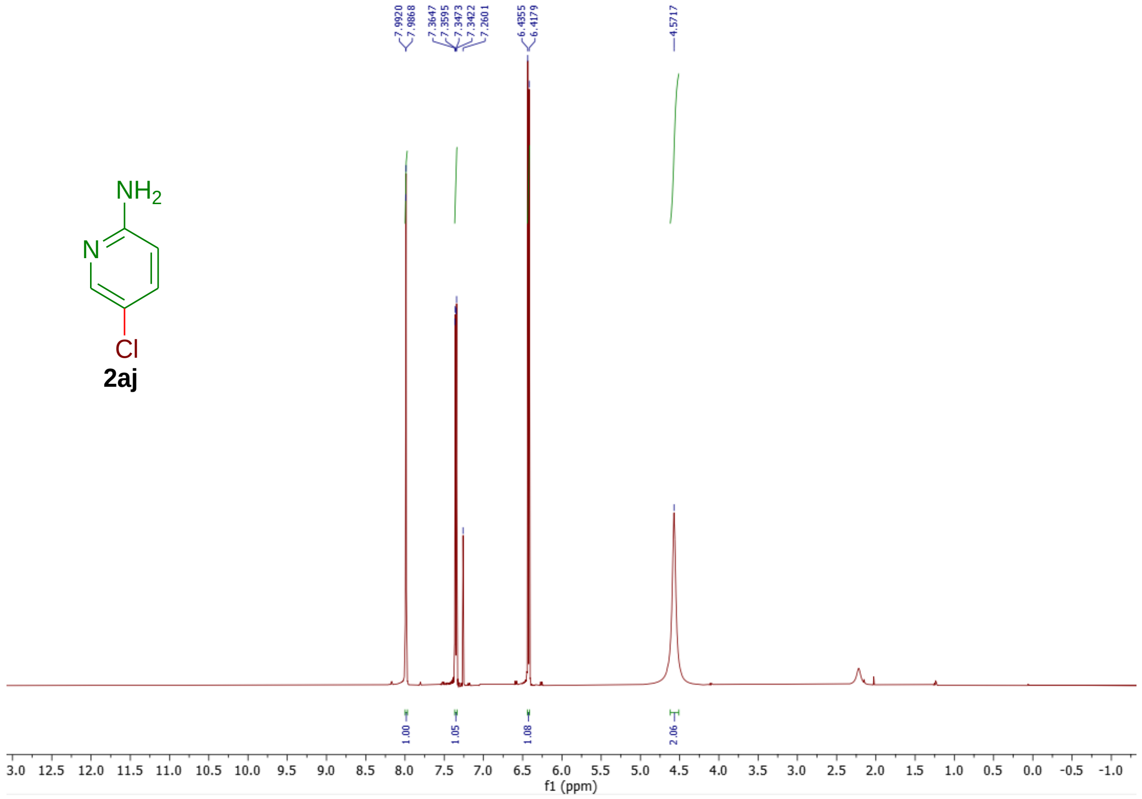

Figure S71: ^{1}H NMR spectrum of compound **2aj**, ($CDCl_3$, 500 MHz).

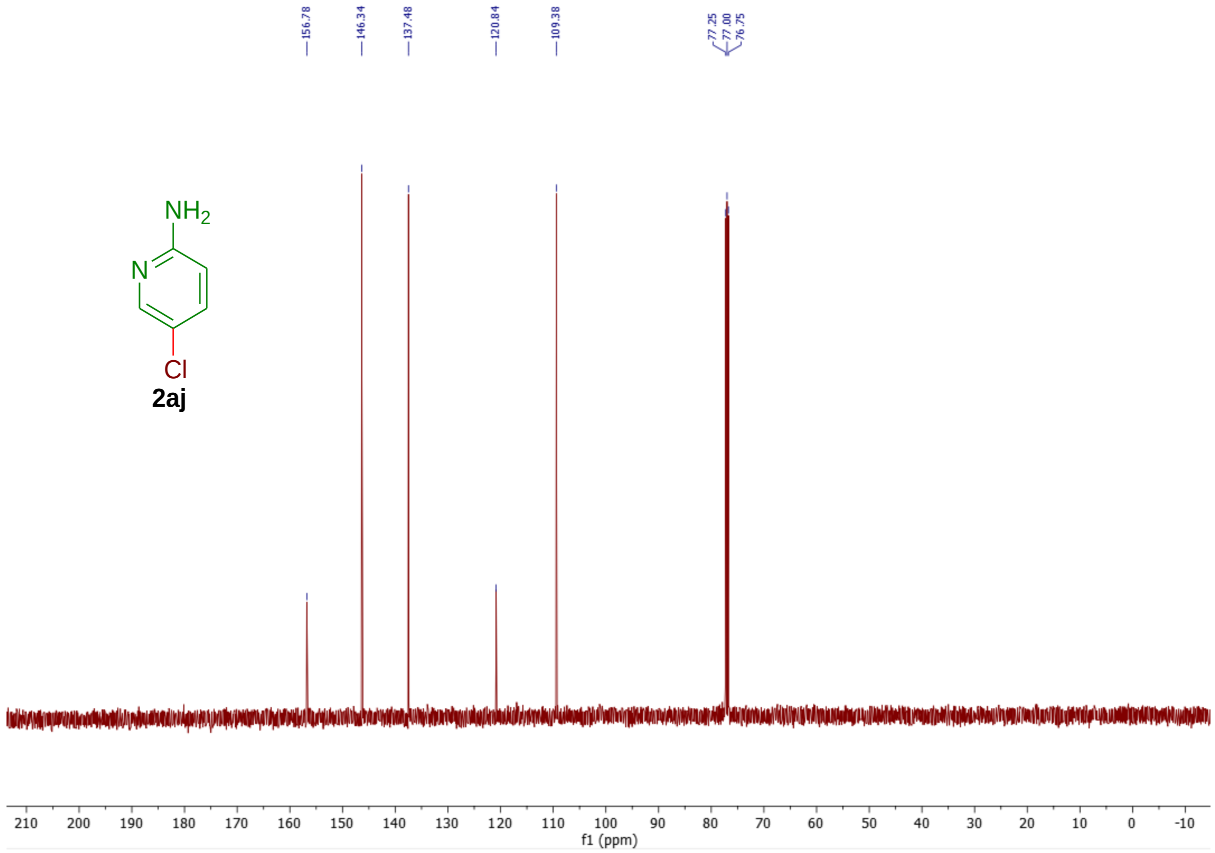

Figure S72: ^{13}C NMR spectrum of compound **2aj**, ($CDCl_3$, 125 MHz).

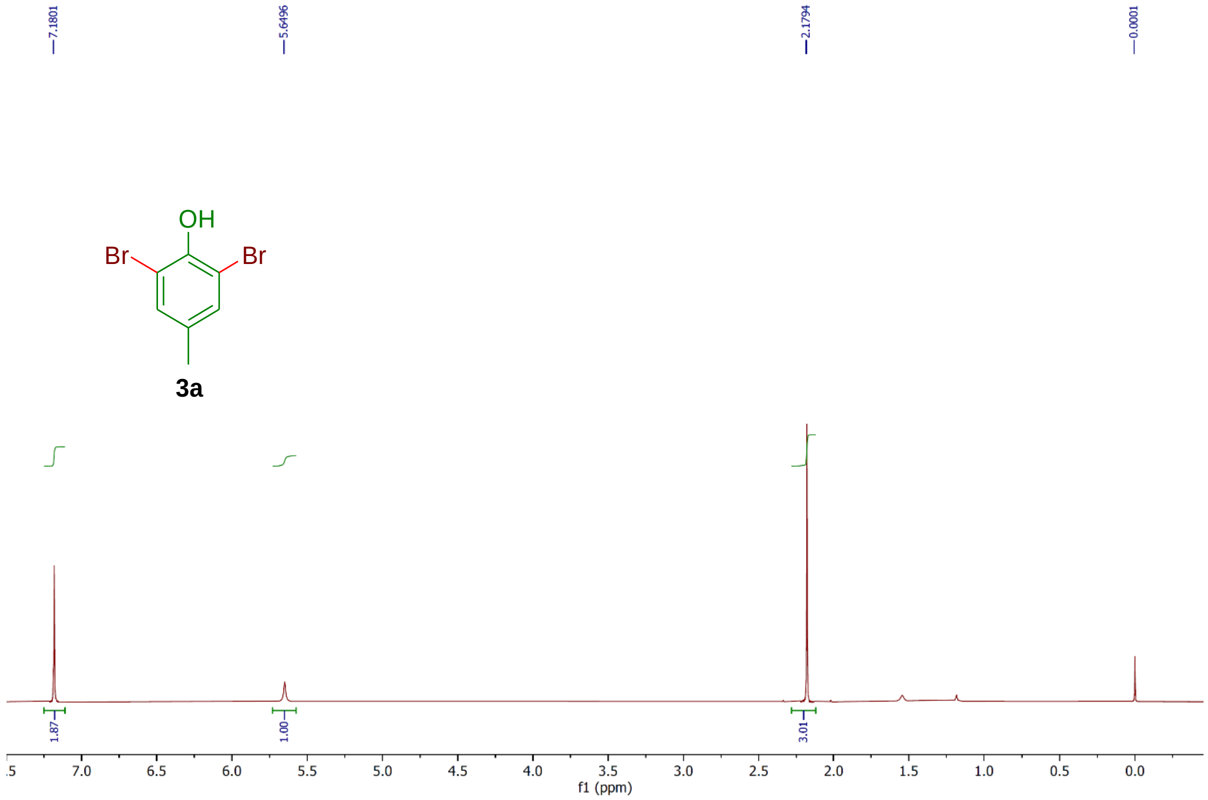

Figure S73: ^{1}H NMR spectrum of compound **3a**, ($CDCl_3$, 400 MHz).

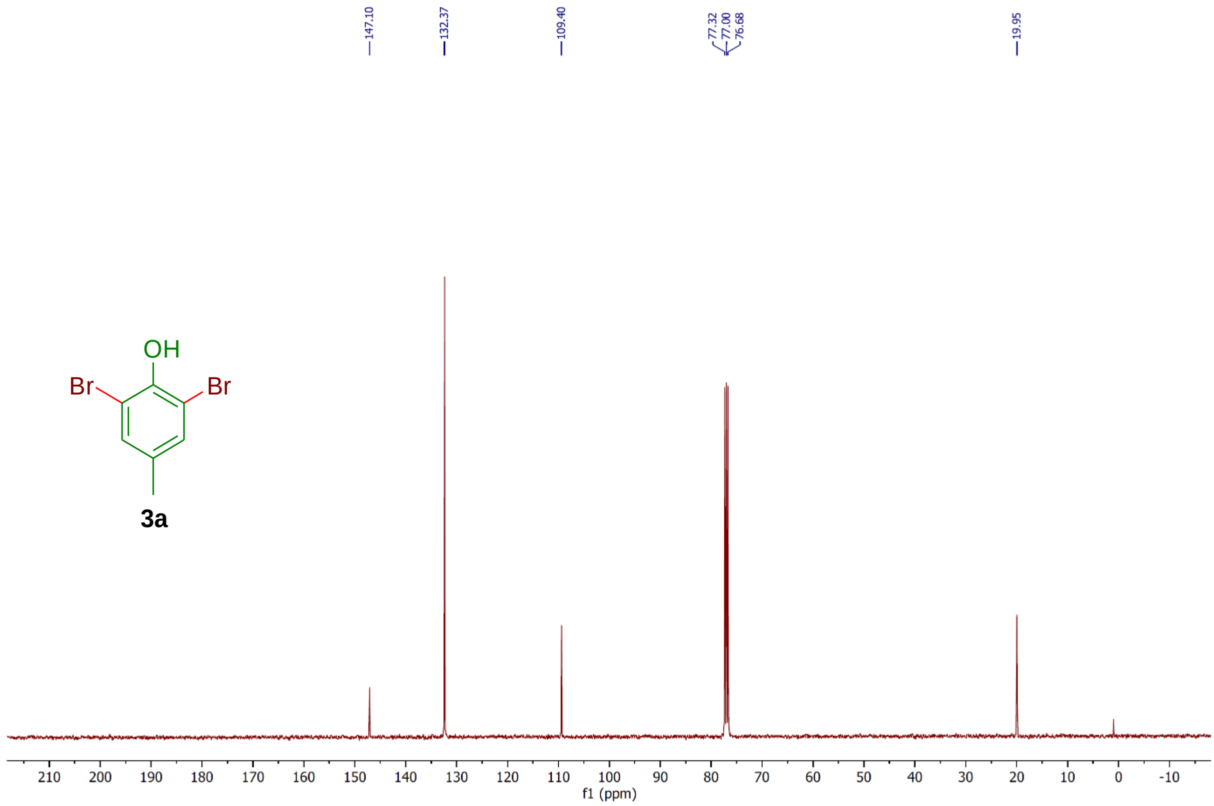

Figure S74: ^{13}C NMR spectrum of compound **3a**, ($CDCl_3$, 100 MHz).

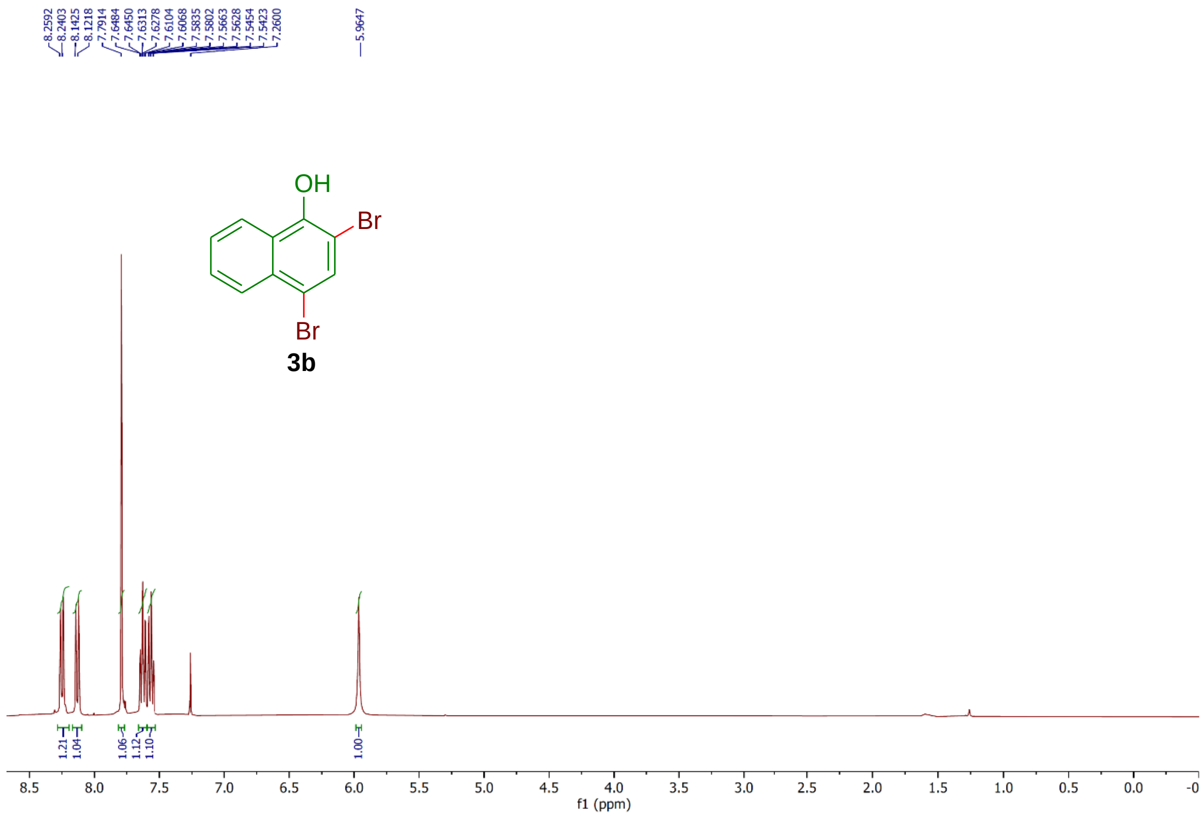

Figure S75: ^{1}H NMR spectrum of compound **3b**, ($CDCl_3$, 400 MHz).

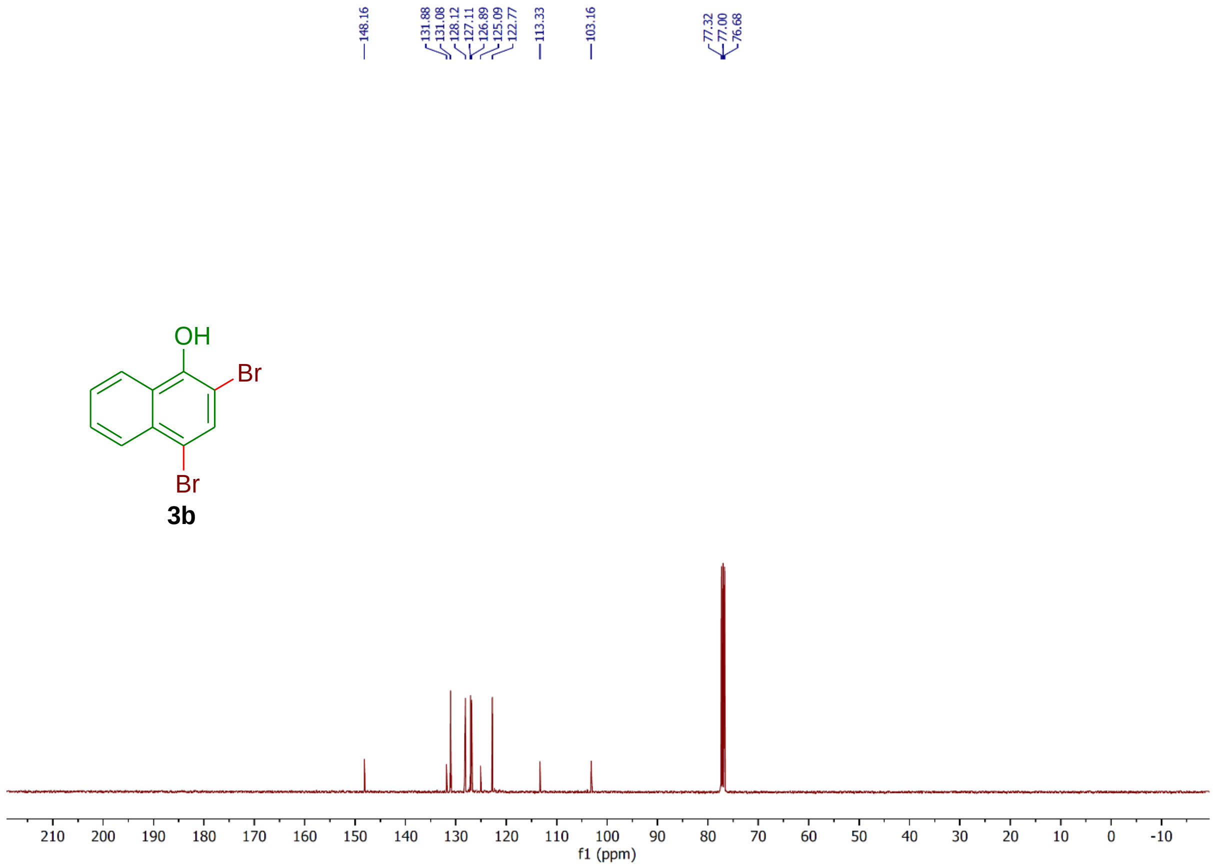

Figure S76: ^{13}C NMR spectrum of compound **3b**, ($CDCl_3$, 100 MHz).

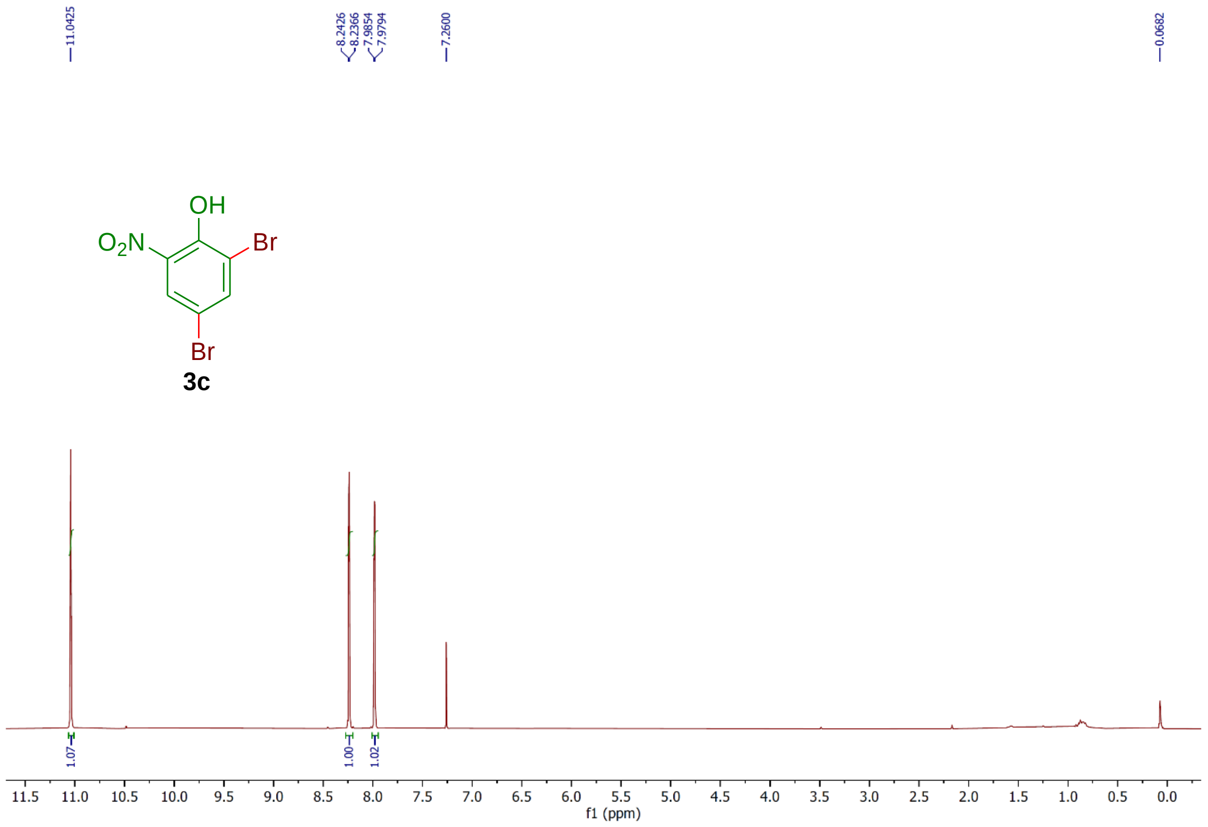

Figure S77: ^{1}H NMR spectrum of compound **3c**, ($CDCl_3$, 400 MHz).

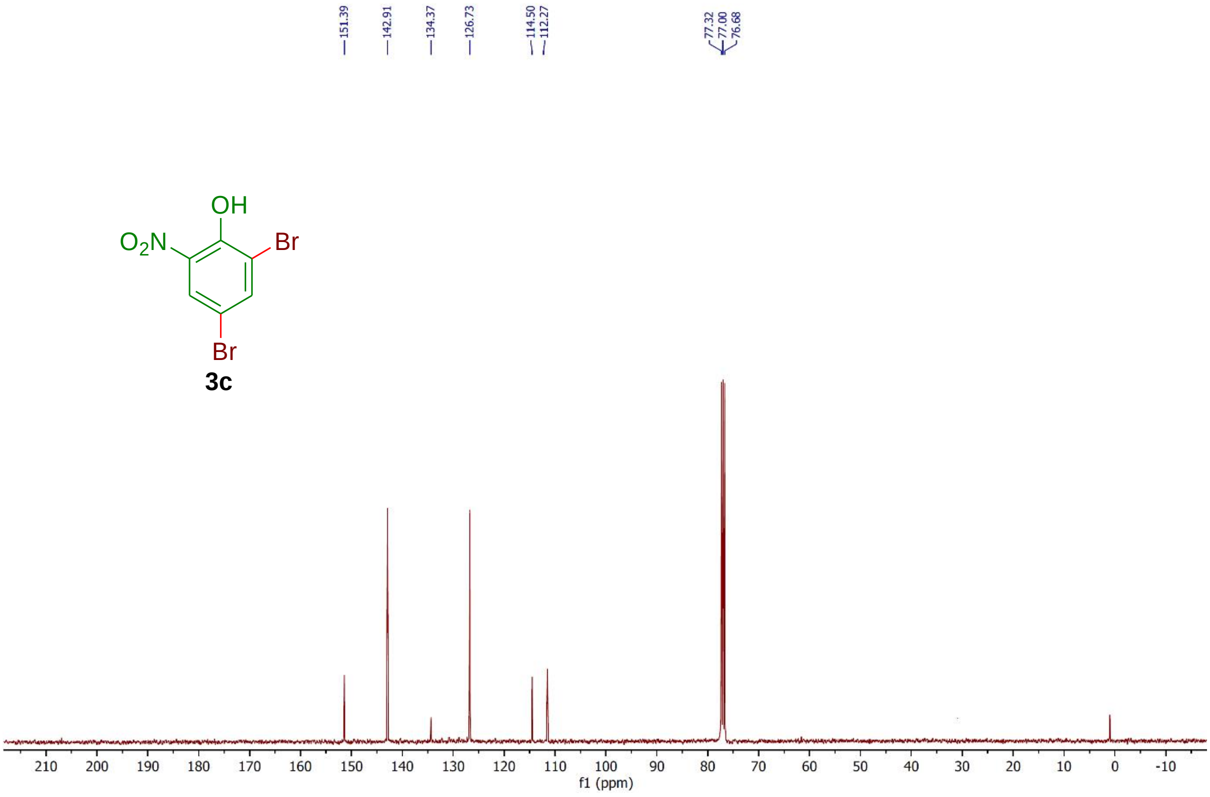

Figure S78: ^{13}C NMR spectrum of compound **3c**, ($CDCl_3$, 100 MHz).

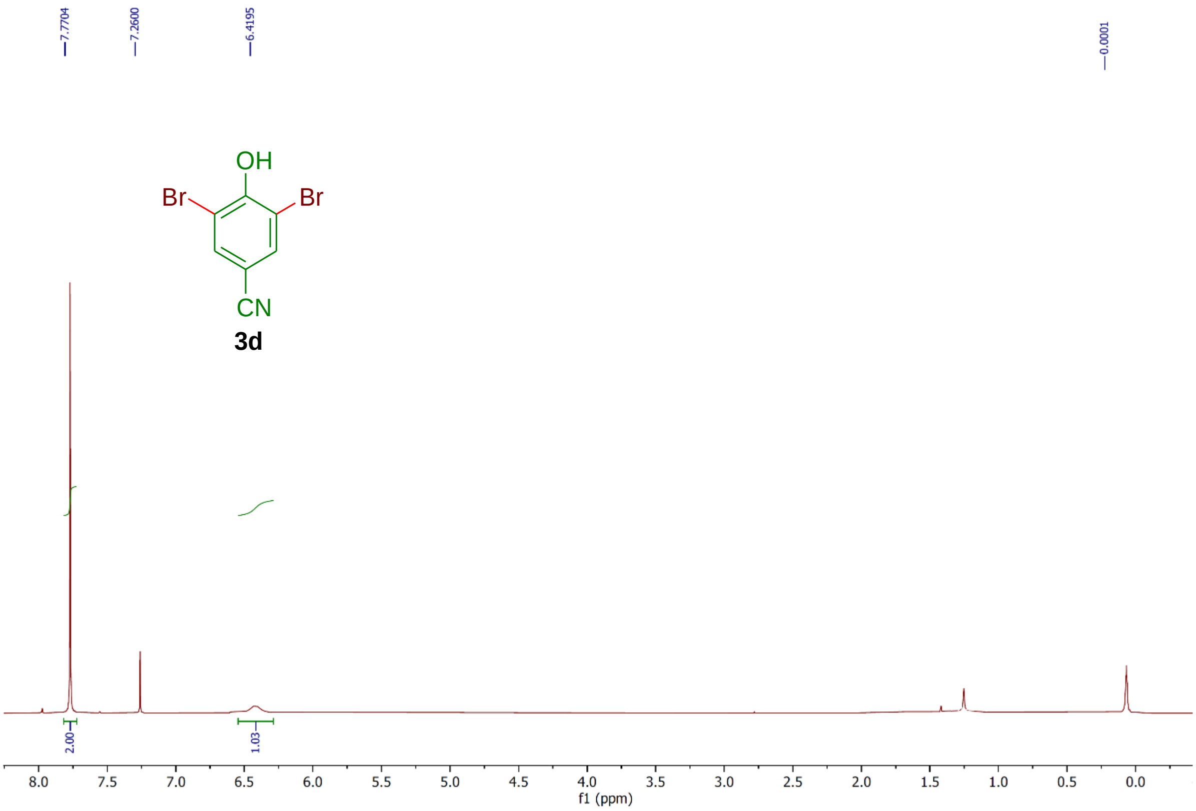

Figure S79: ¹H NMR spectrum of compound **3d**, ($CDCl_3$, 400 MHz).

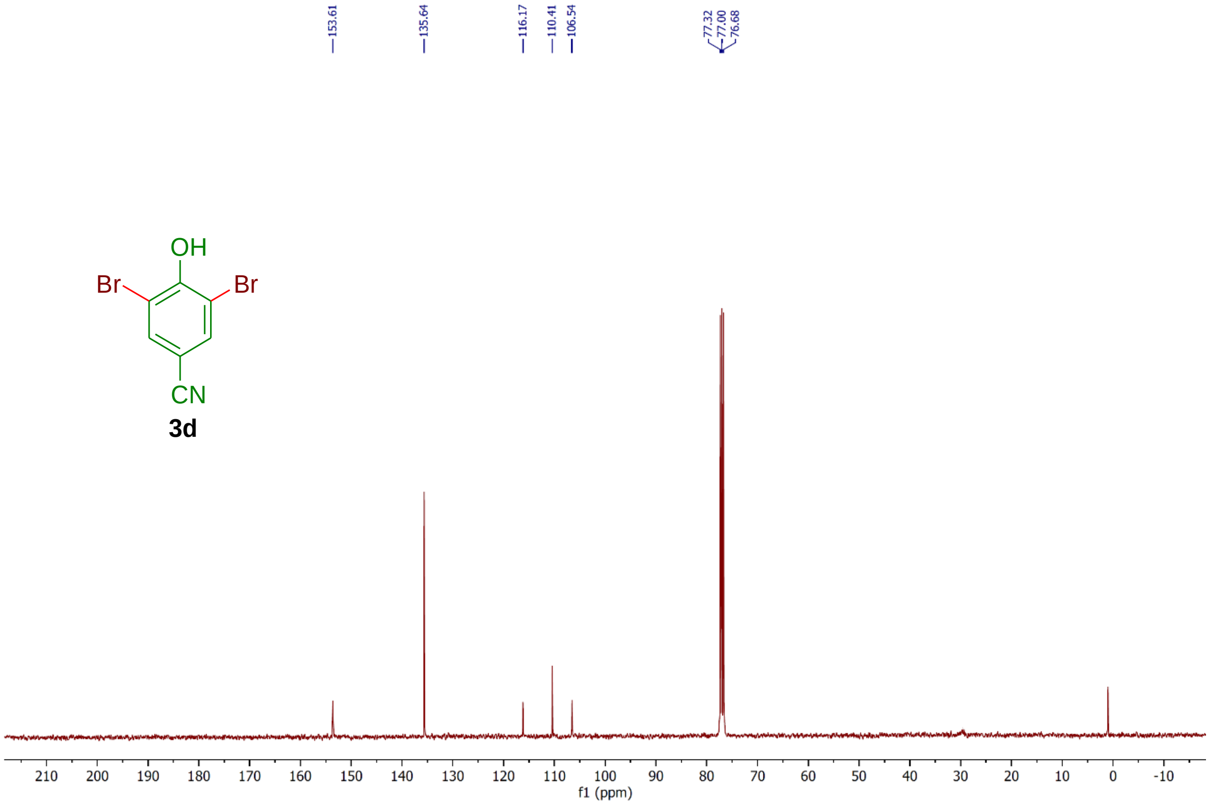

Figure S80: ¹³C NMR spectrum of compound **3d**, ($CDCl_3$, 100 MHz).

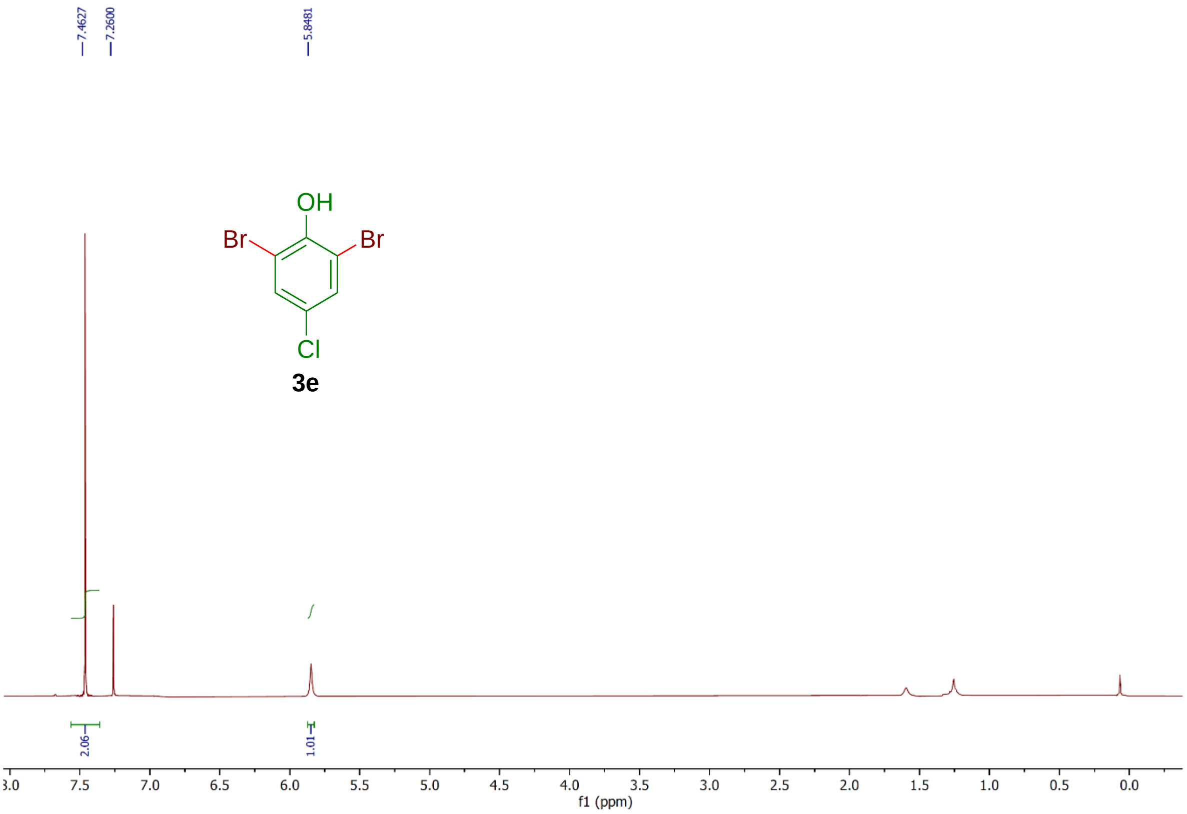

Figure S81: ^{1}H NMR spectrum of compound **3e**, ($CDCl_3$, 400 MHz).

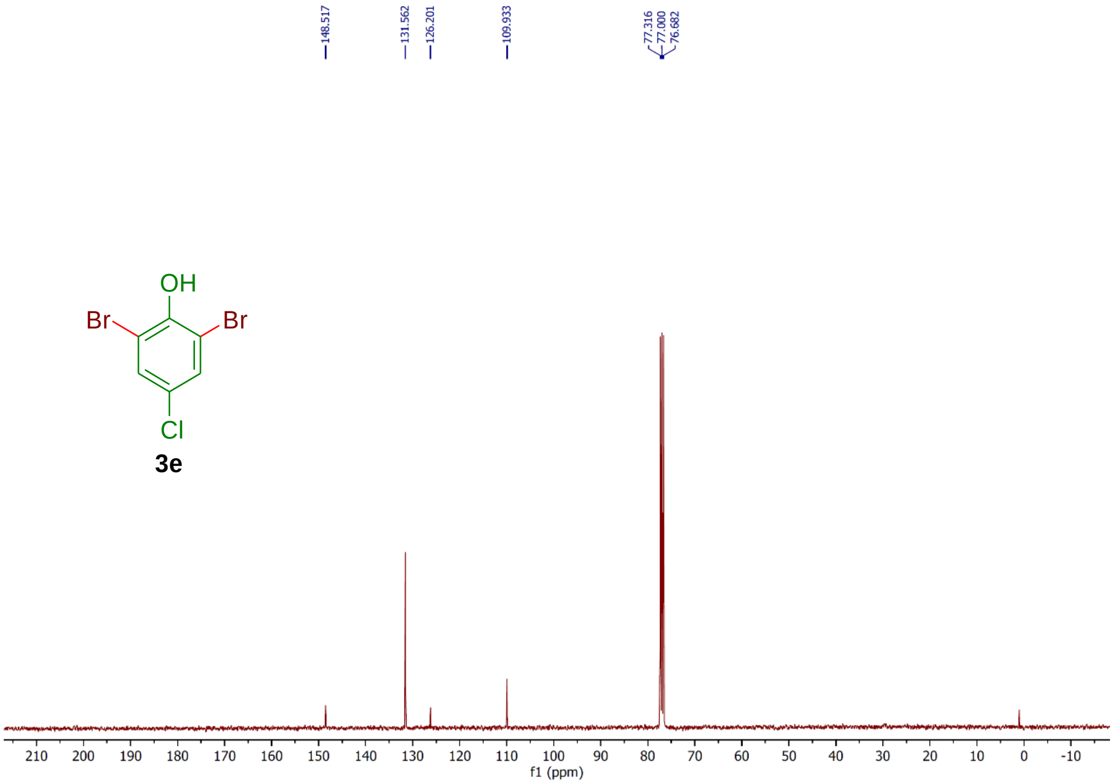

Figure S82: ^{13}C NMR spectrum of compound **3e**, ($CDCl_3$, 100 MHz).

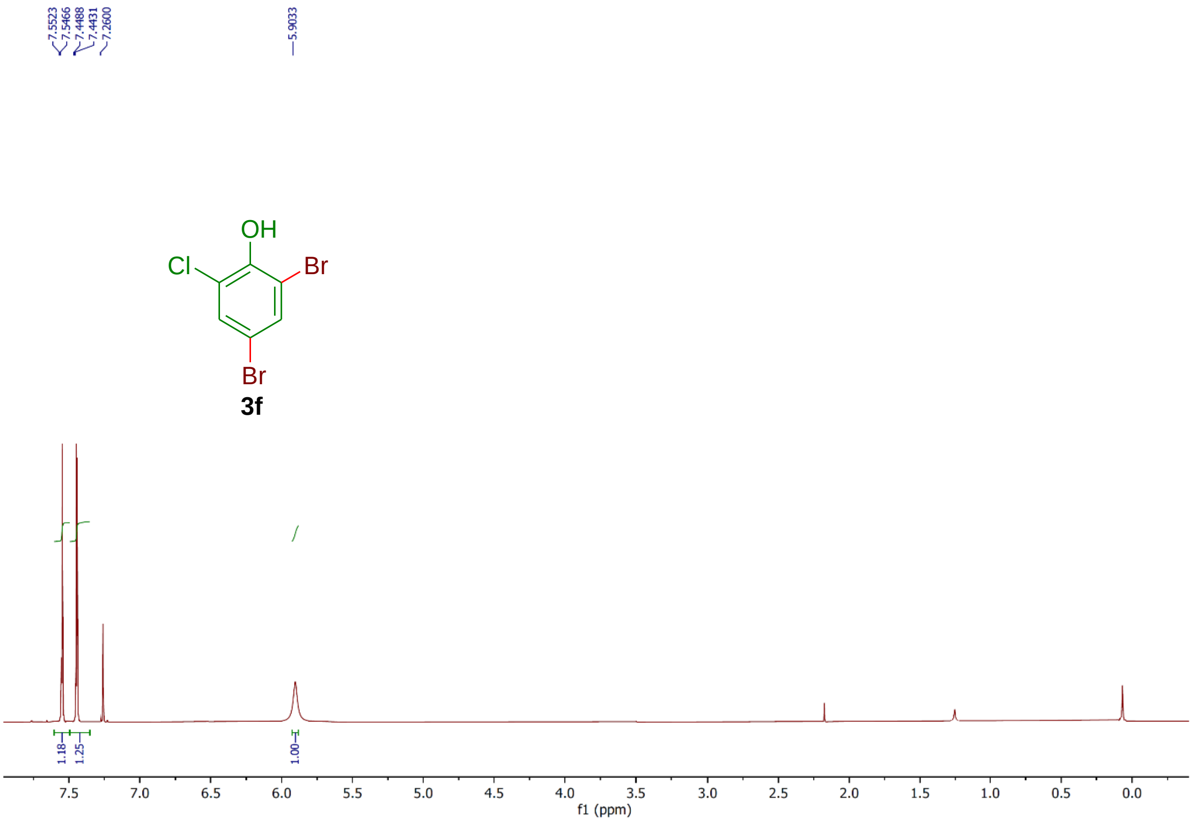

Figure S83: ^{1}H NMR spectrum of compound **3f**, ($CDCl_3$, 400 MHz).

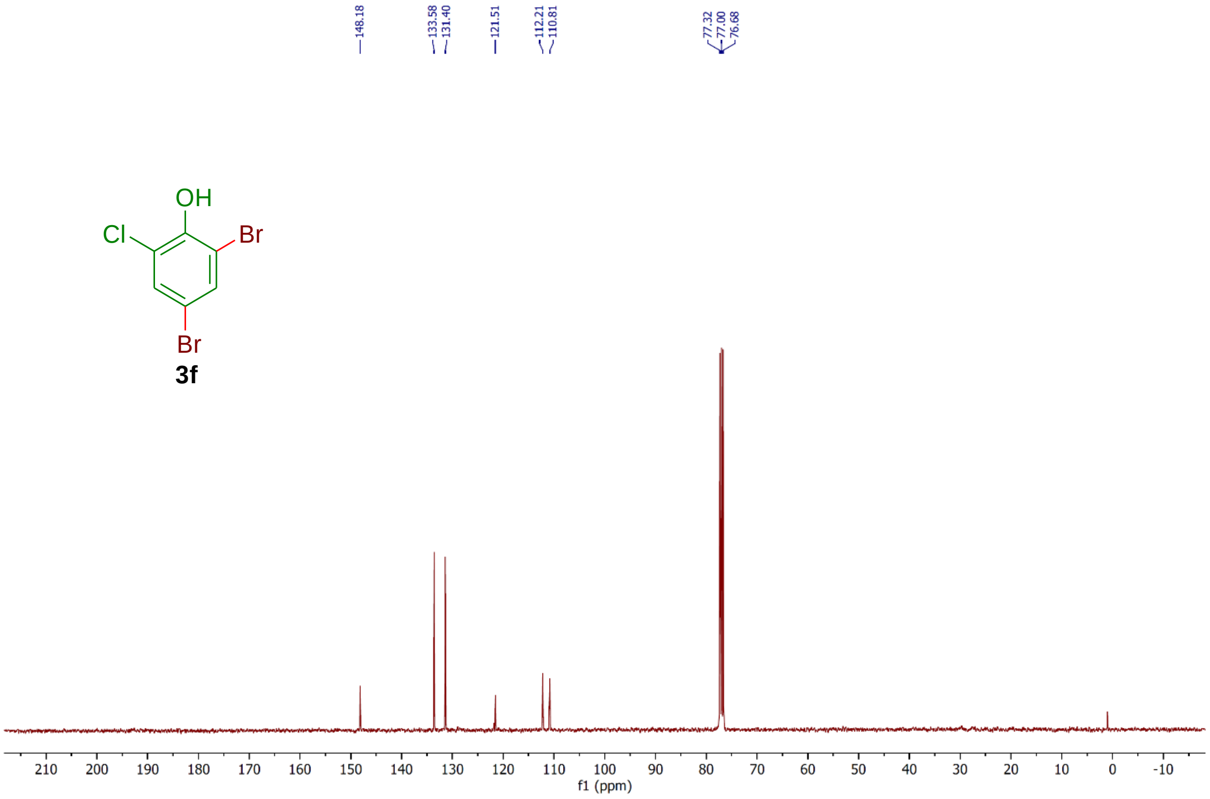

Figure S84: ^{13}C NMR spectrum of compound **3f**, ($CDCl_3$, 100 MHz).

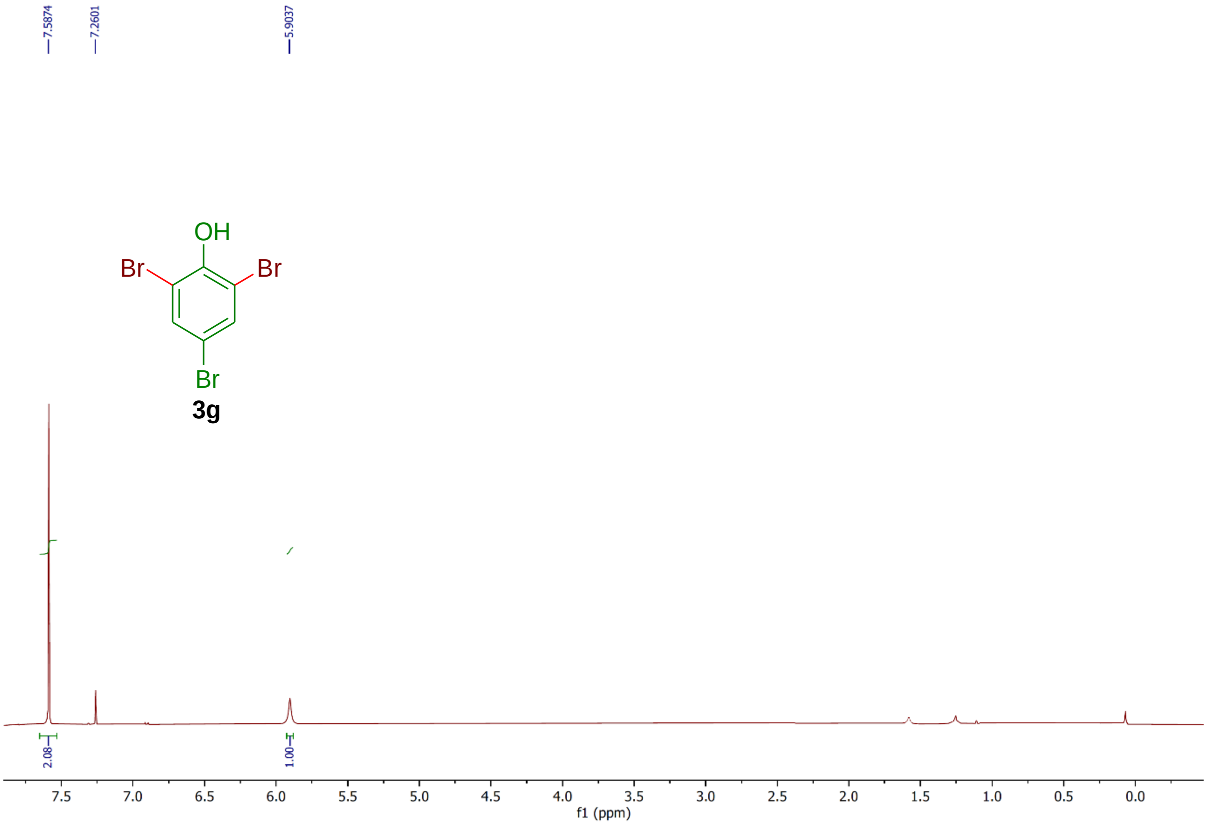

Figure S85: ^{1}H NMR spectrum of compound **3g**, ($CDCl_3$, 400 MHz).

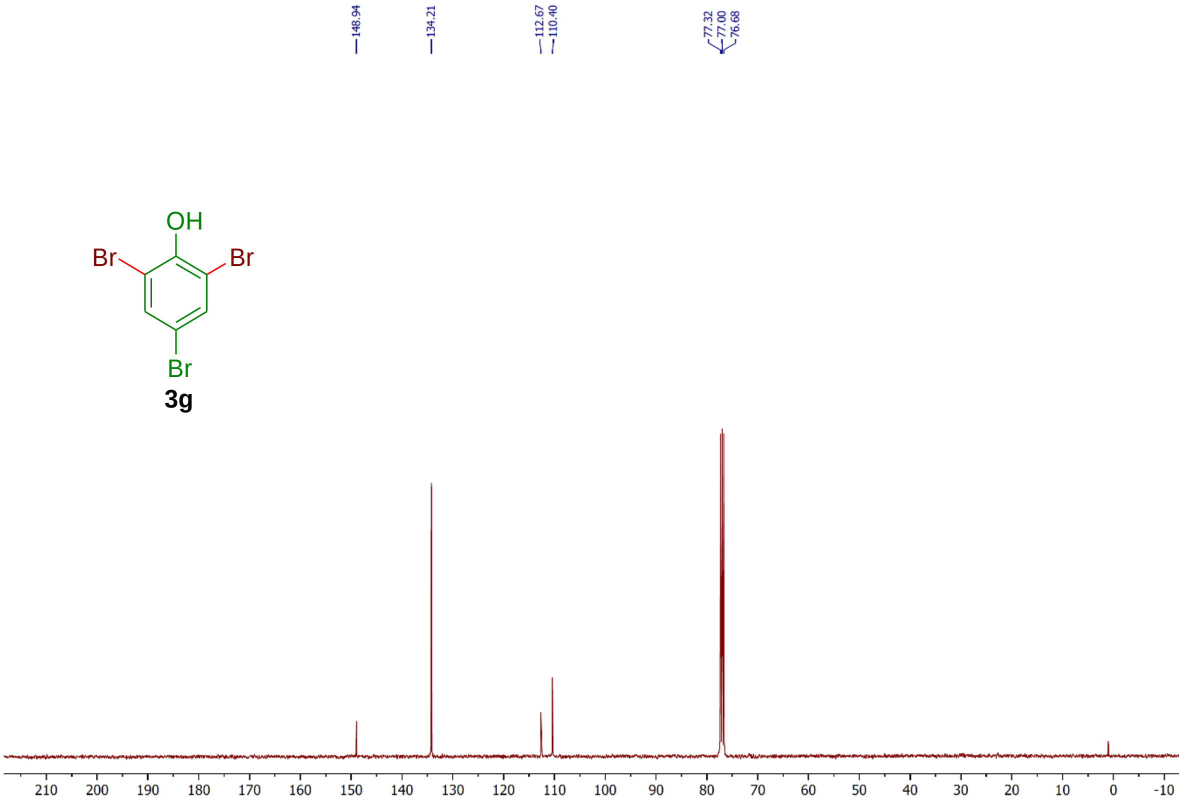

Figure S86: ^{13}C NMR spectrum of compound **3g**, ($CDCl_3$, 100 MHz).

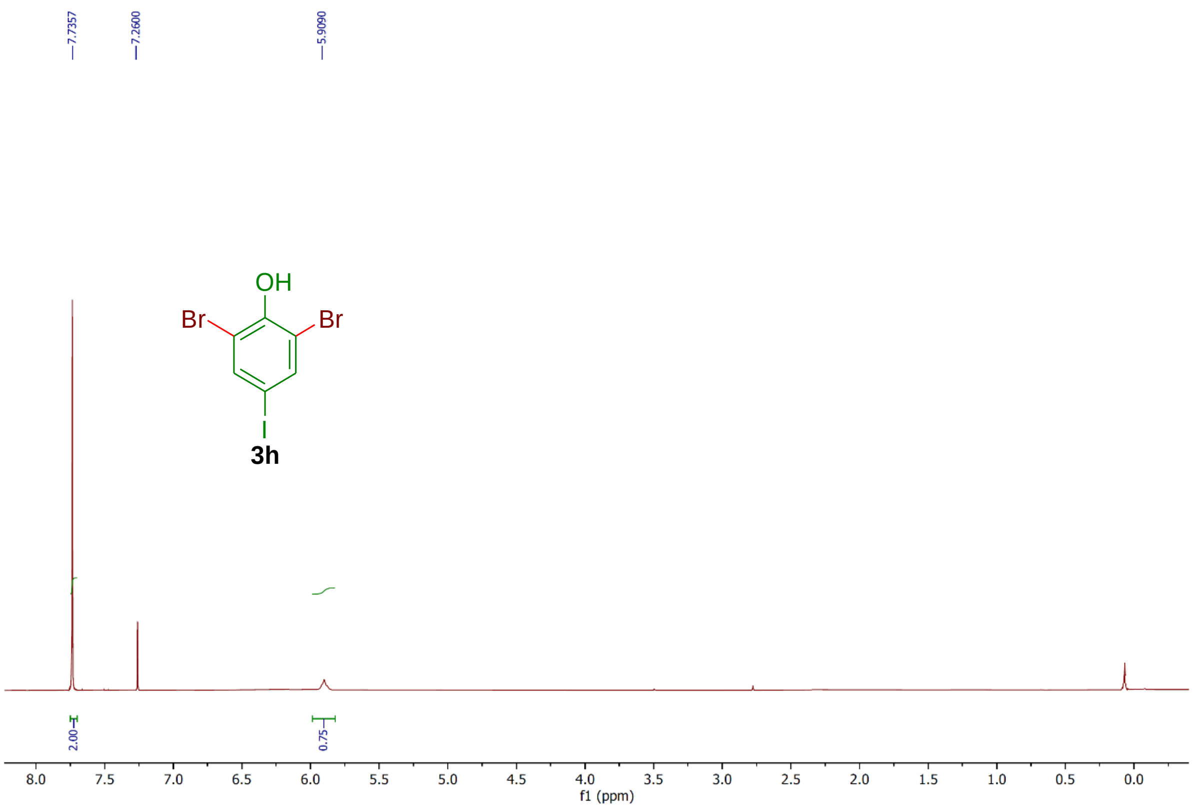

Figure S87: ^{1}H NMR spectrum of compound **3h**, ($CDCl_3$, 400 MHz).

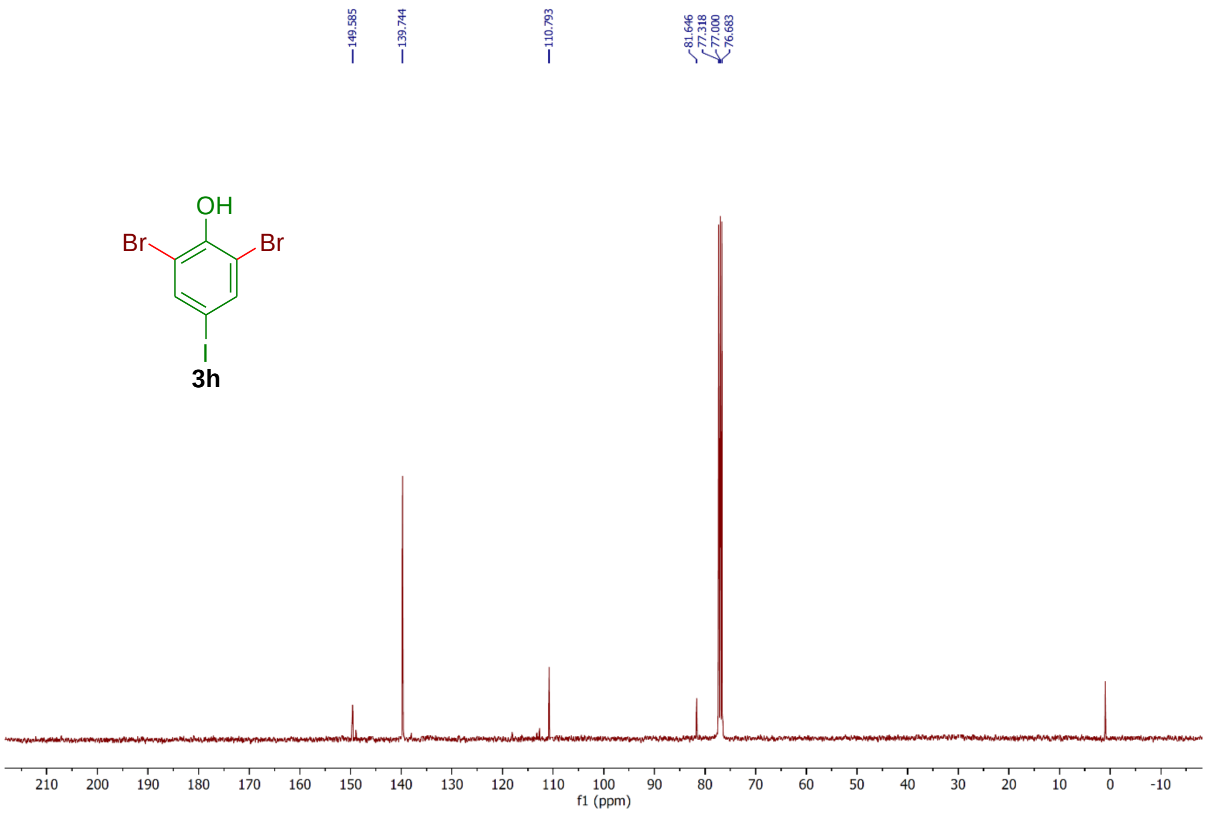

Figure S88: ^{13}C NMR spectrum of compound **3h**, ($CDCl_3$, 100 MHz).

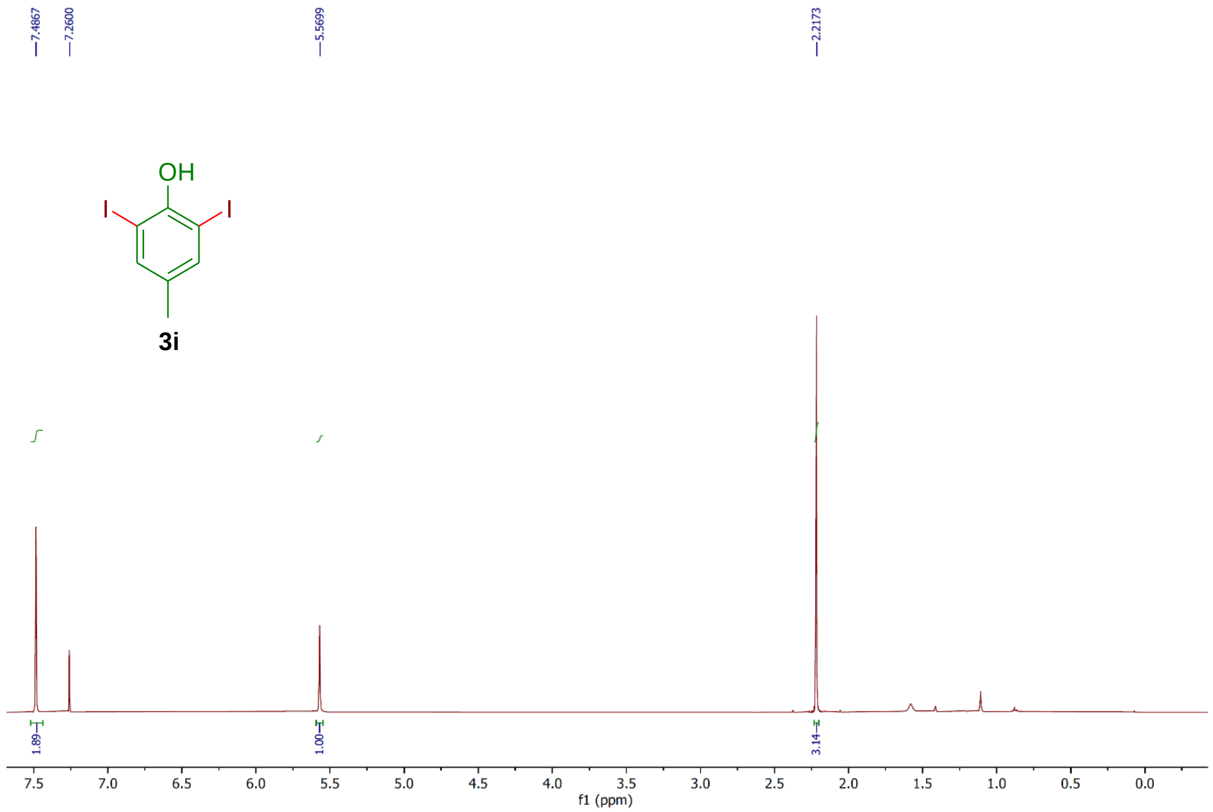

Figure S89: ^{1}H NMR spectrum of compound **3i**, ($CDCl_3$, 400 MHz).

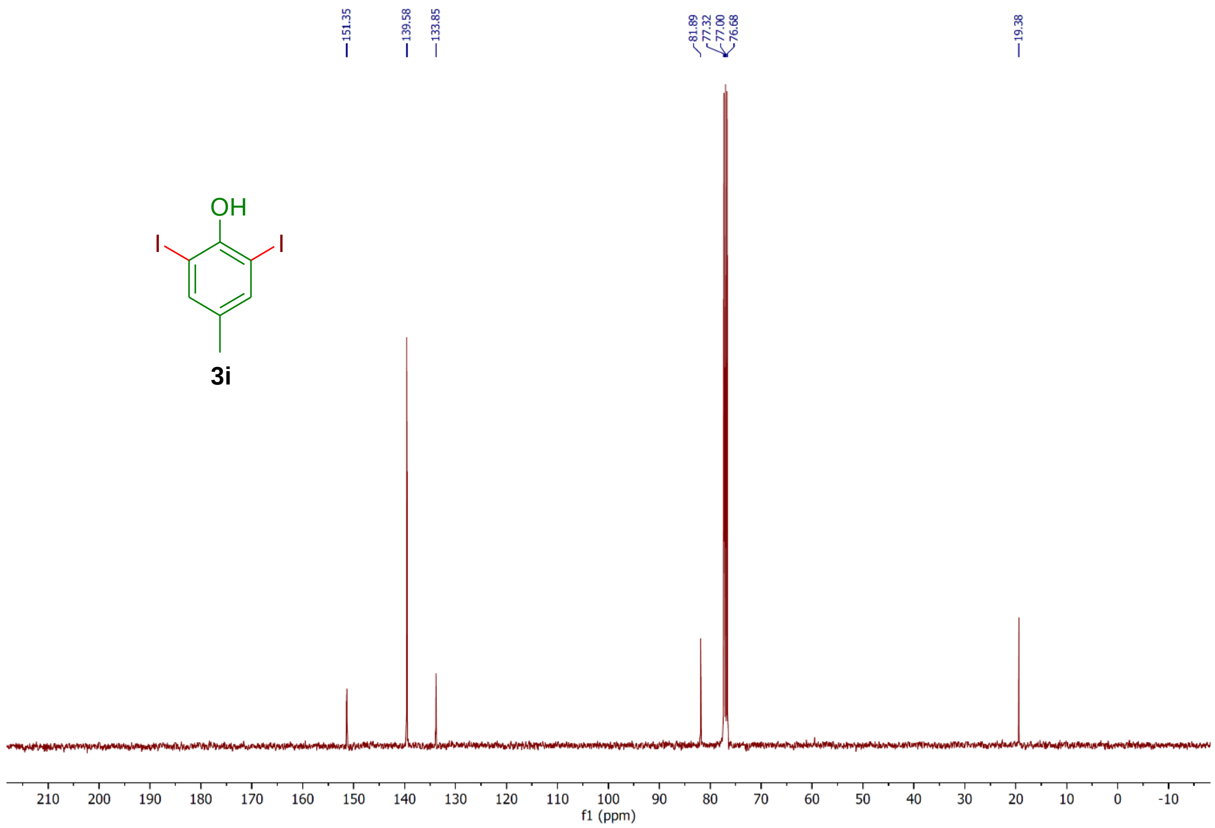

Figure S90: ^{13}C NMR spectrum of compound **3i**, ($CDCl_3$, 100 MHz).

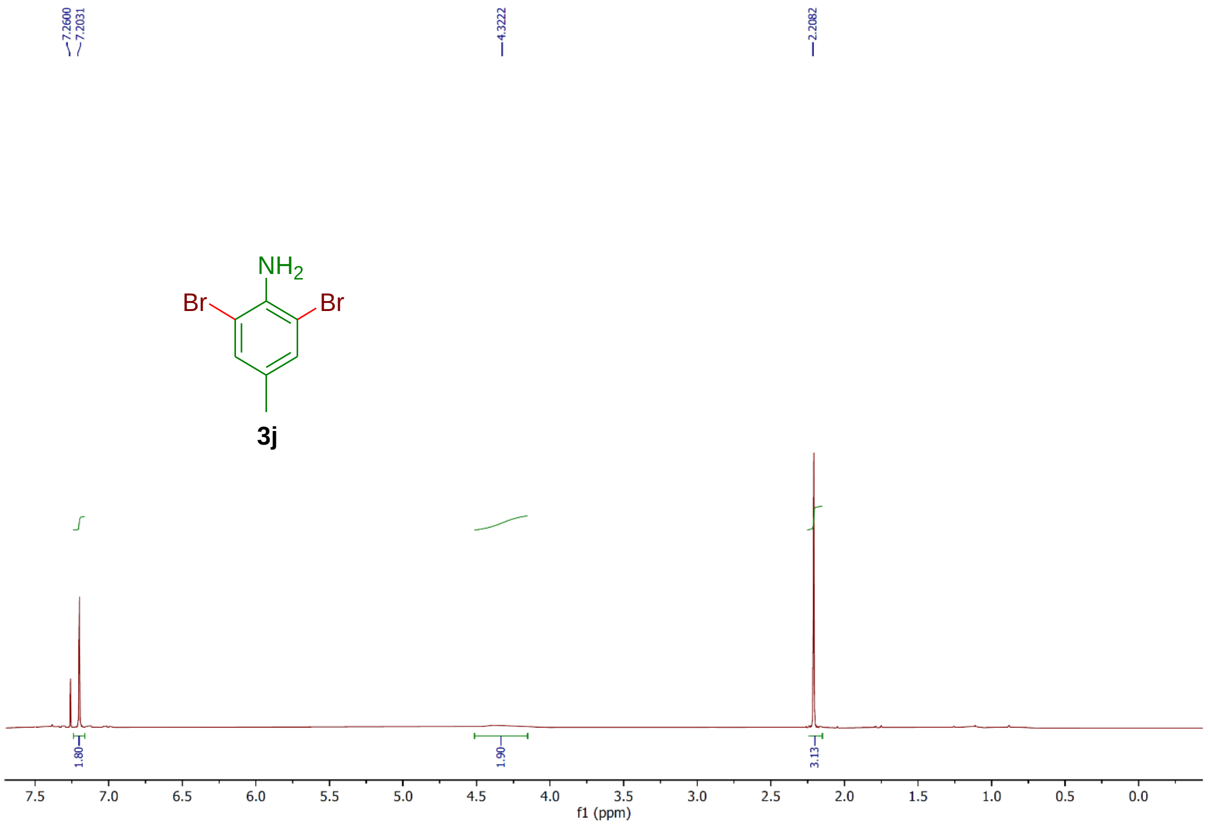

Figure S91: ^{1}H NMR spectrum of compound **3j**, ($CDCl_3$, 400 MHz).

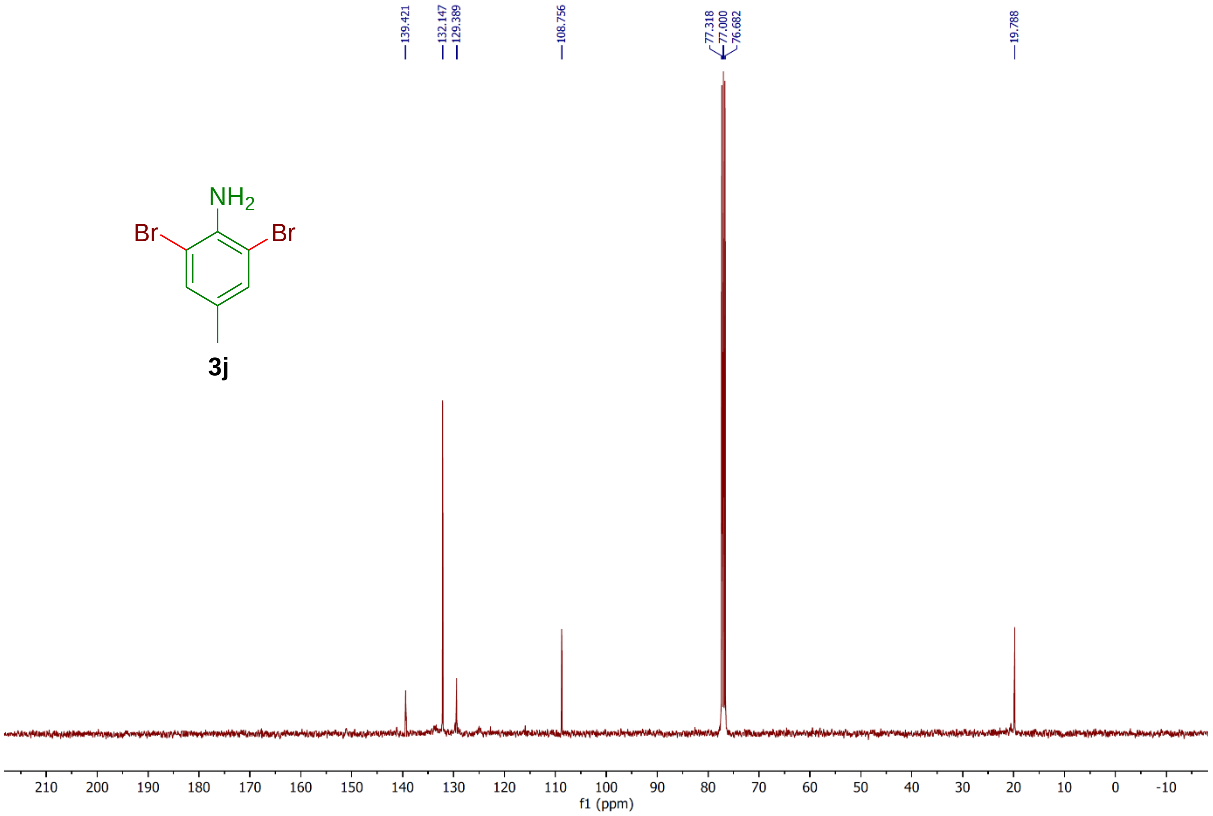

Figure S92: ^{13}C NMR spectrum of compound **3j**, ($CDCl_3$, 100 MHz).

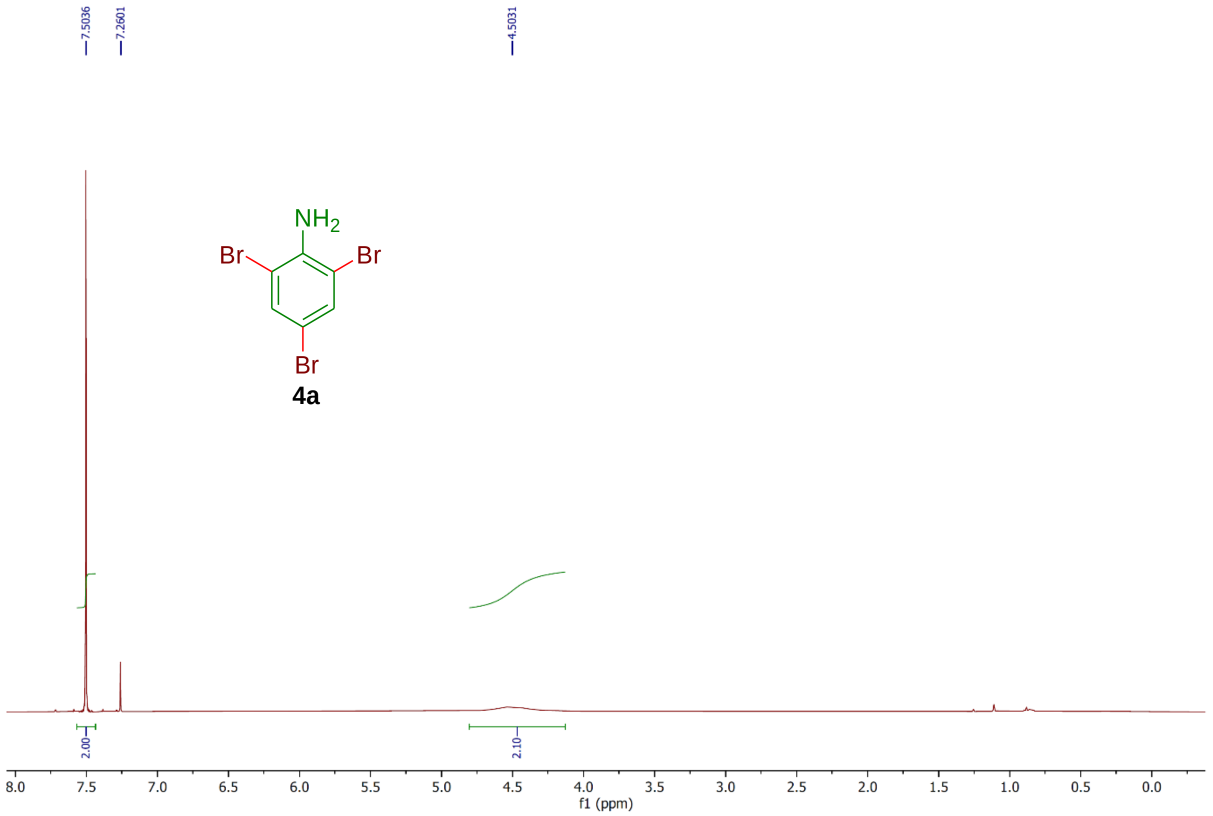

Figure S93: ^{1}H NMR spectrum of compound **4a**, ($CDCl_3$, 400 MHz).

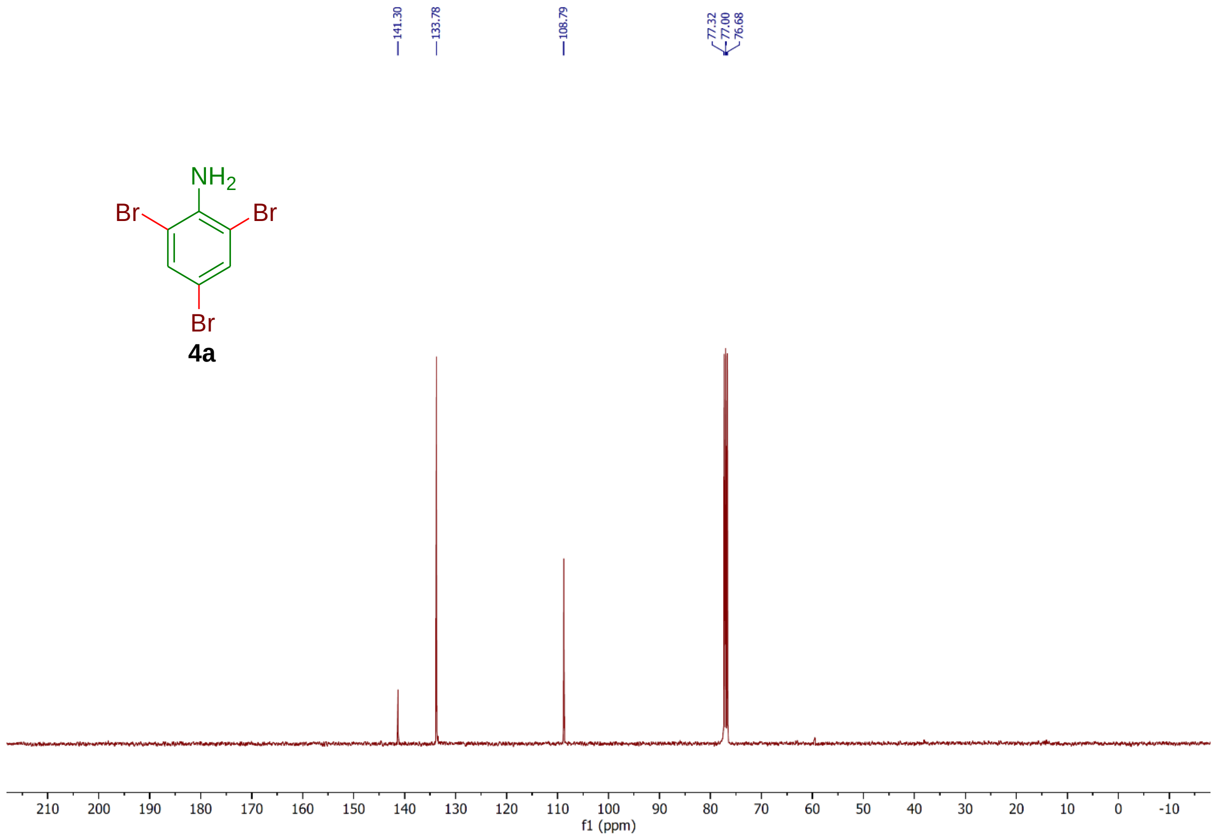

Figure S94: ^{13}C NMR spectrum of compound **4a**, ($CDCl_3$, 100 MHz).

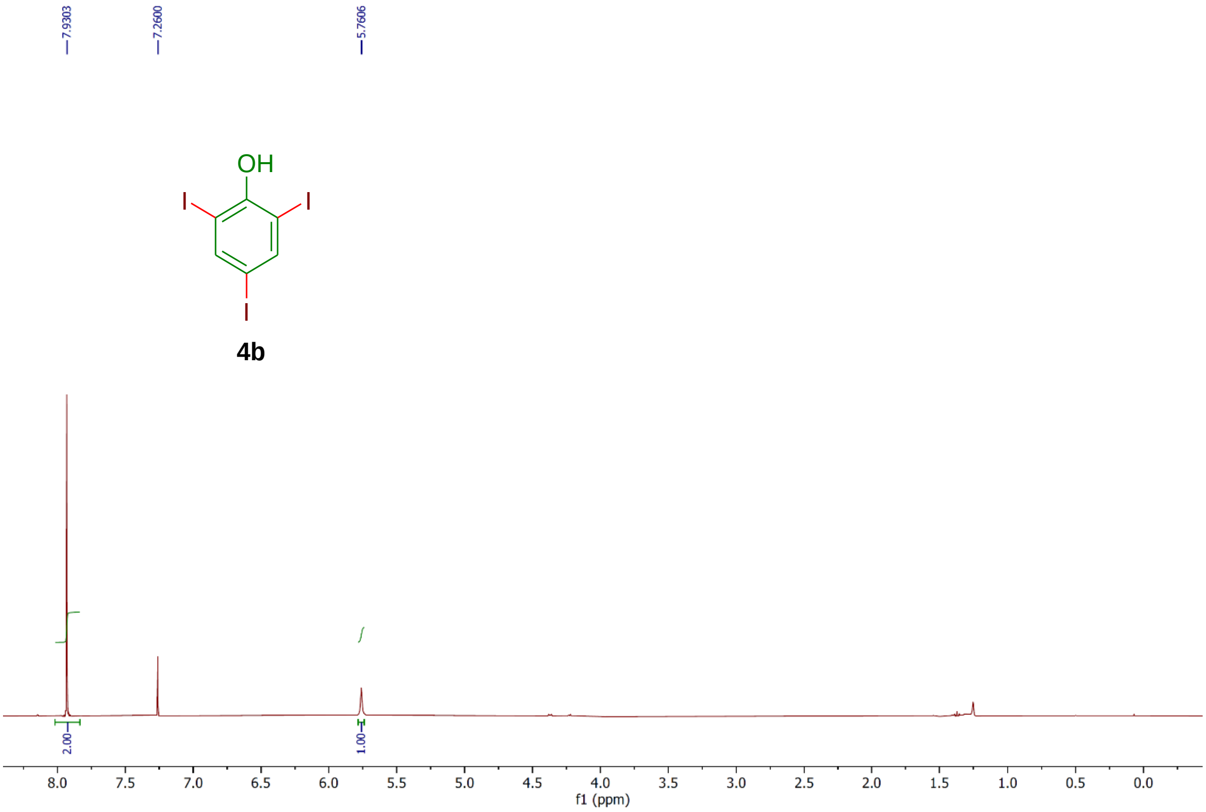

Figure S95: ^{1}H NMR spectrum of compound **4b**, ($CDCl_3$, 400 MHz).

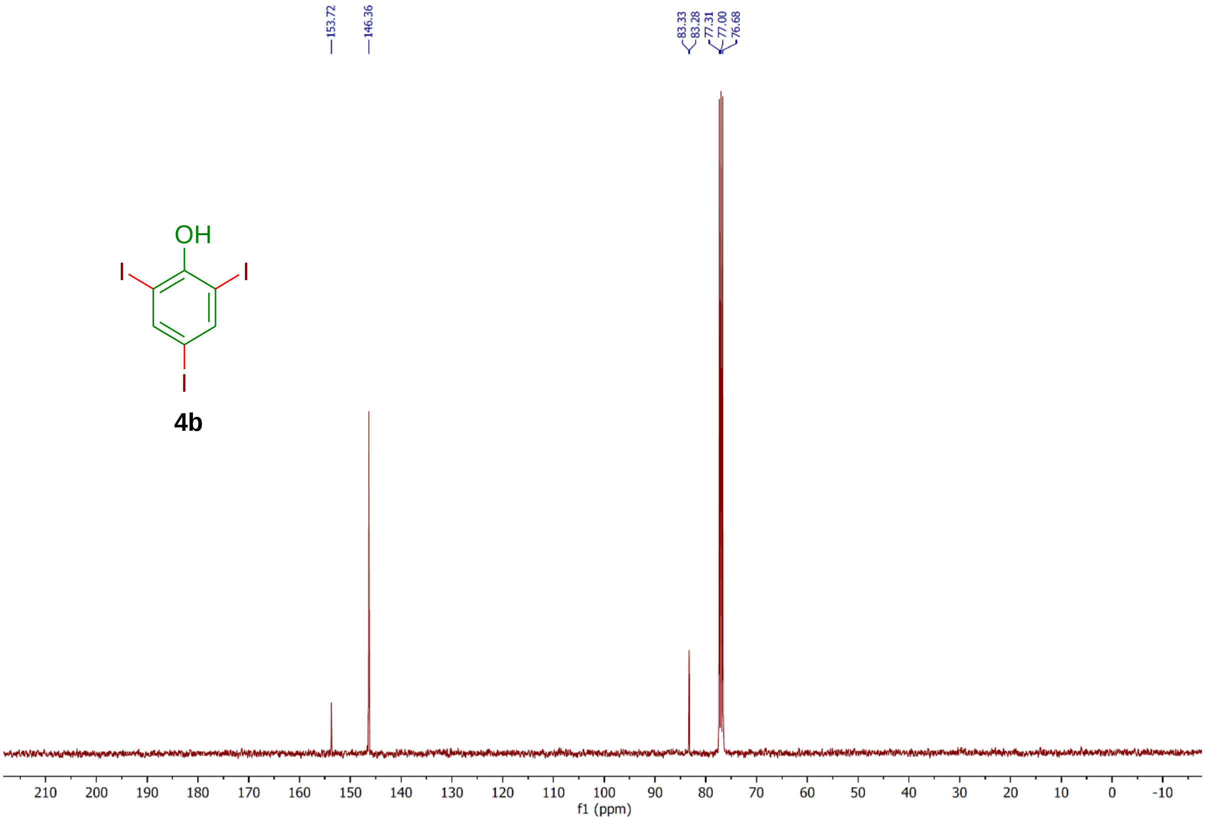

Figure S96: ^{13}C NMR spectrum of compound **4b**, ($CDCl_3$, 100 MHz).

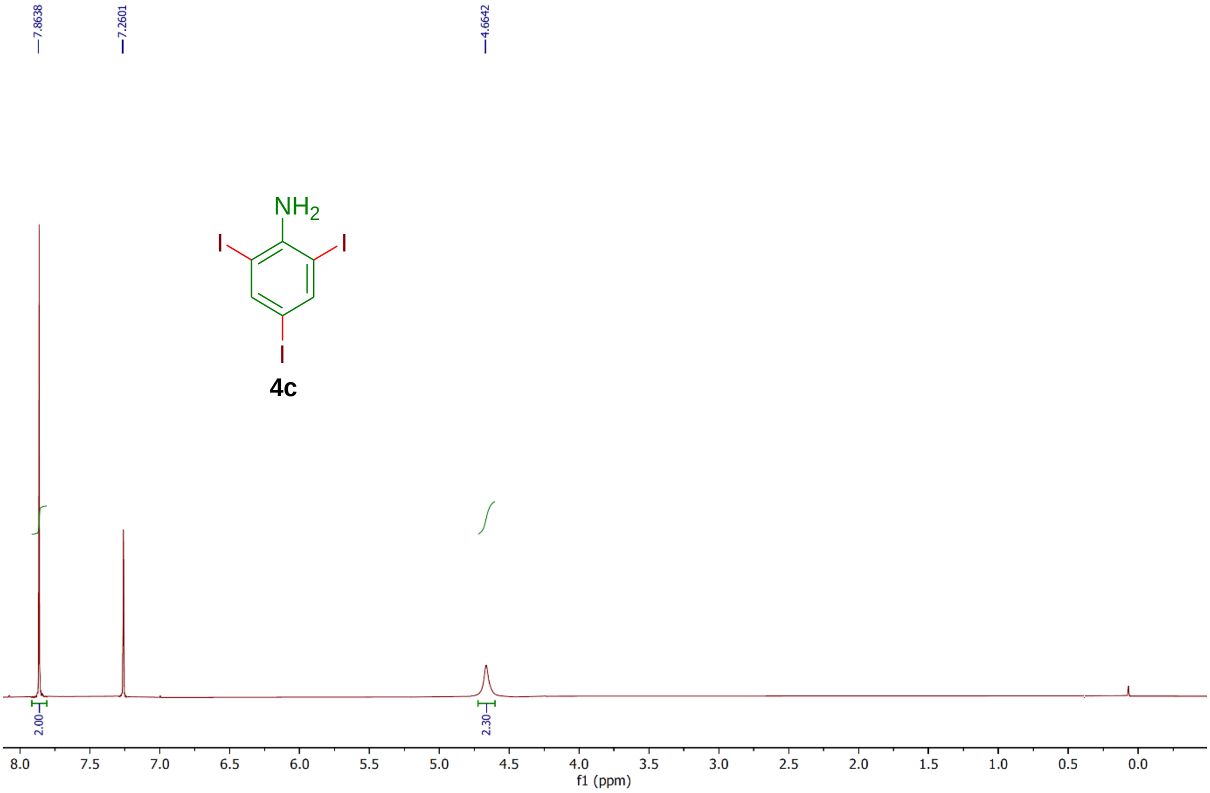

Figure S97: ^{1}H NMR spectrum of compound **4c**, ($CDCl_3$, 400 MHz).

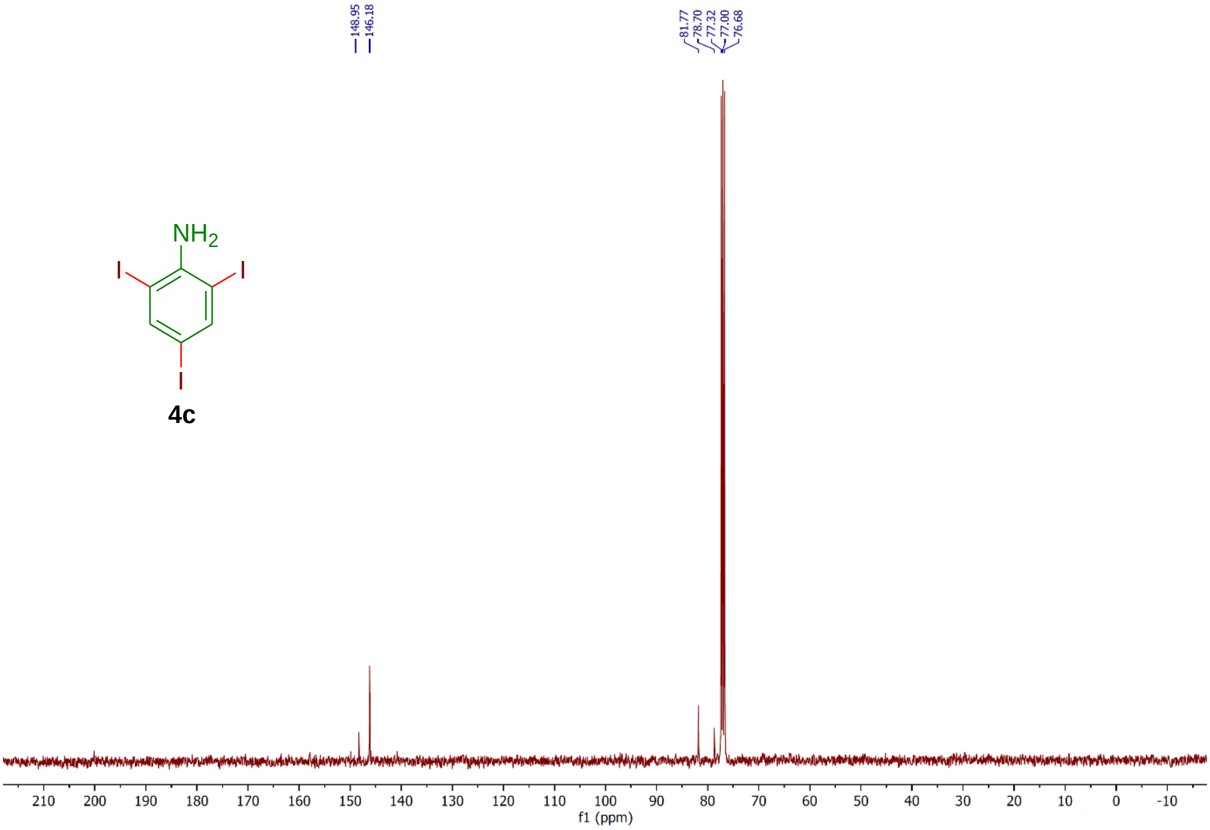

Figure S98: ^{13}C NMR spectrum of compound **4c**, ($CDCl_3$, 100 MHz).

References

1. Wang, L.; Feng, C.; Zhang, Y.; Hu, J. *Molecules* **2020**, *25*, 914. doi:10.3390/molecules25040914
2. Tang, R.-J.; Milcent, T.; Crousse, B. *J. Org. Chem*. **2018**, *83*, 930−938. doi:10.1021/acs.joc.7b02920
3. Otomatsu, T.; Bai, L. M.; Fujita, N.; Shindo, K.; Shimizu, K.; Misawa, N. *J. Mol. Catal. B Enzym.* **2010**, *66*, 234–240. doi:10.1016/j.molcatb.2010.05.015
4. Boruah, J. J.; Das, S. P.; Borah, R.; Gogoi, S. R.; Islam, N. S. *Polyhedron* **2013**, *52*, 246−254. doi:10.1016/j.poly.2012.09.036
5. Schmidt, B.; Holter, F. *Org. Biomol. Chem*. **2011**, *9*, 4914−4920. doi:10.1039/c1ob05256j
6. Eisenhauer, H. R.; Link, K. P. *J. Am. Chem. Soc.* **1954**, *76*, 6, 1647–1649. doi: 10.1021/ja01635a054
7. Ma, X.; Yu, J.; Jiang, M.; Wang, M.; Tang, L.; Wei, M.; Zhou, Q. *Eur. J. Org. Chem*. **2019**, *28*, 4593–4596. doi:10.1002/ejoc.201900794ax
8. Clewley, R. G.; Cross, G. G.; Fischer, A. Henderson, G. N. *Tetrahedron*, **1989**, *45*, 1299–1310. doi: 10.1016/0040-4020(89)80128-0
9. Denton, R. M.; An, J.; Lindovska, P.; Lewis, W. *Tetrahedron*, **2012**, *68,* 2899–2905. doi:10.1016/j.tet.2012.01.067
10. Popa, M. M.; Draghici, C.; Barbu, L.; Dumitrescu, D. E.; Dumitrascu, F. *Synth. Commun.* **2017**, *47,* 344–350. doi:10.1080/00397911.2016.1264603
11. Vijaykumar, G.; Mandal, S. K. *Dalton Trans*. **2016**, *45*, 7421−7426. doi:10.1039/c6dt00470a
12. Le, Z. G.; Chen, Z. C.; Hu, Y.; Zheng, Q. G. *Synthesis* **2004**, *17*, 2809−2812. doi:10.1055/s-2004-831239
13. Ageshina, A. A.; Chesnokov, G. A.; Topchiy, M. A.; Alabugin, I. V.; Nechaev, M. S.; Asachenko, A. F. *Org. Biomol. Chem*. **2019**, *17*, 4523−4534. doi:10.1039/c9ob00615j
14. Khalifa, A.; Conway, L.; Geoghegan, K.; Evans, P. *Tetrahedron Lett*. **2017**, *58*, 4559–4562. doi:10.1016/j.tetlet.2017.10.053
15. Zhou, Y.; Zhou, H.; Liu, S.; Pi, D.; Shen, G. *Tetrahedron* **2017**, *73*, 3898– 3904. doi:10.1016/j.tet.2017.05.056
16. Wu, Y.; Xu, S.; Wang, H.; Shao, D.; Qi, Q.; Lu, Y.; Ma, L.; Zhou, J.; Hu, W.; Gao, W.; Chen, J. *J. Org. Chem.* **2021**, *86*, 16144–16150. doi: 10.1021/acs.joc.1c00923

17. Zhou, Z.; Ma, Z.; Behnke, N. E.; Gao, H.; Kürti, L. *J. Am. Chem. Soc*. **2017**, *139*, 115−118. doi: 10.1021/jacs.6b12712

18. Syvret, R. G.; Butt, K. M.; Nguyen, T. P.; Bulleck, V. L.; Rieth, R. D. *J. Org. Chem*. **2002**, *67*, 4487–4493. doi:10.1021/jo020053u

19. Satkar, Y.; Yera-Ledesma, L. F.; Mali, N.; Patil, D.; Navarro-Santos, P.; Segura-Quezada, L. A.; Ramírez-Morales, P. I.; Solorio-Alvarado, C. R. *J. Org. Chem.* **2019**, *84*, 4149–4164. doi:10.1021/acs.joc.9b00161

20. Rao, A. S.; Ramesh, K.; Rajanna, K. C.; Chakrvarthi, I. *Asian. J. Chem.* **2018**, *30*, 1892–1896. doi:10.14233/ajchem.2018.21413

21. Moorthy, J. N.; Senapati, K.; Kumar, S. *J. Org. Chem.* **2009**, *74*, 6287–6290. doi:10.1021/jo9007892

22. Rong, N.; Yuan, Y.; Chen, H.; Yao, C.; Li, T.; Wanga, Y.; Yang, W. *Org. Chem. Front*., **2021**, *8*, 4479–4484. doi:10.1039/d1qo00461a

23. Racys, D. T.; Sharif, S. A. I.; Pimlott, S. L.; Sutherland, A. *J. Org. Chem*. **2016**, *81*, 772–780. doi:10.1021/acs.joc.5b02761

24. Shinde, A. T.; Zangade, S. B.; Chavan, S. B.; Vibhute, A. Y.; Nalwar, Y. S.; Vibhute, Y. B. *Synth. Commun*. **2010**, *40*, 3506–3513. doi:10.1080/00397910903457332

25. Tavana, M.; Montazeri, N.; Imanzadeh, G. *Asian J. Chem.* **2011**, *23*, 3097–3100.

26. Vibhute, Y. B.; Lonkar, S. M.; Sayyed, M. A.; Baseer, M. A. *Mendeleev Commun.* **2007**, *17*, 51. doi:10.1016/j.mencom.2007.01.020.

27. Kress, T. J.; Moore, L. L.; Costantino, S. M. *J. Org. Chem.* **1976**, *41*, 93−96. doi:10.1021/jo00863a020

28. Satkar, Y.; Ramadoss, V.; Nahide, P. D.; García-Medina, E.; Juárez-Ornelas, K. A.; Alonso-Castro, A. J.; Chávez-Rivera, R.;´ Jiménez-Halla, J. O. C.; Solorio-Alvarado, C. R. *RSC Adv*. **2018**, *8*, 17806−17812. doi: 10.1039/c8ra02982b

29. Ghorpade, P. V.; Pethsangave, D. A.; Some, S.; Shankarling, G. S. *J. Org. Chem*. **2018**, 83, 7388–7397. doi:10.1021/acs.joc.8b00188

30. Mostafa, M. A. B.; Calder, E. D. D.; Racys, D. T.; Sutherland, A. *Chem. Eur. J.* **2017**, *23*, 1044−1047. doi:10.1002/chem.201605671

31. Rogers, D. A.; Brown, R. G.; Brandeburg, Z. C.; Ko, E. Y.; Hopkins, M. D.; LeBlanc, G.; Lamar, A. A. *ACS Omega* **2018**, *3*, 12868−12877. doi:10.1021/acsomega.8b02320

32. Chae, J.; Buchwald, S. L. *J. Org. Chem.* **2004**, *69*, 3336−3339. doi:10.1021/jo035819k

33. Kösel, T.; Dräger, G.; Kirschning, A. *Org. Biomol. Chem.* 2021, 19, 2907–2911. doi: 10.1039/d1ob00083g

34. Ferreira, I. M.; Casagrande, G. A.; Pizzuti, L.; Raminelli, C. *Synth. Commun.* **2014**, 44, 2094–2102. doi:10.1080/00397911.2013.879900
35. Carreno, M. C.; Garcia Ruano, J. L.; Sanz, G.; Toledo, M. A.; Urbano, A. *J. Org. Chem.* **1995**, *60*, 5328−5331. doi:10.1021/jo00121a064
36. Zysman-Colman, E.; Arias, K.; Siegel, J. S. *Can. J. Chem*. **2009**, *87*, 440−447. doi:10.1139/v08-176
37. Bovonsombat, P.; Ali, R.; Khan, C.; Leykajarakul, J.; Pla-on, K.; Aphimanchindakul, S.; Pungcharoenpong, N.; Timsuea, N.; Arunrat, A.; Punpongjareorn, N. *Tetrahedron* **2010**, *66*, 6928–6935. doi:10.1016/j.tet.2010.06.041
38. Bovonsombat, P.; Teecomegaet, P.; Kulvaranon, P.; Pandey, A.; Chobtumskul, K.; Tungsirisurp, S.; Sophanpanichkul, P.; Losuwanakul, S.; Soimaneewan, D.; Kanjanwongpaisan, P.; Siricharoensang, P.; Choosakoonkriang, S. *Tetrahedron* **2017**, *73*, 6564–6572. doi:10.1016/j.tet.2017.10.005
39. Pramanick, P. K.; Hou, Z.-L.; Yao, B. *Tetrahedron* **2017**, *73*, 7105–7114. doi:10.1016/j.tet.2017.10.073
40. Du, B.; Jiang, X.; Sun, P. *J. Org. Chem.* **2013**, *78*, 6, 2786–2791. doi: 10.1021/jo302765g
41. Schröder, N.; Wencel-Delord, J.; Glorius, F. *J. Am. Chem. Soc*. **2012**, *134*, 8298−8301. doi:10.1021/ja302631j
42. Xu, H.; Hu, L.; Zhu, G.; Zhu Y.; Wang, Y.; Zheng-Guang Wu, Z.-G.; Zi, Y.; Huang, W. *RSC Adv.* **2022**, *12*, 7115–7119. doi: 10.1039/d2ra00197g
43. Bose, A.; Mal, P. *Tetrahedron Lett*. **2014**, *55*, 2154–2156. doi:10.1016/j.tetlet.2014.02.064
44. Ghanbarin, N.; Ghafuri, H.; Zand, H. R. E.; Eslami, M. *SynOpen*, 2017, **1**, 143–146. doi:10.1055/s-0036-1590959
